# Causal association between gut microbiota and fibromyalgia: a Mendelian randomization study

**DOI:** 10.3389/fmicb.2023.1305361

**Published:** 2024-01-08

**Authors:** Zhaohua Wang, Dan Jiang, Min Zhang, Yu Teng, Yaojiang Huang

**Affiliations:** ^1^Beijing Engineering Research Center of Food Environment and Public Health, Minzu University of China, Beijing, China; ^2^College of Food Science and Engineering, Dalian Ocean University, Dalian, China; ^3^College of life and Environmental Science, Minzu University of China, Beijing, China

**Keywords:** gut microbiota, fibromyalgia, Mendelian randomization, causal association, gut-brain axis

## Abstract

**Background:**

Fibromyalgia (FM) is a syndrome characterized by chronic and widespread musculoskeletal pain. A number of studies have implied a potential association between gut microbiota and FM. However, the casual association between gut microbiota and FM remains unknown.

**Method:**

Mendelian randomization (MR) study was conducted using the summary statistics of genetic variants from the genome-wide association study (GWAS). Inverse variance weighted (IVW), combined with MR-Egger and weighted median were used to investigate the causal association between 119 gut microbiota genera and FM. Sensitivity analyses were performed on the MR results, including heterogeneity test, leave-one-out test and pleiotropy test.

**Results:**

A total of 1,295 single nucleotide polymorphism (SNPs) were selected as instrumental variables (IVs), with no significant heterogeneity and pleiotropy according to the sensitivity analyses. Five gut microbiota genera were found to have significant casual association with FM. *Coprococcus*2 (OR = 2.317, *p*-value = 0.005, 95% CI: 1.289–4.167), *Eggerthella* (OR = 1.897, *p*-value = 0.001, 95% CI: 1.313–2.741) and *Lactobacillus* (OR = 1.576, *p*-value =0.020, 95% CI: 1.073–2.315) can increase the risk of FM. FamillyXIIIUCG001 (OR = 0.528, *p*-value = 0.038, 95% CI: 0.289–0.964) and *Olsenella* (OR = 0.747, *p*-value = 0.050, 95% CI: 0.557–1.000) can decrease the risk of FM.

**Conclusion:**

This MR study found that gut microbiota is casually associated with FM. New insights into the mechanisms of FM mediated by gut microbiota are provided.

## 1 Introduction

Fibromyalgia (FM) is an enigmatic syndrome characterized by chronic and widespread musculoskeletal pain. The global prevalence of FM is estimated at 2.7%, and is more in women than men (Sarzi-Puttini et al., 2020). Fibromyalgia can have an extremely negative impact on the psychological and physical condition of patients, causing a decreased quality of their life (Favretti et al., 2023). However, FM remains poorly understood, lacking effective preventive and therapeutic strategies. Multiple factors have been explored in the development of FM, and the gut-brain axis and gut microbiota are proposed as a promising mechanism (Giorgi et al., 2023).

The gut-brain axis is getting increasingly focused. It is a bidirectional interaction between the central and enteric nervous systems, involving multiple pathways including immune, neural, endocrine, and metabolic routes (Mayer et al., 2022). And the gut microbiota, a diverse and dynamic community of microorganisms that inhabit the digestive tract, is considered a key factor that modifies the gut-brain axis (Guo et al., 2019; Ibrahim et al., 2022). It has been reported that gut microbiota is associated with rheumatic diseases (Yu et al., 2021; Zhao et al., 2022) and neurological diseases (Generoso et al., 2021; Socała et al., 2021; Chen et al., 2021b) and plays a critical role in their development. It has also been reported that gut microbiota is associated with FM (Minerbi et al., 2019; Minerbi and Fitzcharles, 2020), but this association is correlative rather than causal.

Mendelian randomization (MR) is a method that utilizes genetic variants as instrumental variables to assess the causal association between exposures and outcomes (Emdin et al., 2017). Compared with observational epidemiological studies, MR is inherently less prone to confounding and reverse causation because of the random allocation of genotypes in the forming of a zygote during gestation (Davey Smith and Hemani, 2014). It can generate robust results when assessing the causal effects of modifiable exposures on disease outcomes.

In this study, we used the genome-wide association study (GWAS) summary statistics, to conduct a MR study to investigate the causal association between gut microbiota and FM.

## 2 Materials and methods

### 2.1 Data sources

Genetic variants for gut microbiota were obtained from the GWAS summary statistic of MiBioGen consortium (MiBioGen; Kurilshikov et al., 2021). This dataset includes 18,340 samples from 24 cohorts. Microbiota quantitative trait loci mapping was performed to identify host genetic variants that were mapped to genetic loci associated with the abundance levels of gut microbiota. In this dataset, genus was the lowest taxonomic level, and 131 genera were identified, with 12 unknown genera (Kurilshikov et al., 2021). Therefore, 119 bacterial genera were included in this MR study for analysis. Genetic variants for FM were obtained from GWAS summary statistics of FinnGen consortium R9 release data (Kurki et al., 2023). This dataset includes 168,378 European participants consisted of 737 cases and 167,641 controls. Each participating cohort had previously obtained ethical approvals, so no additional ethical approvals or informed consents were required.

### 2.2 Instrumental variable selection

To identify the potential instrumental variables, the single nucleotide polymorphisms (SNPs) for each bacterial genus were screened based on the locus-wide significance threshold (*p* < 1.0×10^−5^). Then, to eliminate non-random associations between allelic variants at different loci, the linkage disequilibrium (LD) between these SNPs were calculated on the base of clumping window size = 10,000 kb and *R*^2^ < 0.001, using the European population as reference.

### 2.3 Two-sample and bidirectional MR

Multiple MR methods were used to investigate the causal association between each bacterial genus and FM, including inverse variance weighted (IVW), MR-Egger and weighted media.

We used the IVW method as the primary analysis. IVW is one of the most commonly used method in MR that uses the meta-analysis method to aggregate the Wald values of SNPs and derive an overall estimate of the effect (Burgess et al., 2013). In the absence of heterogeneity and horizontal pleiotropy, we prefer IVW to estimate the causal associations. MR-Egger is another commonly used method in MR that relaxes the assumption of no horizontal pleiotropy. Based on the Instrument strength independent of direct effect (InSIDE) assumption, it estimates the causal effect by fitting a regression model that includes the intercept term and the weighted average of the ratio estimates. However, incorporating the intercept term in regression correspondingly causes statistically less efficient estimates of causal effects (Bowden et al., 2015). When there is horizontal pleiotropy, the results obtained from the MR-Egger are preferred. The weighted median method is a robust method in MR that provides stable estimates even when up to 50% of the instruments are invalid (Hartwig et al., 2017). When there is heterogeneity but no horizontal pleiotropy present, the results obtained from the weighted median method are preferred.

Bidirectional MR was used to exclude the possible causal effect of FM on gut microbiota. The reverse MR analyses were only conducted for bacterial genera that were significantly causal associated with FM.

### 2.4 Sensitivity analysis

Sensitivity analyses were conducted on the MR results, including heterogeneity test, leave-one-out test, pleiotropy test.

To assess the heterogeneity of instrumental variables (IVs), the Cochran’s IVW *Q* statistics were calculated. If there is a *p*-value <0.05, it indicates the presence of heterogeneity. To assess the horizontal pleiotropy, the MR-Egger regression was used. If the intercept term has a significant deviation (*p*-value <0.05) from zero, it indicates the presence of horizontal pleiotropy. The MR-PRESSO analysis was also used to detect and reduce horizontal pleiotropy. It can detect and remove significant outliers (*p*-value <0.05) but it requires a minimum of 50% valid genetic variants and relies on the InSIDE assumptions (Verbanck et al., 2018). The Leave-one-out test was performed by excluding each of IV in turn to evaluate whether any of them exerted a disproportionate impact on the result. The *F*-statistic (Staiger and Stock, 1997) of each IV was calculated to evaluate their strength. IVs with *F*-statistic <10 were considered as weak instrumental variables, which may introduce weak instrumental bias to the result.

### 2.5 Statistical analysis

All statistical analyses were conducted using R version 4.3.0, using the TwoSampleMR (Hemani et al., 2018) and forestplot (Gordon and Lumley, 2022) R packages.

## 3 Results

**Figure 1:**
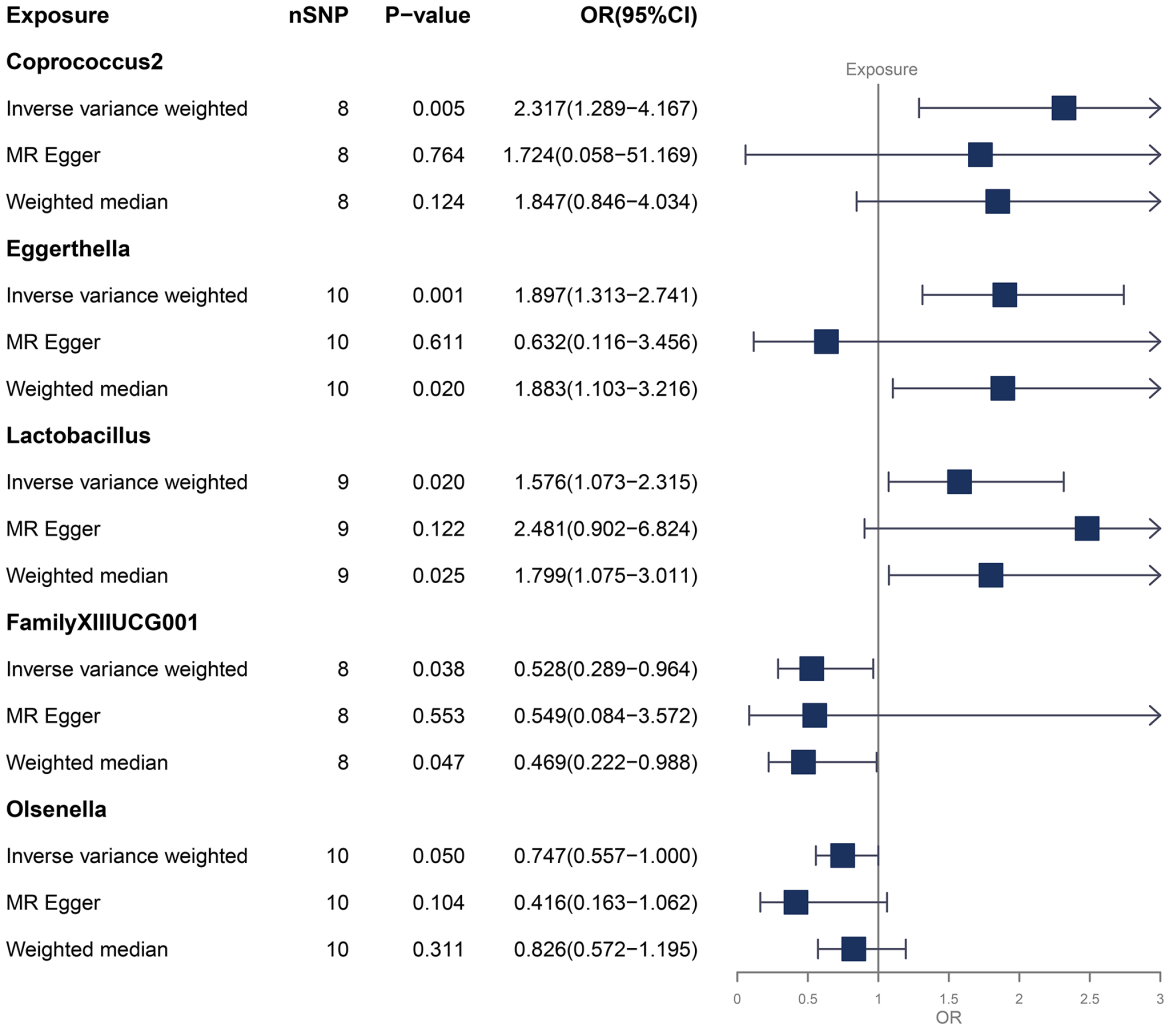
MR results for the causal association between gut microbiota and FM.

We identified a total of 1,295 SNPs as IVs for the gut microbiota, as is shown in Supplementary Table S1. Based on the MR results (Supplementary Table S2), we found that *Coprococcus*2, *Eggerthella*, *Lactobacillus*, FamilyXIIUC001 and *Olsenella* were causal associated with FM in at least one MR method (Figure 1).

We focused on the results of the IVW method, while considering the results of other methods as reference. As shown in Figure 1, *Coprococcus*2 (OR = 2.317, *p*-value = 0.005, 95% CI: 1.289–4.167), *Eggerthella* (OR = 1.897, *p*-value = 0.001, 95% CI: 1.313–2.741) and *Lactobacillus* (OR = 1.576, *p*-value =0.020, 95% CI: 1.073–2.315) could increase the risk of FM. FamillyXIIIUCG001 (OR = 0.528, *p*-value = 0.038, 95% CI: 0.289–0.964) and *Olsenella* (OR = 0.747, *p*-value = 0.050, 95% CI: 0.557–1.000) could decrease the risk of FM. The weighted median method also supported the result of *Eggerthella*, *Lactobacillus* and FamilyXIIIUCG001, while the weighted median method for *Coprococcus*2 and *Olsenella*, and the MR-Egger method did not find significant casual association, and their *p*-values were all greater than 0.1. Forest plots that reflected the single effect of IVs of these bacterial genera were shown in Supplementary Figure S1. In addition, the reverse MR analyses (Supplementary Table S3) did not find FM have any significant casual effect on gut microbiota.

**Figure 2:**
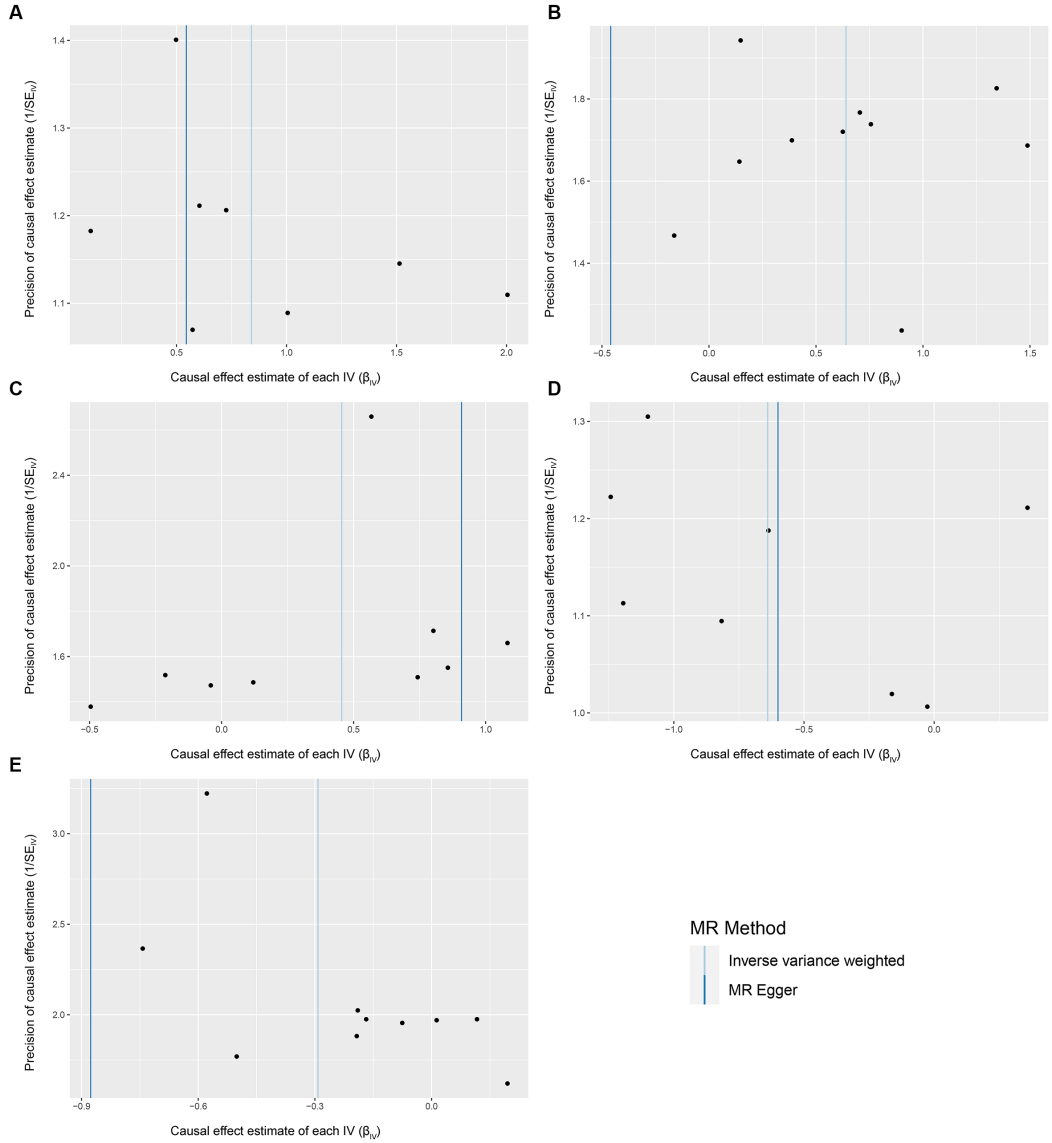
Funnel plots for the causal association between gut microbiota and FM. **(A)** *Coprococcus*2; **(B)** *Eggerthella*; **(C)** *Lactobacillus*; **(D)** FamillyXIIIUCG001; **(E)** *Olsenella*; funnel plots are scatter plots where the estimated causal effects of IVs are plotted against a measure of their precision. The *X*-axis represents the precision of the causal estimates, and the *Y*-axis represents the estimated causal effect of each genetic variant. In an unbiased scenario, where there is no heterogeneity, the scatter points on the funnel plot should exhibit a symmetric distribution resembling an inverted funnel shape.

**Figure 3:**
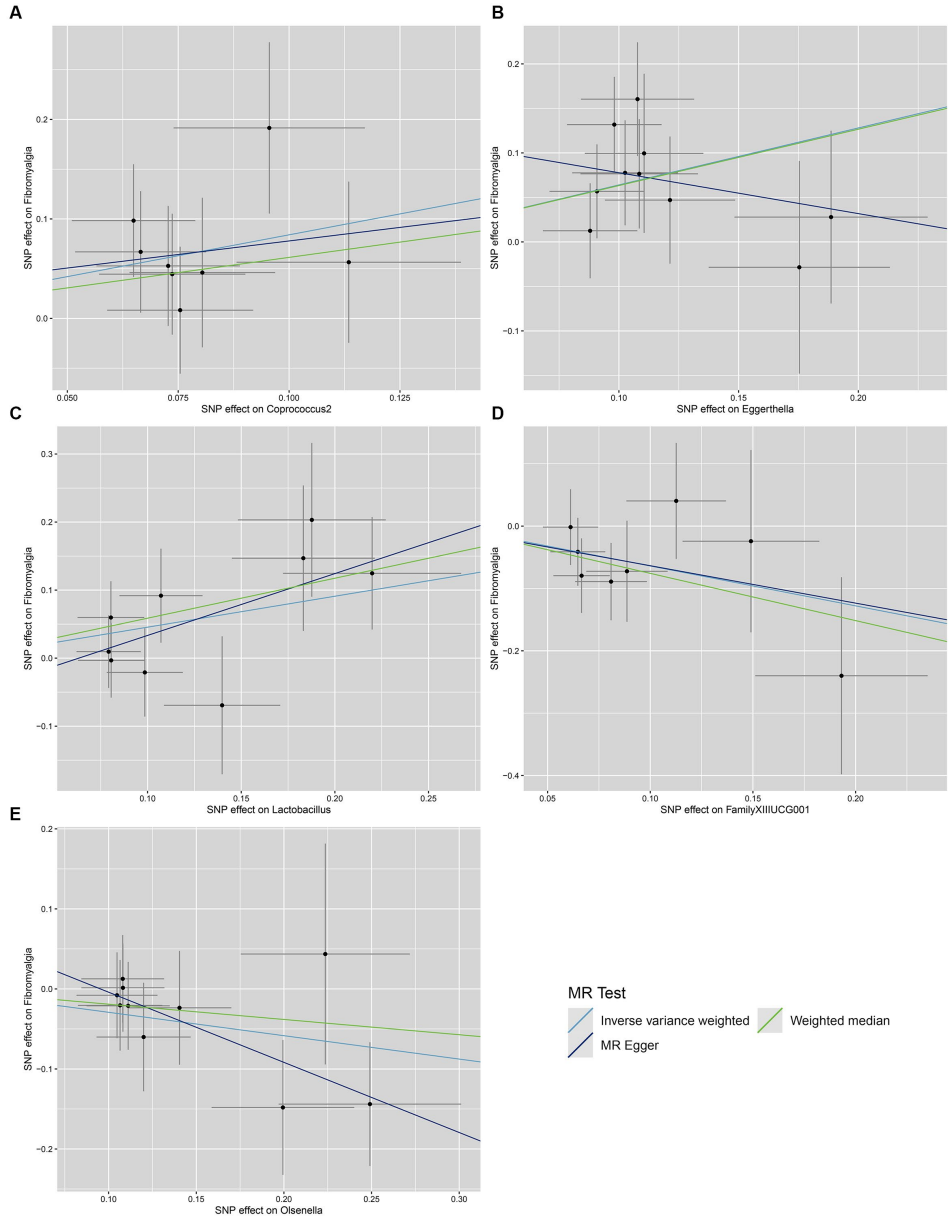
Scatter plots for the causal association between gut microbiota and FM. **(A)** *Coprococcus*2; **(B)** *Eggerthella*; **(C)** *Lactobacillus*; **(D)** FamillyXIIIUCG001; **(E)** *Olsenella*; scatter plots are used to visualize the association of IVs with exposure (gut microbiota) and outcome (FM). The *X*-axis and *Y*-axis, respectively, represent the effects of IVs on the exposure and outcome, with vertical and horizontal lines indicating the 95% confidence intervals for IVs. The slope of each line corresponding to the estimated MR effect of each method.

**Figure 4:**
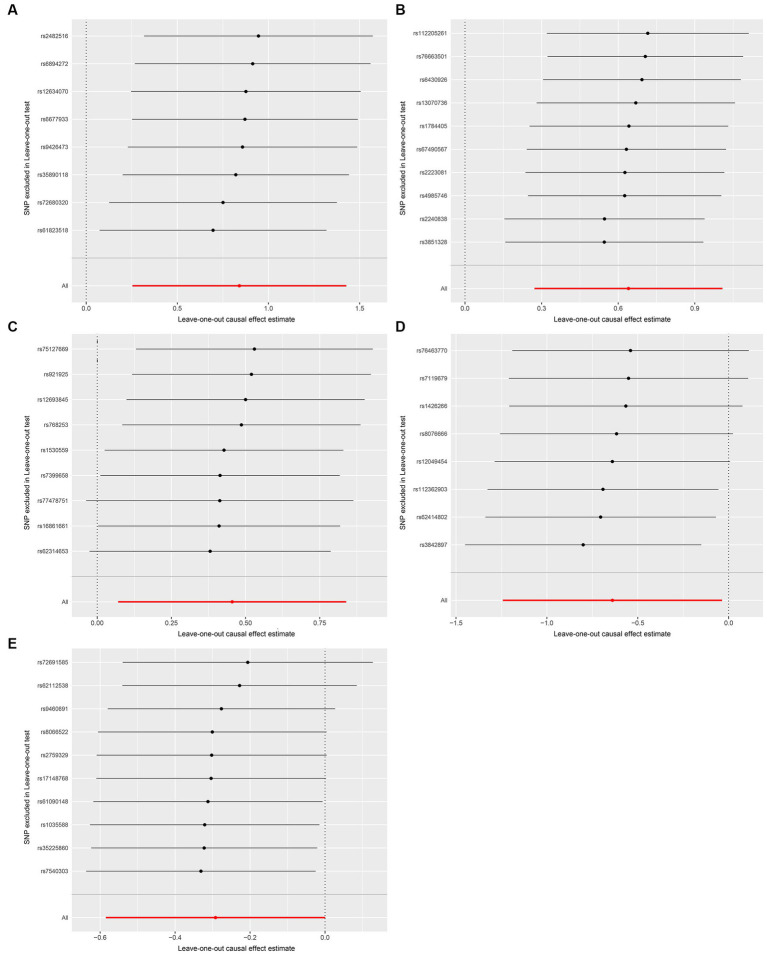
Leave-one-out plots for the causal association between gut microbiota and FM. **(A)** *Coprococcus*2; **(B)** *Eggerthella*; **(C)** *Lactobacillus*; **(D)** FamillyXIIIUCG001; **(E)** *Olsenella*; the leave-one-out plots are used to visualize the overall estimate of IVW (red horizontal line) and its estimates after the removal of each IV (black horizontal line).

We conducted a series of sensitivity analysis to ensure the reliability and robustness of the results. There was no significant heterogeneity of IVs as indicated by the non-significant results of Cochran’s IVW *Q* test (Supplementary Table S4). On the visual inspection of funnel plots (Figure 2), the MR-Egger method showed a presence of potential outliers, while the IVW method did not. And there was no significant horizontal pleiotropy according to the results from intercept analysis in the MR-Egger regression (Supplementary Table S5) and MR-PRESSO analysis (Supplementary Table S6). On the visual inspection of scatter plot (Figure 3), only the MR-Egger method for *Eggerthella* showed a significant deviation of the slopes and intercept term compared to the other two methods. The results of leave-one-out plots (Figure 4) were similar after excluding each IV from the study, which indicated that no single SNP had a disproportionate impact on the total results. The *F*-statistics of the IVs (Supplementary Table S7) were all greater than 10, indicating that the results are unlikely to be affected by weak instrumental bias.

## 4 Discussion

Gut microbiota is a crucial element of human health, intricately involved in various physiological functions within the human body (Zhang et al., 2020). Microbiota-gut-brain axis provide us a new way to understand FM. Potential associations between gut microbiota and FM has been identified, although these associations are correlative rather than causal (Clos-Garcia et al., 2019; Albayrak et al., 2021; Minerbi et al., 2022a,b). If a causal relationship between gut microbiota and FM is established, the way may be paved for the development of microbiota-based therapies and diagnostics for FM. In this study, we conducted a series of MR analyses to investigate the causal association between gut microbiota and FM, using the GWAS summary statistic from MiBioGen and FinnGen consortium. We found that *Coprococcus*2, *Eggerthella*, *Lactobacillus*, FamillyXIIIUCG001 and *Olsenella* were casually associated with FM.

*Coprococcus*2, *Eggerthella* and *Lactobacillus* were found to increase the risk of FM. *Coprococcus*2 is a bacterial genus producing short chain fatty acids (SCFAs), particularly butyric acid. Butyric acid and butyrate are often considered to maintain intestinal barrier function because it promotes the expression of mucus, antimicrobial peptides, and tight junction proteins (Garofalo et al., 2023). However, the effects of butyrate may be dose-dependent, as high concentrations of butyrate have been observed to induce intestinal cell apoptosis, thereby damage the intestinal barrier (Huang et al., 2014). It has also been observed an increase in the abundance of butyrate-producing bacteria and level of butyrate in patients with FM (Minerbi et al., 2019; Erdrich et al., 2020). In addition, high concentrations and mixtures of SCFAs can promote microglia activation and amplify the inflammatory process (Pender et al., 2000; Sampson et al., 2016). We proposed that it is the imbalance rather than deficiency of SCFAs that contribute to the development of FM. *Coprococcus*2 may increase the risk of FM by overproducing butyric acid.

Although there is a lack of research about the association between *Eggerthella* and FM, increased abundance of *Eggerthella* has been reported in various disorders, such as depression (Barandouzi et al., 2020; Liu et al., 2022), rheumatoid arthritis (Chen et al., 2021a), schizophrenia (Karpiński et al., 2023), systemic lupus erythematosus (He et al., 2016), and multiple sclerosis (Chang and Choi, 2023), all of which are common comorbidities of FM. *Eggerthella* can increase intestinal permeability and promote the production of pro-inflammatory cytokines (Salem et al., 2019). It can use ornithine as substrate to generate energy, producing citrulline and carbamyol phosphate as byproducts. And increased levels of citrulline in the gut participate in citrullination, leading to antibody production by the immune response (Chen et al., 2016). It can also activate Th17 cells, inducing an inflammatory response mediated by the Th17/Treg cell imbalance (Liu et al., 2022). These findings indicate the potential causal association between *Eggerthella* and FM.

*Lactobacillus* could increase the risk of FM. This is counterintuitive because *Lactobacillus* is regarded as a probiotic (Azad et al., 2018). In fact, the role of *Lactobacillus* is relatively heterogeneous. Certain species of *Lactobacillus* and have been shown to have potential association with FM. For example, *Lactobacillus plantarum* IS-10506 can upregulate serotonin (5-hydroxytryptamine, 5-HT) levels (Ranuh et al., 2019), while *Lactobacillus acidophilus* (Cao et al., 2018b) and *Lactobacillus rhamnosus* (Cao et al., 2018a) can downregulate 5-HT levels. Serotonergic dysfunction is one major hypothesis of fibromyalgia, which is supported by the efficacy of drugs that alter serotonin metabolism (Wolfe et al., 2013; Chinn et al., 2016). Another example, the *Lactobacillus ruminis* can stimulate the production of tumor necrosis factor (TNF) (Taweechotipatr et al., 2009). TNF can not only cross the blood-brain barrier but also increases its permeability (Banks et al., 1995; Tsao et al., 2001). TNF is also an activator of microglia (Klapal et al., 2016). We speculated that *Lactobacillus ruminis* may be involved in membrane dysfunction triggered by microglia through elevated levels of TNF, thus contributing to the development of FM. Unfortunately, due to the lack of statistic of genetic variant for bacterial species, we can only obtain the overall effect of the genus Lactobacillus.

FamilyXIIIUCG001 and *Olsenella* were found to decrease the risk of FM. There is limited research on these two bacterial genera, but we believe that they may be of significant importance for probiotic treatment of FM. In anhedonia-like phenotype rodents with neuropathic pain, the abundance of FamilyXIIIUCG001 were lower in susceptible group than in sham group and resilient group (Yang et al., 2019). In addition, in mice with chronic social defeat stress-induced depressive-like behavior receiving *Clostridium butyricum* miyairi 588 treatment, FamilyXIIIUCG001 was negatively correlated with depression-like behavior which is often comorbid with FM (Liu et al., 2014; Tian et al., 2019). As for *Olsenella*, a Mendelian randomization study has demonstrated that *Olsenella* can decrease the risk of Migraine (He et al., 2023). Another study found that *Olsenella* is negatively associated with the risk of low back pain (Su et al., 2023).

This study has several strengths. Firstly, we investigated the causal association between gut microbiota and FM through MR method, which can exclude the interference of confounding factors and reversing causation. Secondly, we obtained the summary statistics of genetic variants of diseases from the publicly available GWAS datasets, ensuring the reliability of IVs in the MR analysis. Thirdly, to account for bias related to study design, we performed several MR sensitivity analyses. Fourthly, we conducted reverse MR to assess the reversing causation, which could also contribute to the robustness of the study.

However, there are also several limitations in this study. Firstly, to perform sensitivity analysis, a broader range of genetic variations should be included as instrumental variables. Therefore, we did not use the traditional GWAS significance threshold (*p* < 5E–8) to screen SNPs. Secondly, the smallest taxonomic level in the original GWAS study is the genus level. Therefore, we failed to investigate the causal association with FM of gut microbiota at species level, which can be more accurate. Further research is needed to obtain the GWAS statistics of genetic variants for bacterial species. Thirdly, most of participants in these GWAS were European. This leads to the concern that our results may not be entirely applicable to other descent.

## 5 Conclusion

This MR study found that gut microbiota, including *Coprococcus*2, *Eggerthella*, *Lactobacillus*, FamillyXIIIUCG001 and *Olsenella* were casually associated with FM. It could contribute to the development of effective preventive and therapeutic strategies for FM.

## Data availability statement

The datasets presented in this study can be found in online repositories. The names of the repository/repositories and accession number(s) can be found in the article/Supplementary material.

## Ethics statement

Ethical approval was not required for the study involving humans in accordance with the local legislation and institutional requirements. Written informed consent to participate in this study was not required from the participants or the participants’ legal guardians/next of kin in accordance with the national legislation and the institutional requirements.

## Author contributions

ZW: Data curation, Formal analysis, Investigation, Methodology, Software, Visualization, Writing – original draft, Writing – review & editing. DJ: Data curation, Formal analysis, Validation, Writing – original draft, Writing – review & editing. MZ: Validation, Writing – review & editing. YT: Validation, Writing – review & editing. YH: Conceptualization, Funding acquisition, Project administration, Resources, Supervision, Writing – review & editing.
