# Supplementary material for: Causal association between gut microbiota and fibromyalgia: a Mendelian randomization study
Source: Front Microbiol. 2024 Jan 8;14:1305361. doi: 10.3389/fmicb.2023.1305361 (PMC10800605; doi:10.3389/fmicb.2023.1305361)
Supplement: Supplementary file 1 [file Data_Sheet_1.docx]

Supplementary Material

# Supplementary Figures and Tables

## Supplementary Figures


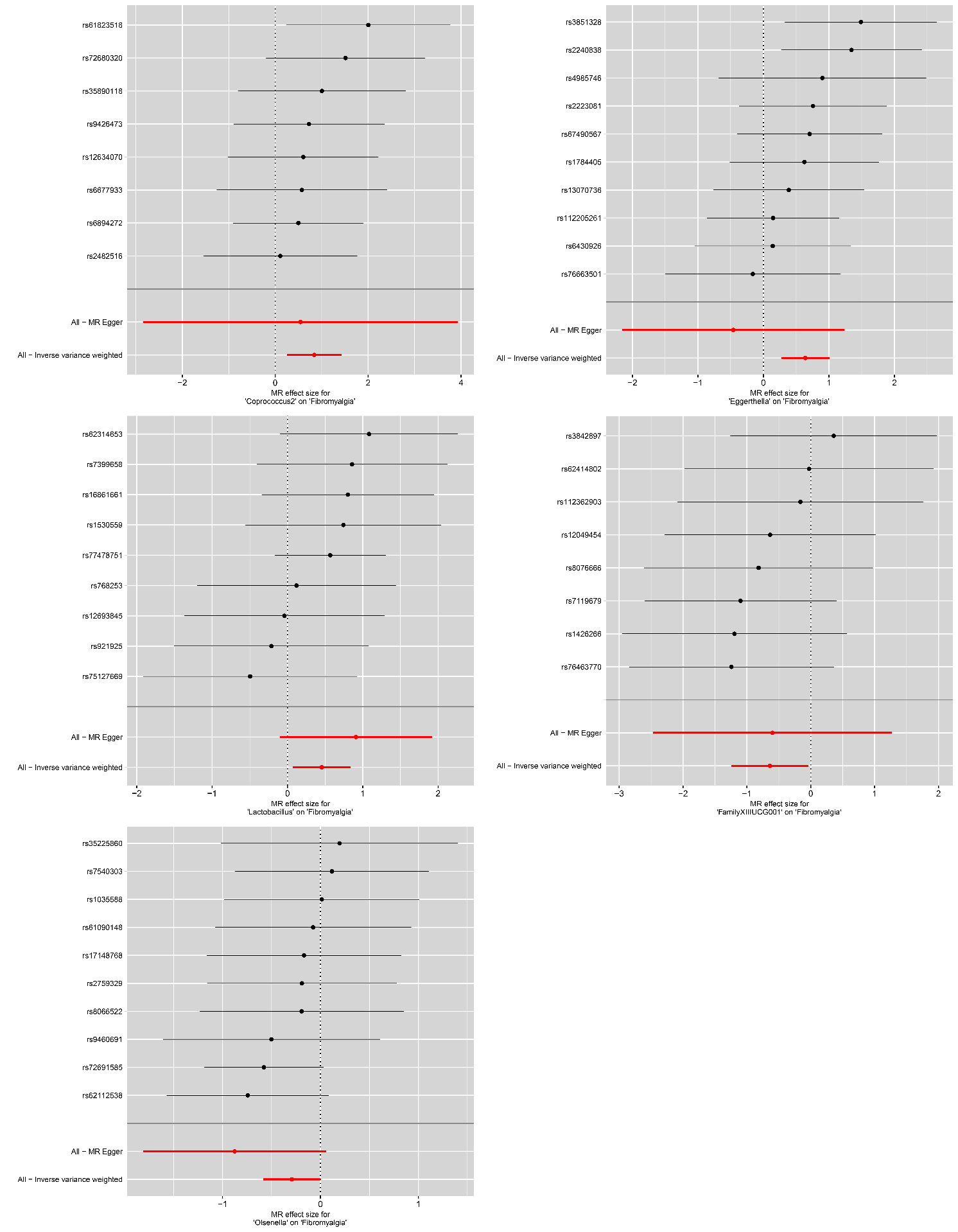


**Supplementary Figure 1.** Forest plots of the causal effects of single IV for gut microbiota on FM. The significance of red lines are MR results of MR-Egger regression and IVW method. (A) Osteoarthritis, (B) Rheumatoid arthritis, (C) Depression, (D) Sleeplessness.

## Supplementary Table

**Supplementary Table 1.** Instrumental variables used in MR analysis of the association between gut microbiota and FM

| Bacterial taxa (exposure) | SNP | Effect allele | Other allele | Exposure (Bacteria) | | | Outcome (FM) | | |
| --- | --- | --- | --- | --- | --- | --- | --- | --- | --- |
|  |  |  |  | Beta | SE | P-value | Beta | SE | P-value |
| Actinomyces | rs34583783 | G | T | 0.127 | 0.027 | 2.41E-06 | -0.053 | 0.111 | 0.636 |
| Actinomyces | rs7915461 | T | C | 0.188 | 0.040 | 2.94E-06 | -0.065 | 0.104 | 0.532 |
| Actinomyces | rs4146653 | G | A | 0.099 | 0.021 | 4.23E-06 | -0.025 | 0.076 | 0.741 |
| Actinomyces | rs35011108 | A | G | 0.233 | 0.051 | 5.54E-06 | 0.032 | 0.106 | 0.762 |
| Actinomyces | rs2715439 | C | T | 0.075 | 0.016 | 5.89E-06 | -0.067 | 0.053 | 0.209 |
| Actinomyces | rs4073240 | G | A | 0.075 | 0.017 | 7.49E-06 | -0.006 | 0.055 | 0.910 |
| Actinomyces | rs71315246 | A | G | -0.097 | 0.022 | 9.72E-06 | -0.087 | 0.077 | 0.260 |
| Adlercreutzia | rs7680684 | C | T | -0.083 | 0.017 | 7.95E-07 | 0.115 | 0.056 | 0.038 |
| Adlercreutzia | rs34181676 | G | T | -0.135 | 0.028 | 1.74E-06 | -0.213 | 0.132 | 0.108 |
| Adlercreutzia | rs9490822 | C | T | -0.073 | 0.016 | 2.42E-06 | 0.044 | 0.053 | 0.405 |
| Adlercreutzia | rs13231526 | C | A | 0.143 | 0.031 | 4.31E-06 | 0.147 | 0.098 | 0.131 |
| Adlercreutzia | rs6664405 | T | C | -0.095 | 0.021 | 6.12E-06 | -0.012 | 0.076 | 0.878 |
| Adlercreutzia | rs9915817 | T | C | 0.075 | 0.017 | 8.55E-06 | 0.092 | 0.058 | 0.114 |
| Adlercreutzia | rs55719207 | G | A | -0.070 | 0.016 | 9.66E-06 | 0.035 | 0.054 | 0.524 |
| Adlercreutzia | rs11604400 | C | T | -0.103 | 0.023 | 1.27E-05 | 0.071 | 0.086 | 0.409 |
| Akkermansia | rs11729256 | T | C | 0.075 | 0.015 | 5.82E-07 | -0.045 | 0.070 | 0.526 |
| Akkermansia | rs9349825 | A | G | -0.070 | 0.015 | 1.75E-06 | 0.002 | 0.068 | 0.980 |
| Akkermansia | rs4936098 | A | G | 0.065 | 0.014 | 1.79E-06 | 0.025 | 0.055 | 0.659 |
| Akkermansia | rs2602429 | C | T | 0.075 | 0.016 | 1.83E-06 | 0.033 | 0.060 | 0.588 |
| Akkermansia | rs74542928 | T | C | 0.113 | 0.024 | 1.90E-06 | 0.015 | 0.125 | 0.902 |
| Akkermansia | rs117107102 | A | G | 0.204 | 0.043 | 2.18E-06 | 0.047 | 0.125 | 0.711 |
| Akkermansia | rs12908520 | G | A | 0.062 | 0.013 | 2.39E-06 | 0.010 | 0.054 | 0.850 |
| Akkermansia | rs4242783 | G | A | 0.069 | 0.015 | 3.47E-06 | 0.021 | 0.059 | 0.719 |
| Akkermansia | rs111862613 | T | C | 0.091 | 0.020 | 3.63E-06 | -0.012 | 0.070 | 0.866 |
| Akkermansia | rs61779207 | G | A | -0.076 | 0.017 | 5.81E-06 | -0.008 | 0.064 | 0.898 |
| Akkermansia | rs941682 | G | A | -0.063 | 0.014 | 1.07E-05 | -0.022 | 0.059 | 0.713 |
| Alistipes | rs11769002 | G | A | -0.053 | 0.011 | 1.34E-06 | -0.109 | 0.054 | 0.042 |
| Alistipes | rs7129639 | C | A | -0.052 | 0.011 | 1.66E-06 | 0.008 | 0.057 | 0.894 |
| Alistipes | rs8130320 | A | G | -0.049 | 0.011 | 4.82E-06 | 0.033 | 0.053 | 0.535 |
| Alistipes | rs1689282 | A | C | -0.052 | 0.011 | 5.01E-06 | -0.077 | 0.057 | 0.172 |
| Alistipes | rs34417064 | A | G | -0.048 | 0.011 | 6.47E-06 | -0.014 | 0.053 | 0.797 |
| Alistipes | rs11958296 | A | G | -0.098 | 0.022 | 6.94E-06 | 0.121 | 0.131 | 0.353 |
| Alistipes | rs62576416 | T | C | 0.049 | 0.011 | 7.14E-06 | -0.032 | 0.055 | 0.557 |
| Alistipes | rs12990744 | C | T | -0.078 | 0.017 | 7.14E-06 | 0.144 | 0.089 | 0.104 |
| Alistipes | rs4810359 | A | G | -0.065 | 0.015 | 8.05E-06 | -0.059 | 0.081 | 0.460 |
| Alistipes | rs1107244 | G | A | 0.076 | 0.017 | 9.37E-06 | -0.115 | 0.099 | 0.243 |
| Alistipes | rs2875322 | T | C | -0.058 | 0.013 | 9.87E-06 | 0.042 | 0.071 | 0.556 |
| Alistipes | rs35909684 | A | C | -0.085 | 0.019 | 1.08E-05 | 0.029 | 0.102 | 0.779 |
| Alistipes | rs2290844 | C | T | 0.081 | 0.019 | 2.16E-05 | -0.017 | 0.083 | 0.840 |
| Allisonella | rs602075 | A | G | 0.169 | 0.030 | 1.27E-08 | -0.011 | 0.060 | 0.855 |
| Allisonella | rs35778461 | C | T | 0.147 | 0.030 | 7.99E-07 | 0.106 | 0.063 | 0.093 |
| Allisonella | rs6742198 | G | A | 0.149 | 0.032 | 2.44E-06 | -0.004 | 0.062 | 0.950 |
| Allisonella | rs1901739 | T | G | 0.116 | 0.025 | 3.22E-06 | -0.001 | 0.053 | 0.986 |
| Allisonella | rs35110698 | T | C | -0.146 | 0.032 | 5.11E-06 | -0.052 | 0.075 | 0.486 |
| Allisonella | rs7898615 | T | G | 0.168 | 0.037 | 6.92E-06 | 0.082 | 0.077 | 0.286 |
| Allisonella | rs594561 | C | T | 0.112 | 0.025 | 8.22E-06 | -0.042 | 0.053 | 0.430 |
| Allisonella | rs76904847 | G | A | 0.149 | 0.033 | 9.19E-06 | -0.103 | 0.071 | 0.145 |
| Alloprevotella | rs12675596 | G | T | 0.146 | 0.029 | 5.41E-07 | -0.067 | 0.065 | 0.303 |
| Alloprevotella | rs4680035 | A | G | -0.120 | 0.026 | 4.03E-06 | -0.006 | 0.054 | 0.908 |
| Alloprevotella | rs34619204 | G | A | -0.156 | 0.034 | 5.88E-06 | -0.016 | 0.070 | 0.815 |
| Alloprevotella | rs58212166 | A | G | -0.162 | 0.036 | 6.88E-06 | 0.025 | 0.068 | 0.712 |
| Alloprevotella | rs4364940 | A | G | 0.126 | 0.028 | 7.48E-06 | 0.088 | 0.057 | 0.126 |
| Alloprevotella | rs2154444 | T | G | 0.138 | 0.031 | 7.67E-06 | -0.143 | 0.060 | 0.017 |
| Anaerofilum | rs816292 | T | C | -0.113 | 0.022 | 2.94E-07 | -0.006 | 0.058 | 0.923 |
| Anaerofilum | rs79598899 | C | T | 0.183 | 0.036 | 3.22E-07 | -0.148 | 0.128 | 0.247 |
| Anaerofilum | rs712981 | A | C | 0.101 | 0.020 | 6.84E-07 | -0.016 | 0.054 | 0.767 |
| Anaerofilum | rs4506496 | G | A | 0.103 | 0.021 | 1.28E-06 | 0.055 | 0.059 | 0.346 |
| Anaerofilum | rs10794359 | T | C | -0.095 | 0.020 | 2.00E-06 | -0.036 | 0.054 | 0.508 |
| Anaerofilum | rs17096874 | C | T | -0.126 | 0.027 | 2.63E-06 | -0.037 | 0.065 | 0.575 |
| Anaerofilum | rs356049 | G | A | 0.133 | 0.029 | 4.75E-06 | -0.086 | 0.107 | 0.420 |
| Anaerofilum | rs1563175 | A | C | 0.092 | 0.020 | 4.88E-06 | -0.019 | 0.053 | 0.720 |
| Anaerofilum | rs17012738 | T | G | 0.090 | 0.020 | 6.43E-06 | 0.005 | 0.053 | 0.930 |
| Anaerofilum | rs9299345 | T | C | -0.136 | 0.030 | 6.45E-06 | 0.092 | 0.089 | 0.303 |
| Anaerofilum | rs4244069 | G | A | -0.147 | 0.033 | 6.99E-06 | 0.058 | 0.080 | 0.464 |
| Anaerostipes | rs7193624 | C | T | 0.075 | 0.015 | 6.35E-07 | -0.019 | 0.097 | 0.848 |
| Anaerostipes | rs2804244 | A | G | -0.053 | 0.011 | 1.72E-06 | -0.018 | 0.055 | 0.743 |
| Anaerostipes | rs62157625 | T | C | 0.089 | 0.019 | 1.81E-06 | -0.121 | 0.081 | 0.136 |
| Anaerostipes | rs62215703 | G | A | 0.064 | 0.014 | 2.38E-06 | 0.055 | 0.064 | 0.387 |
| Anaerostipes | rs2396460 | T | C | -0.051 | 0.011 | 2.88E-06 | 0.039 | 0.054 | 0.465 |
| Anaerostipes | rs6854026 | T | C | -0.051 | 0.011 | 3.14E-06 | -0.080 | 0.053 | 0.135 |
| Anaerostipes | rs3900776 | G | A | -0.110 | 0.024 | 3.23E-06 | 0.409 | 0.160 | 0.011 |
| Anaerostipes | rs6726833 | C | A | -0.088 | 0.019 | 3.61E-06 | 0.076 | 0.099 | 0.445 |
| Anaerostipes | rs60983350 | G | A | -0.054 | 0.012 | 3.63E-06 | -0.028 | 0.057 | 0.628 |
| Anaerostipes | rs2014785 | T | C | 0.052 | 0.011 | 4.30E-06 | -0.071 | 0.054 | 0.190 |
| Anaerostipes | rs78735375 | A | C | -0.137 | 0.031 | 6.75E-06 | 0.065 | 0.136 | 0.629 |
| Anaerostipes | rs6474958 | A | G | -0.050 | 0.011 | 8.01E-06 | -0.034 | 0.057 | 0.558 |
| Anaerostipes | rs10502061 | A | G | 0.084 | 0.019 | 1.35E-05 | 0.075 | 0.083 | 0.367 |
| Anaerotruncus | rs6563550 | T | C | 0.088 | 0.018 | 6.95E-07 | -0.115 | 0.099 | 0.243 |
| Anaerotruncus | rs1272208 | G | T | -0.061 | 0.013 | 2.45E-06 | -0.010 | 0.063 | 0.869 |
| Anaerotruncus | rs8005030 | C | T | 0.055 | 0.012 | 2.54E-06 | 0.087 | 0.056 | 0.123 |
| Anaerotruncus | rs4669806 | G | T | 0.058 | 0.012 | 2.78E-06 | -0.061 | 0.064 | 0.346 |
| Anaerotruncus | rs9347879 | T | C | 0.051 | 0.011 | 4.62E-06 | 0.012 | 0.053 | 0.819 |
| Anaerotruncus | rs115414803 | A | C | -0.144 | 0.032 | 5.46E-06 | -0.001 | 0.111 | 0.995 |
| Anaerotruncus | rs10150232 | A | G | 0.057 | 0.012 | 5.59E-06 | -0.005 | 0.066 | 0.938 |
| Anaerotruncus | rs7155595 | C | A | 0.054 | 0.012 | 5.73E-06 | 0.028 | 0.058 | 0.626 |
| Anaerotruncus | rs1431492 | C | T | -0.065 | 0.015 | 7.45E-06 | -0.080 | 0.073 | 0.271 |
| Anaerotruncus | rs6494922 | A | G | 0.090 | 0.020 | 8.00E-06 | 0.047 | 0.119 | 0.692 |
| Anaerotruncus | rs17734739 | T | C | 0.066 | 0.015 | 9.53E-06 | -0.007 | 0.076 | 0.922 |
| Anaerotruncus | rs34449434 | A | C | -0.050 | 0.011 | 1.17E-05 | -0.036 | 0.056 | 0.516 |
| Anaerotruncus | rs11018566 | A | G | -0.156 | 0.037 | 1.91E-05 | 0.052 | 0.116 | 0.655 |
| Bacteroides | rs6795673 | C | T | 0.054 | 0.011 | 3.11E-07 | -0.031 | 0.053 | 0.560 |
| Bacteroides | rs11585893 | A | G | -0.074 | 0.015 | 5.24E-07 | 0.086 | 0.062 | 0.168 |
| Bacteroides | rs66474973 | G | T | 0.081 | 0.016 | 7.81E-07 | -0.049 | 0.087 | 0.571 |
| Bacteroides | rs17619981 | T | G | 0.088 | 0.019 | 2.46E-06 | 0.131 | 0.078 | 0.091 |
| Bacteroides | rs9507307 | C | T | 0.060 | 0.013 | 2.85E-06 | -0.021 | 0.062 | 0.733 |
| Bacteroides | rs2023437 | T | C | -0.078 | 0.017 | 3.06E-06 | 0.056 | 0.081 | 0.491 |
| Bacteroides | rs66710942 | C | T | 0.049 | 0.011 | 5.53E-06 | -0.051 | 0.053 | 0.334 |
| Bacteroides | rs13207588 | A | G | -0.059 | 0.013 | 6.40E-06 | -0.175 | 0.068 | 0.010 |
| Bacteroides | rs1340391 | T | C | -0.059 | 0.013 | 7.58E-06 | -0.011 | 0.078 | 0.888 |
| Barnesiella | rs2276875 | A | G | -0.070 | 0.014 | 5.84E-07 | -0.077 | 0.061 | 0.208 |
| Barnesiella | rs2428166 | G | A | -0.166 | 0.034 | 8.78E-07 | -0.179 | 0.226 | 0.428 |
| Barnesiella | rs35177866 | A | G | 0.092 | 0.019 | 1.41E-06 | -0.124 | 0.103 | 0.228 |
| Barnesiella | rs13242616 | T | C | -0.058 | 0.012 | 2.15E-06 | -0.072 | 0.057 | 0.212 |
| Barnesiella | rs199035 | G | A | 0.056 | 0.012 | 2.96E-06 | -0.070 | 0.053 | 0.191 |
| Barnesiella | rs79795328 | A | G | -0.082 | 0.018 | 3.49E-06 | 0.026 | 0.076 | 0.730 |
| Barnesiella | rs62251337 | A | G | -0.069 | 0.015 | 3.76E-06 | -0.022 | 0.076 | 0.769 |
| Barnesiella | rs113258194 | A | G | 0.099 | 0.021 | 3.78E-06 | -0.007 | 0.090 | 0.934 |
| Barnesiella | rs12909713 | C | T | -0.055 | 0.012 | 4.48E-06 | 0.003 | 0.053 | 0.951 |
| Barnesiella | rs77455852 | T | G | -0.089 | 0.020 | 5.17E-06 | 0.035 | 0.076 | 0.648 |
| Barnesiella | rs76181748 | C | T | -0.078 | 0.017 | 5.73E-06 | 0.005 | 0.063 | 0.933 |
| Barnesiella | rs72684847 | T | C | -0.114 | 0.025 | 6.64E-06 | -0.114 | 0.101 | 0.260 |
| Barnesiella | rs11155559 | T | C | 0.096 | 0.021 | 7.15E-06 | 0.139 | 0.091 | 0.129 |
| Bifidobacterium | rs182549 | C | T | 0.120 | 0.013 | 5.27E-21 | -0.011 | 0.054 | 0.836 |
| Bifidobacterium | rs7322849 | T | C | 0.112 | 0.020 | 2.53E-08 | -0.038 | 0.093 | 0.681 |
| Bifidobacterium | rs62181700 | G | A | -0.062 | 0.013 | 1.93E-06 | 0.028 | 0.062 | 0.652 |
| Bifidobacterium | rs56108664 | T | C | 0.073 | 0.016 | 3.80E-06 | 0.124 | 0.079 | 0.115 |
| Bifidobacterium | rs857444 | C | T | 0.056 | 0.012 | 4.12E-06 | -0.055 | 0.055 | 0.320 |
| Bifidobacterium | rs540489 | T | G | -0.064 | 0.014 | 4.31E-06 | -0.028 | 0.070 | 0.686 |
| Bifidobacterium | rs75344046 | C | T | 0.232 | 0.051 | 4.39E-06 | 0.239 | 0.124 | 0.055 |
| Bifidobacterium | rs73797465 | T | G | -0.095 | 0.021 | 5.18E-06 | 0.010 | 0.084 | 0.904 |
| Bifidobacterium | rs55888705 | A | G | 0.055 | 0.012 | 6.49E-06 | -0.033 | 0.059 | 0.574 |
| Bifidobacterium | rs2686790 | T | C | 0.071 | 0.016 | 7.49E-06 | 0.055 | 0.075 | 0.459 |
| Bifidobacterium | rs2491158 | G | A | 0.071 | 0.016 | 8.25E-06 | 0.002 | 0.079 | 0.982 |
| Bifidobacterium | rs12022129 | G | A | 0.062 | 0.014 | 8.28E-06 | -0.055 | 0.059 | 0.353 |
| Bifidobacterium | rs5746486 | T | C | -0.054 | 0.012 | 9.04E-06 | 0.035 | 0.054 | 0.522 |
| Bilophila | rs7802841 | C | A | 0.067 | 0.014 | 1.14E-06 | 0.064 | 0.058 | 0.269 |
| Bilophila | rs1571225 | C | T | 0.083 | 0.017 | 1.26E-06 | 0.031 | 0.072 | 0.663 |
| Bilophila | rs10758666 | T | G | 0.082 | 0.017 | 1.83E-06 | 0.052 | 0.071 | 0.470 |
| Bilophila | rs3827020 | C | T | 0.077 | 0.016 | 1.83E-06 | -0.009 | 0.067 | 0.899 |
| Bilophila | rs1327489 | C | T | 0.081 | 0.017 | 2.07E-06 | 0.036 | 0.072 | 0.615 |
| Bilophila | rs6793291 | C | A | 0.113 | 0.024 | 3.08E-06 | -0.013 | 0.120 | 0.911 |
| Bilophila | rs4506138 | T | C | 0.064 | 0.014 | 3.19E-06 | 0.049 | 0.057 | 0.389 |
| Bilophila | rs1241171 | G | A | -0.069 | 0.015 | 3.97E-06 | -0.035 | 0.075 | 0.640 |
| Bilophila | rs542415 | T | C | -0.061 | 0.013 | 4.25E-06 | 0.062 | 0.054 | 0.253 |
| Bilophila | rs45577639 | A | G | 0.069 | 0.015 | 4.27E-06 | 0.032 | 0.067 | 0.639 |
| Bilophila | rs10954637 | G | T | 0.063 | 0.014 | 4.57E-06 | 0.053 | 0.057 | 0.357 |
| Bilophila | rs72676854 | T | C | 0.123 | 0.027 | 4.58E-06 | -0.012 | 0.119 | 0.922 |
| Bilophila | rs10230993 | C | T | 0.063 | 0.014 | 4.99E-06 | 0.053 | 0.057 | 0.353 |
| Bilophila | rs1263061 | A | G | -0.072 | 0.016 | 5.59E-06 | -0.028 | 0.079 | 0.729 |
| Bilophila | rs1676510 | A | G | -0.071 | 0.016 | 6.74E-06 | -0.028 | 0.079 | 0.729 |
| Bilophila | rs2728491 | G | T | -0.063 | 0.014 | 6.83E-06 | -0.070 | 0.062 | 0.258 |
| Bilophila | rs1763359 | C | T | -0.071 | 0.016 | 6.88E-06 | -0.028 | 0.079 | 0.729 |
| Bilophila | rs1241179 | A | G | -0.071 | 0.016 | 8.27E-06 | -0.028 | 0.079 | 0.726 |
| Bilophila | rs10974873 | C | T | 0.066 | 0.015 | 8.39E-06 | 0.060 | 0.060 | 0.317 |
| Bilophila | rs1261690 | T | C | -0.070 | 0.016 | 8.51E-06 | -0.028 | 0.079 | 0.726 |
| Bilophila | rs1969927 | G | A | 0.056 | 0.013 | 8.67E-06 | -0.083 | 0.056 | 0.140 |
| Bilophila | rs60178956 | G | A | -0.062 | 0.014 | 1.00E-05 | 0.052 | 0.064 | 0.412 |
| Bilophila | rs11069458 | T | C | -0.068 | 0.016 | 1.12E-05 | -0.067 | 0.069 | 0.330 |
| Bilophila | rs9899990 | A | G | -0.103 | 0.023 | 1.13E-05 | 0.071 | 0.097 | 0.464 |
| Bilophila | rs2713348 | A | G | 0.061 | 0.014 | 1.20E-05 | 0.008 | 0.058 | 0.893 |
| Bilophila | rs4798126 | G | A | 0.073 | 0.017 | 1.28E-05 | -0.038 | 0.067 | 0.566 |
| Bilophila | rs59441360 | C | A | 0.074 | 0.017 | 1.30E-05 | -0.035 | 0.067 | 0.601 |
| Bilophila | rs11081071 | C | T | 0.075 | 0.017 | 1.31E-05 | -0.034 | 0.067 | 0.612 |
| Bilophila | rs3862170 | G | A | 0.074 | 0.017 | 1.61E-05 | -0.033 | 0.067 | 0.619 |
| Blautia | rs11149971 | C | T | 0.118 | 0.023 | 4.96E-07 | -0.064 | 0.116 | 0.582 |
| Blautia | rs67794373 | C | T | 0.060 | 0.012 | 1.09E-06 | 0.055 | 0.063 | 0.388 |
| Blautia | rs12453000 | C | T | 0.063 | 0.013 | 1.50E-06 | 0.023 | 0.078 | 0.771 |
| Blautia | rs72973581 | A | G | 0.125 | 0.027 | 2.40E-06 | 0.062 | 0.113 | 0.587 |
| Blautia | rs115043014 | G | A | -0.207 | 0.044 | 2.65E-06 | 0.061 | 0.208 | 0.770 |
| Blautia | rs4926264 | T | C | 0.083 | 0.018 | 3.56E-06 | -0.073 | 0.087 | 0.399 |
| Blautia | rs682885 | A | G | -0.049 | 0.011 | 4.32E-06 | 0.001 | 0.057 | 0.987 |
| Blautia | rs7860714 | A | G | -0.050 | 0.011 | 4.85E-06 | 0.090 | 0.056 | 0.105 |
| Blautia | rs113271346 | C | T | 0.078 | 0.017 | 5.43E-06 | 0.002 | 0.095 | 0.985 |
| Blautia | rs3005511 | A | G | 0.050 | 0.011 | 6.08E-06 | -0.126 | 0.058 | 0.030 |
| Blautia | rs117001700 | T | C | 0.196 | 0.044 | 8.48E-06 | -0.130 | 0.228 | 0.569 |
| Blautia | rs16892041 | T | C | -0.062 | 0.014 | 1.13E-05 | -0.075 | 0.065 | 0.251 |
| Blautia | rs2788271 | T | G | -0.058 | 0.013 | 1.62E-05 | 0.054 | 0.069 | 0.433 |
| Butyricicoccus | rs56221232 | T | C | 0.083 | 0.017 | 7.56E-07 | -0.148 | 0.089 | 0.096 |
| Butyricicoccus | rs12585793 | T | C | -0.262 | 0.056 | 3.43E-06 | -0.026 | 0.156 | 0.869 |
| Butyricicoccus | rs2017189 | G | T | -0.051 | 0.011 | 4.25E-06 | 0.076 | 0.053 | 0.155 |
| Butyricicoccus | rs62478070 | T | G | 0.224 | 0.049 | 6.00E-06 | -0.024 | 0.168 | 0.888 |
| Butyricicoccus | rs4962426 | G | T | 0.061 | 0.014 | 6.27E-06 | -0.088 | 0.067 | 0.188 |
| Butyricicoccus | rs7322368 | T | C | 0.082 | 0.018 | 8.45E-06 | -0.123 | 0.093 | 0.185 |
| Butyricicoccus | rs10084203 | A | G | 0.055 | 0.012 | 8.64E-06 | 0.039 | 0.079 | 0.622 |
| Butyricicoccus | rs12034718 | A | G | 0.070 | 0.016 | 9.34E-06 | -0.059 | 0.064 | 0.351 |
| Butyricimonas | rs113054641 | G | A | -0.145 | 0.027 | 1.32E-07 | 0.016 | 0.127 | 0.899 |
| Butyricimonas | rs62390301 | T | C | -0.087 | 0.017 | 5.99E-07 | 0.057 | 0.066 | 0.395 |
| Butyricimonas | rs7083431 | A | C | 0.070 | 0.014 | 1.11E-06 | 0.052 | 0.060 | 0.380 |
| Butyricimonas | rs62130338 | G | A | -0.073 | 0.016 | 3.71E-06 | 0.040 | 0.056 | 0.477 |
| Butyricimonas | rs71428626 | G | T | -0.133 | 0.029 | 4.34E-06 | 0.060 | 0.150 | 0.691 |
| Butyricimonas | rs9657374 | C | T | 0.068 | 0.015 | 4.35E-06 | -0.009 | 0.058 | 0.880 |
| Butyricimonas | rs78453362 | A | G | -0.149 | 0.033 | 4.87E-06 | -0.368 | 0.170 | 0.030 |
| Butyricimonas | rs1862649 | G | A | 0.113 | 0.025 | 5.16E-06 | 0.004 | 0.105 | 0.966 |
| Butyricimonas | rs11228830 | A | G | 0.135 | 0.030 | 5.82E-06 | -0.003 | 0.096 | 0.974 |
| Butyricimonas | rs12458763 | A | C | 0.122 | 0.027 | 5.99E-06 | 0.124 | 0.129 | 0.335 |
| Butyricimonas | rs2114713 | G | T | 0.063 | 0.014 | 6.41E-06 | 0.094 | 0.053 | 0.079 |
| Butyricimonas | rs72814525 | A | G | 0.066 | 0.015 | 9.22E-06 | 0.109 | 0.062 | 0.081 |
| Butyricimonas | rs12304031 | G | A | -0.086 | 0.020 | 1.17E-05 | 0.043 | 0.081 | 0.594 |
| Butyrivibrio | rs72723662 | C | T | 0.224 | 0.045 | 6.14E-07 | -0.120 | 0.078 | 0.123 |
| Butyrivibrio | rs7752361 | A | G | -0.119 | 0.024 | 6.75E-07 | -0.098 | 0.053 | 0.064 |
| Butyrivibrio | rs11761679 | T | C | 0.155 | 0.032 | 1.48E-06 | -0.023 | 0.077 | 0.765 |
| Butyrivibrio | rs4537857 | T | C | -0.125 | 0.026 | 1.81E-06 | 0.009 | 0.056 | 0.873 |
| Butyrivibrio | rs16941336 | C | T | 0.127 | 0.027 | 1.95E-06 | -0.053 | 0.063 | 0.405 |
| Butyrivibrio | rs7763512 | G | A | 0.120 | 0.025 | 2.24E-06 | -0.024 | 0.054 | 0.652 |
| Butyrivibrio | rs74622183 | A | G | -0.201 | 0.043 | 2.67E-06 | -0.123 | 0.093 | 0.187 |
| Butyrivibrio | rs17163238 | G | A | 0.141 | 0.031 | 5.09E-06 | -0.061 | 0.067 | 0.364 |
| Butyrivibrio | rs9349693 | A | G | 0.118 | 0.026 | 5.63E-06 | 0.013 | 0.058 | 0.823 |
| Butyrivibrio | rs1007475 | G | T | 0.118 | 0.026 | 6.22E-06 | 0.041 | 0.059 | 0.488 |
| Butyrivibrio | rs486484 | A | G | -0.108 | 0.024 | 6.35E-06 | -0.007 | 0.054 | 0.898 |
| Butyrivibrio | rs28846706 | A | G | 0.176 | 0.039 | 7.13E-06 | 0.052 | 0.088 | 0.554 |
| Butyrivibrio | rs4928024 | A | G | -0.175 | 0.039 | 7.21E-06 | 0 | 0.068 | 1.000 |
| Butyrivibrio | rs142855850 | A | G | 0.205 | 0.046 | 7.30E-06 | -0.006 | 0.090 | 0.947 |
| Butyrivibrio | rs16934069 | T | C | -0.134 | 0.030 | 7.90E-06 | 0.051 | 0.071 | 0.472 |
| CandidatusSoleaferrea | rs61825792 | T | C | 0.112 | 0.023 | 1.30E-06 | -0.034 | 0.073 | 0.638 |
| CandidatusSoleaferrea | rs10108780 | A | G | -0.093 | 0.020 | 3.38E-06 | -0.118 | 0.062 | 0.057 |
| CandidatusSoleaferrea | rs10090365 | A | G | -0.083 | 0.018 | 4.02E-06 | 0.012 | 0.053 | 0.828 |
| CandidatusSoleaferrea | rs10809135 | T | C | 0.083 | 0.018 | 4.73E-06 | -0.003 | 0.054 | 0.960 |
| CandidatusSoleaferrea | rs4678258 | T | C | 0.099 | 0.022 | 4.81E-06 | 0.076 | 0.062 | 0.223 |
| CandidatusSoleaferrea | rs9973954 | A | G | 0.089 | 0.020 | 4.99E-06 | 0.017 | 0.056 | 0.758 |
| CandidatusSoleaferrea | rs6494306 | A | G | -0.097 | 0.021 | 6.05E-06 | -0.001 | 0.057 | 0.985 |
| CandidatusSoleaferrea | rs6489992 | A | G | -0.084 | 0.019 | 7.00E-06 | -0.027 | 0.055 | 0.628 |
| CandidatusSoleaferrea | rs7400877 | T | C | -0.095 | 0.021 | 7.84E-06 | 0.199 | 0.065 | 0.002 |
| CandidatusSoleaferrea | rs36160691 | G | A | 0.104 | 0.024 | 1.17E-05 | -0.031 | 0.057 | 0.592 |
| Catenibacterium | rs212393 | G | A | -0.135 | 0.029 | 2.29E-06 | -0.081 | 0.066 | 0.216 |
| Catenibacterium | rs12404911 | C | T | 0.141 | 0.030 | 3.72E-06 | 0.004 | 0.069 | 0.953 |
| Catenibacterium | rs73128290 | A | G | 0.130 | 0.028 | 5.14E-06 | -0.104 | 0.058 | 0.072 |
| Catenibacterium | rs7742829 | C | T | 0.114 | 0.025 | 5.51E-06 | -0.059 | 0.054 | 0.273 |
| ChristensenellaceaeR.7group | rs892686 | A | G | 0.051 | 0.011 | 3.90E-06 | 0.006 | 0.053 | 0.910 |
| ChristensenellaceaeR.7group | rs62132810 | A | G | -0.083 | 0.018 | 3.94E-06 | 0.110 | 0.076 | 0.149 |
| ChristensenellaceaeR.7group | rs78521377 | C | T | 0.125 | 0.027 | 5.40E-06 | -0.059 | 0.155 | 0.703 |
| ChristensenellaceaeR.7group | rs62467127 | C | T | 0.114 | 0.025 | 5.94E-06 | 0.220 | 0.170 | 0.196 |
| ChristensenellaceaeR.7group | rs10461257 | A | G | -0.055 | 0.012 | 6.13E-06 | -0.106 | 0.057 | 0.060 |
| ChristensenellaceaeR.7group | rs60954665 | T | G | 0.050 | 0.011 | 6.91E-06 | -0.021 | 0.053 | 0.697 |
| ChristensenellaceaeR.7group | rs79150079 | C | A | 0.122 | 0.027 | 7.27E-06 | -0.013 | 0.106 | 0.901 |
| ChristensenellaceaeR.7group | rs62190261 | A | C | 0.096 | 0.021 | 8.07E-06 | 0.018 | 0.094 | 0.850 |
| ChristensenellaceaeR.7group | rs73952017 | C | T | -0.086 | 0.019 | 9.16E-06 | 0.001 | 0.087 | 0.992 |
| ChristensenellaceaeR.7group | rs17081797 | A | G | -0.090 | 0.020 | 9.53E-06 | -0.115 | 0.108 | 0.289 |
| Clostridiuminnocuumgroup | rs6890185 | T | C | 0.113 | 0.023 | 1.14E-06 | 0.036 | 0.056 | 0.527 |
| Clostridiuminnocuumgroup | rs1942371 | G | A | -0.158 | 0.034 | 3.84E-06 | -0.069 | 0.081 | 0.396 |
| Clostridiuminnocuumgroup | rs40656 | C | T | 0.143 | 0.031 | 4.50E-06 | -0.042 | 0.066 | 0.530 |
| Clostridiuminnocuumgroup | rs61267978 | T | C | 0.147 | 0.032 | 4.57E-06 | -0.024 | 0.081 | 0.771 |
| Clostridiuminnocuumgroup | rs10074000 | T | C | -0.103 | 0.023 | 6.43E-06 | 0.021 | 0.055 | 0.697 |
| Clostridiuminnocuumgroup | rs10506058 | A | G | 0.100 | 0.022 | 7.03E-06 | -0.069 | 0.054 | 0.197 |
| Clostridiuminnocuumgroup | rs77845139 | A | G | -0.115 | 0.026 | 7.78E-06 | -0.129 | 0.062 | 0.037 |
| Clostridiuminnocuumgroup | rs6577484 | G | A | 0.160 | 0.036 | 8.76E-06 | 0.087 | 0.089 | 0.329 |
| Clostridiuminnocuumgroup | rs4869133 | G | A | -0.181 | 0.041 | 1.03E-05 | 0.063 | 0.070 | 0.369 |
| Clostridiumsensustricto1 | rs2795528 | G | A | -0.184 | 0.039 | 2.61E-06 | -0.106 | 0.115 | 0.355 |
| Clostridiumsensustricto1 | rs2817172 | C | T | 0.058 | 0.012 | 3.01E-06 | 0.050 | 0.054 | 0.353 |
| Clostridiumsensustricto1 | rs550843 | T | C | -0.078 | 0.017 | 3.68E-06 | 0.029 | 0.059 | 0.626 |
| Clostridiumsensustricto1 | rs115807074 | A | G | -0.227 | 0.049 | 3.92E-06 | 0.185 | 0.264 | 0.485 |
| Clostridiumsensustricto1 | rs12341505 | G | A | 0.081 | 0.018 | 6.76E-06 | -0.069 | 0.091 | 0.449 |
| Clostridiumsensustricto1 | rs116847295 | C | T | 0.110 | 0.025 | 7.75E-06 | 0.078 | 0.080 | 0.330 |
| Clostridiumsensustricto1 | rs11264403 | G | A | -0.139 | 0.033 | 3.21E-05 | 0.217 | 0.100 | 0.030 |
| Collinsella | rs9541268 | C | A | 0.096 | 0.020 | 1.15E-06 | -0.031 | 0.093 | 0.739 |
| Collinsella | rs2103510 | G | A | 0.079 | 0.017 | 2.93E-06 | 0.105 | 0.083 | 0.205 |
| Collinsella | rs73052258 | G | A | 0.093 | 0.020 | 4.43E-06 | 0.092 | 0.099 | 0.355 |
| Collinsella | rs62102630 | A | G | -0.070 | 0.015 | 5.59E-06 | -0.024 | 0.079 | 0.758 |
| Collinsella | rs75672793 | A | G | -0.109 | 0.024 | 5.95E-06 | 0.016 | 0.127 | 0.898 |
| Collinsella | rs10890671 | T | C | -0.054 | 0.012 | 6.13E-06 | -0.030 | 0.053 | 0.581 |
| Collinsella | rs11597285 | G | T | -0.054 | 0.012 | 8.10E-06 | -0.071 | 0.054 | 0.192 |
| Collinsella | rs149807560 | C | A | -0.104 | 0.024 | 9.83E-06 | 0.095 | 0.104 | 0.364 |
| Coprobacter | rs305411 | A | G | 0.129 | 0.026 | 1.03E-06 | 0.003 | 0.089 | 0.976 |
| Coprobacter | rs213863 | C | T | -0.089 | 0.019 | 2.47E-06 | -0.012 | 0.055 | 0.831 |
| Coprobacter | rs143662916 | C | T | 0.253 | 0.054 | 2.75E-06 | -0.135 | 0.157 | 0.390 |
| Coprobacter | rs3828477 | G | T | -0.091 | 0.020 | 3.14E-06 | -0.040 | 0.056 | 0.479 |
| Coprobacter | rs72821405 | T | C | -0.147 | 0.032 | 4.12E-06 | 0.233 | 0.094 | 0.013 |
| Coprobacter | rs11532348 | C | T | -0.104 | 0.023 | 4.61E-06 | -0.107 | 0.075 | 0.151 |
| Coprobacter | rs12684609 | T | C | 0.101 | 0.022 | 4.75E-06 | 0.095 | 0.067 | 0.156 |
| Coprobacter | rs74919520 | G | A | 0.126 | 0.028 | 5.38E-06 | 0.012 | 0.086 | 0.890 |
| Coprobacter | rs189356 | G | A | 0.078 | 0.017 | 5.53E-06 | -0.012 | 0.054 | 0.825 |
| Coprobacter | rs12908296 | A | G | 0.101 | 0.022 | 6.58E-06 | -0.115 | 0.070 | 0.099 |
| Coprobacter | rs12996055 | A | C | 0.092 | 0.021 | 1.07E-05 | 0.009 | 0.060 | 0.887 |
| Coprococcus1 | rs4277593 | G | A | -0.059 | 0.011 | 9.92E-08 | -0.016 | 0.054 | 0.773 |
| Coprococcus1 | rs74101919 | T | C | -0.072 | 0.014 | 6.68E-07 | 0.116 | 0.086 | 0.175 |
| Coprococcus1 | rs56405618 | A | G | -0.090 | 0.019 | 1.54E-06 | 0.086 | 0.083 | 0.299 |
| Coprococcus1 | rs1010560 | C | A | 0.058 | 0.012 | 2.27E-06 | 0.012 | 0.060 | 0.836 |
| Coprococcus1 | rs73031725 | T | C | 0.168 | 0.036 | 2.38E-06 | 0.072 | 0.152 | 0.637 |
| Coprococcus1 | rs1576241 | A | G | -0.051 | 0.011 | 3.17E-06 | 0.047 | 0.055 | 0.393 |
| Coprococcus1 | rs12794898 | G | T | 0.090 | 0.020 | 4.64E-06 | -0.034 | 0.080 | 0.671 |
| Coprococcus1 | rs73167075 | T | C | 0.057 | 0.013 | 7.04E-06 | 0.092 | 0.065 | 0.156 |
| Coprococcus1 | rs1762123 | C | T | -0.089 | 0.020 | 7.12E-06 | -0.072 | 0.087 | 0.410 |
| Coprococcus1 | rs946513 | C | T | 0.206 | 0.046 | 7.61E-06 | -0.103 | 0.129 | 0.424 |
| Coprococcus1 | rs2907920 | A | G | 0.056 | 0.013 | 9.65E-06 | -0.048 | 0.059 | 0.410 |
| Coprococcus1 | rs1519491 | T | C | 0.050 | 0.011 | 1.10E-05 | 0.012 | 0.055 | 0.822 |
| Coprococcus2 | rs6677933 | C | T | -0.080 | 0.016 | 9.66E-07 | -0.046 | 0.075 | 0.540 |
| Coprococcus2 | rs72680320 | T | C | -0.065 | 0.014 | 3.08E-06 | -0.098 | 0.057 | 0.083 |
| Coprococcus2 | rs2482516 | C | T | 0.075 | 0.016 | 4.59E-06 | 0.008 | 0.064 | 0.897 |
| Coprococcus2 | rs35890118 | A | G | -0.067 | 0.015 | 6.61E-06 | -0.067 | 0.061 | 0.273 |
| Coprococcus2 | rs9426473 | A | G | 0.073 | 0.016 | 6.82E-06 | 0.053 | 0.060 | 0.382 |
| Coprococcus2 | rs6894272 | T | C | -0.113 | 0.025 | 7.31E-06 | -0.057 | 0.081 | 0.485 |
| Coprococcus2 | rs12634070 | T | C | 0.074 | 0.016 | 7.98E-06 | 0.045 | 0.061 | 0.465 |
| Coprococcus2 | rs61823518 | A | C | -0.096 | 0.022 | 9.48E-06 | -0.192 | 0.086 | 0.026 |
| Coprococcus3 | rs8100692 | T | C | 0.058 | 0.011 | 3.61E-07 | -0.014 | 0.053 | 0.790 |
| Coprococcus3 | rs178271 | T | C | 0.145 | 0.029 | 7.95E-07 | -0.442 | 0.210 | 0.035 |
| Coprococcus3 | rs13394391 | C | T | -0.071 | 0.015 | 2.60E-06 | -0.035 | 0.073 | 0.636 |
| Coprococcus3 | rs7521171 | G | A | -0.060 | 0.013 | 3.96E-06 | -0.010 | 0.058 | 0.861 |
| Coprococcus3 | rs11080344 | C | T | 0.052 | 0.011 | 4.82E-06 | -0.061 | 0.053 | 0.251 |
| Coprococcus3 | rs13247359 | G | A | 0.051 | 0.011 | 5.88E-06 | -0.043 | 0.053 | 0.420 |
| Coprococcus3 | rs4575475 | G | A | 0.062 | 0.014 | 6.92E-06 | -0.043 | 0.063 | 0.499 |
| Coprococcus3 | rs10810043 | A | G | 0.052 | 0.012 | 8.70E-06 | 0.023 | 0.056 | 0.685 |
| Coprococcus3 | rs11077359 | T | C | -0.065 | 0.015 | 1.45E-05 | 0.015 | 0.071 | 0.838 |
| DefluviitaleaceaeUCG011 | rs12500663 | T | G | -0.115 | 0.023 | 4.72E-07 | 0.099 | 0.104 | 0.340 |
| DefluviitaleaceaeUCG011 | rs4677103 | A | G | 0.098 | 0.020 | 7.14E-07 | 0.061 | 0.070 | 0.388 |
| DefluviitaleaceaeUCG011 | rs112893842 | T | C | 0.114 | 0.023 | 1.02E-06 | 0.055 | 0.093 | 0.552 |
| DefluviitaleaceaeUCG011 | rs55658617 | T | C | 0.174 | 0.036 | 1.48E-06 | -0.063 | 0.146 | 0.667 |
| DefluviitaleaceaeUCG011 | rs1582238 | T | C | 0.081 | 0.017 | 1.49E-06 | 0.015 | 0.055 | 0.784 |
| DefluviitaleaceaeUCG011 | rs9608282 | T | G | 0.143 | 0.030 | 1.86E-06 | 0.071 | 0.145 | 0.628 |
| DefluviitaleaceaeUCG011 | rs9725395 | A | G | -0.138 | 0.030 | 2.86E-06 | -0.019 | 0.084 | 0.817 |
| DefluviitaleaceaeUCG011 | rs4344384 | G | T | 0.072 | 0.016 | 4.64E-06 | -0.061 | 0.053 | 0.251 |
| DefluviitaleaceaeUCG011 | rs2892880 | G | A | 0.082 | 0.018 | 6.77E-06 | 0.055 | 0.061 | 0.367 |
| Desulfovibrio | rs16863365 | A | G | 0.109 | 0.023 | 1.43E-06 | -0.017 | 0.126 | 0.892 |
| Desulfovibrio | rs13066142 | G | A | 0.119 | 0.025 | 2.05E-06 | 0.017 | 0.093 | 0.855 |
| Desulfovibrio | rs2853179 | C | T | 0.081 | 0.017 | 3.16E-06 | 0.098 | 0.064 | 0.123 |
| Desulfovibrio | rs2590913 | G | A | 0.154 | 0.034 | 5.31E-06 | -0.223 | 0.121 | 0.066 |
| Desulfovibrio | rs6580353 | T | C | 0.077 | 0.017 | 5.57E-06 | -0.033 | 0.067 | 0.620 |
| Desulfovibrio | rs4797774 | G | A | 0.213 | 0.047 | 6.11E-06 | 0.043 | 0.132 | 0.747 |
| Desulfovibrio | rs12031543 | T | C | -0.127 | 0.028 | 6.42E-06 | -0.035 | 0.077 | 0.655 |
| Desulfovibrio | rs7729080 | C | A | -0.070 | 0.016 | 8.30E-06 | -0.054 | 0.058 | 0.355 |
| Desulfovibrio | rs72647089 | T | G | -0.107 | 0.024 | 8.44E-06 | -0.062 | 0.097 | 0.526 |
| Desulfovibrio | rs2032031 | A | G | -0.065 | 0.015 | 1.06E-05 | 0.025 | 0.053 | 0.639 |
| Dialister | rs11166701 | G | A | -0.066 | 0.013 | 6.71E-07 | -0.065 | 0.053 | 0.223 |
| Dialister | rs4753063 | G | A | -0.060 | 0.013 | 4.54E-06 | -0.091 | 0.053 | 0.088 |
| Dialister | rs11071887 | T | C | 0.066 | 0.015 | 5.98E-06 | -0.002 | 0.057 | 0.968 |
| Dialister | rs4747450 | C | A | 0.067 | 0.015 | 5.99E-06 | 0.047 | 0.064 | 0.464 |
| Dialister | rs10938938 | G | A | -0.077 | 0.017 | 6.02E-06 | 0.045 | 0.073 | 0.538 |
| Dialister | rs2435610 | A | C | 0.065 | 0.014 | 6.32E-06 | -0.037 | 0.061 | 0.546 |
| Dialister | rs2314294 | T | C | 0.087 | 0.019 | 7.82E-06 | -0.034 | 0.077 | 0.660 |
| Dialister | rs764177 | C | A | -0.060 | 0.014 | 8.86E-06 | 0.129 | 0.055 | 0.019 |
| Dialister | rs76680460 | G | A | -0.161 | 0.036 | 9.55E-06 | -0.072 | 0.132 | 0.586 |
| Dialister | rs75416973 | A | G | 0.073 | 0.016 | 9.85E-06 | 0.044 | 0.064 | 0.492 |
| Dialister | rs10138457 | T | C | -0.113 | 0.026 | 1.59E-05 | -0.015 | 0.091 | 0.867 |
| Dorea | rs62503162 | A | G | -0.097 | 0.019 | 5.41E-07 | 0.212 | 0.132 | 0.109 |
| Dorea | rs13279148 | G | A | 0.072 | 0.015 | 2.13E-06 | -0.025 | 0.085 | 0.768 |
| Dorea | rs4793307 | C | T | 0.057 | 0.012 | 2.76E-06 | -0.023 | 0.062 | 0.709 |
| Dorea | rs1899291 | C | T | 0.070 | 0.015 | 3.50E-06 | -0.029 | 0.073 | 0.692 |
| Dorea | rs73729431 | C | T | -0.137 | 0.030 | 4.61E-06 | 0.096 | 0.185 | 0.603 |
| Dorea | rs3005511 | A | G | 0.052 | 0.011 | 4.80E-06 | -0.126 | 0.058 | 0.030 |
| Dorea | rs11150408 | T | G | 0.049 | 0.011 | 7.58E-06 | 0.012 | 0.053 | 0.827 |
| Dorea | rs3752849 | G | A | 0.164 | 0.037 | 7.62E-06 | 0.014 | 0.122 | 0.910 |
| Dorea | rs12537781 | T | C | -0.056 | 0.013 | 9.18E-06 | -0.022 | 0.062 | 0.723 |
| Dorea | rs345219 | T | G | -0.050 | 0.011 | 1.00E-05 | -0.022 | 0.054 | 0.689 |
| Eggerthella | rs2240838 | A | G | 0.098 | 0.020 | 7.01E-07 | 0.132 | 0.054 | 0.014 |
| Eggerthella | rs112205261 | T | C | -0.189 | 0.040 | 2.96E-06 | -0.028 | 0.097 | 0.773 |
| Eggerthella | rs2223081 | G | A | 0.103 | 0.022 | 3.50E-06 | 0.078 | 0.059 | 0.188 |
| Eggerthella | rs76663501 | C | T | 0.175 | 0.038 | 3.62E-06 | -0.029 | 0.120 | 0.812 |
| Eggerthella | rs1784405 | T | G | 0.091 | 0.020 | 4.58E-06 | 0.057 | 0.053 | 0.282 |
| Eggerthella | rs3851328 | T | G | -0.108 | 0.024 | 5.20E-06 | -0.160 | 0.064 | 0.012 |
| Eggerthella | rs4985746 | G | A | 0.111 | 0.025 | 8.09E-06 | 0.100 | 0.089 | 0.266 |
| Eggerthella | rs6430926 | C | T | 0.088 | 0.020 | 8.09E-06 | 0.013 | 0.053 | 0.816 |
| Eggerthella | rs13070736 | A | C | -0.121 | 0.027 | 8.18E-06 | -0.047 | 0.071 | 0.511 |
| Eggerthella | rs67490567 | T | C | 0.108 | 0.025 | 9.87E-06 | 0.077 | 0.061 | 0.213 |
| Eisenbergiella | rs2683098 | C | T | 0.107 | 0.023 | 1.87E-06 | -0.057 | 0.064 | 0.379 |
| Eisenbergiella | rs3812426 | G | A | 0.106 | 0.022 | 2.05E-06 | -0.092 | 0.073 | 0.207 |
| Eisenbergiella | rs1508033 | A | C | 0.092 | 0.020 | 2.93E-06 | -0.055 | 0.058 | 0.340 |
| Eisenbergiella | rs4462860 | G | A | 0.094 | 0.020 | 3.02E-06 | -0.057 | 0.055 | 0.297 |
| Eisenbergiella | rs1553971 | T | G | 0.121 | 0.026 | 4.26E-06 | -0.054 | 0.064 | 0.401 |
| Eisenbergiella | rs13258851 | A | G | 0.137 | 0.030 | 5.77E-06 | 0.071 | 0.076 | 0.350 |
| Eisenbergiella | rs11027642 | C | T | 0.129 | 0.028 | 5.93E-06 | 0.024 | 0.075 | 0.755 |
| Eisenbergiella | rs11938607 | T | C | 0.098 | 0.022 | 6.33E-06 | 0.020 | 0.061 | 0.743 |
| Eisenbergiella | rs12257723 | A | C | -0.095 | 0.021 | 6.69E-06 | 0.031 | 0.056 | 0.583 |
| Eisenbergiella | rs11651545 | T | C | 0.107 | 0.024 | 6.85E-06 | -0.154 | 0.065 | 0.018 |
| Eisenbergiella | rs12710729 | C | A | 0.089 | 0.020 | 7.20E-06 | 0.067 | 0.057 | 0.235 |
| Enterorhabdus | rs114731706 | T | G | 0.182 | 0.038 | 1.85E-06 | -0.096 | 0.151 | 0.522 |
| Enterorhabdus | rs73331712 | T | C | 0.262 | 0.055 | 2.01E-06 | -0.065 | 0.134 | 0.630 |
| Enterorhabdus | rs3017103 | A | G | 0.098 | 0.021 | 2.69E-06 | -0.018 | 0.068 | 0.792 |
| Enterorhabdus | rs10098492 | T | C | 0.132 | 0.029 | 6.62E-06 | -0.024 | 0.114 | 0.832 |
| Enterorhabdus | rs77655283 | G | A | 0.133 | 0.030 | 8.27E-06 | 0.166 | 0.104 | 0.109 |
| Enterorhabdus | rs2051957 | C | T | 0.084 | 0.019 | 8.93E-06 | -0.050 | 0.066 | 0.454 |
| Erysipelatoclostridium | rs7221249 | A | G | 0.084 | 0.014 | 4.01E-09 | -0.067 | 0.053 | 0.205 |
| Erysipelatoclostridium | rs710230 | T | C | 0.143 | 0.028 | 3.52E-07 | -0.011 | 0.103 | 0.914 |
| Erysipelatoclostridium | rs4697572 | A | G | -0.081 | 0.016 | 6.91E-07 | 0.217 | 0.067 | 0.001 |
| Erysipelatoclostridium | rs58236560 | G | T | -0.111 | 0.023 | 2.12E-06 | 0.083 | 0.082 | 0.311 |
| Erysipelatoclostridium | rs6474512 | A | C | 0.067 | 0.014 | 2.87E-06 | -0.045 | 0.054 | 0.407 |
| Erysipelatoclostridium | rs622418 | A | G | -0.067 | 0.014 | 3.10E-06 | 0.062 | 0.053 | 0.240 |
| Erysipelatoclostridium | rs340991 | A | G | -0.074 | 0.016 | 3.16E-06 | 0.015 | 0.060 | 0.801 |
| Erysipelatoclostridium | rs17804233 | T | C | -0.066 | 0.014 | 4.28E-06 | -0.009 | 0.053 | 0.870 |
| Erysipelatoclostridium | rs45480394 | T | G | -0.069 | 0.015 | 6.02E-06 | 0.002 | 0.055 | 0.971 |
| Erysipelatoclostridium | rs9590927 | G | A | -0.065 | 0.014 | 6.72E-06 | -0.002 | 0.053 | 0.969 |
| Erysipelatoclostridium | rs1434153 | G | A | -0.068 | 0.015 | 7.11E-06 | -0.072 | 0.053 | 0.177 |
| Erysipelatoclostridium | rs61806970 | C | T | 0.143 | 0.032 | 8.79E-06 | 0.194 | 0.106 | 0.068 |
| Erysipelatoclostridium | rs2901723 | C | A | 0.064 | 0.014 | 8.80E-06 | -0.023 | 0.053 | 0.661 |
| Erysipelatoclostridium | rs16936671 | C | T | -0.097 | 0.022 | 8.88E-06 | -0.002 | 0.076 | 0.985 |
| Erysipelatoclostridium | rs3804326 | A | G | 0.141 | 0.034 | 2.60E-05 | -0.023 | 0.123 | 0.855 |
| ErysipelotrichaceaeUCG003 | rs76502207 | T | C | 0.145 | 0.029 | 5.86E-07 | 0.012 | 0.136 | 0.927 |
| ErysipelotrichaceaeUCG003 | rs28568391 | A | G | -0.058 | 0.012 | 8.76E-07 | -0.100 | 0.053 | 0.061 |
| ErysipelotrichaceaeUCG003 | rs11994308 | C | T | 0.115 | 0.024 | 1.94E-06 | 0.071 | 0.093 | 0.442 |
| ErysipelotrichaceaeUCG003 | rs59068084 | T | G | 0.056 | 0.012 | 2.63E-06 | 0.041 | 0.054 | 0.456 |
| ErysipelotrichaceaeUCG003 | rs6875357 | C | T | 0.166 | 0.035 | 2.84E-06 | 0.037 | 0.138 | 0.789 |
| ErysipelotrichaceaeUCG003 | rs62403464 | T | C | -0.073 | 0.016 | 2.94E-06 | 0.156 | 0.069 | 0.023 |
| ErysipelotrichaceaeUCG003 | rs17798136 | G | A | 0.159 | 0.035 | 4.92E-06 | 0.098 | 0.107 | 0.359 |
| ErysipelotrichaceaeUCG003 | rs75949021 | T | C | -0.170 | 0.037 | 5.80E-06 | 0.159 | 0.135 | 0.236 |
| ErysipelotrichaceaeUCG003 | rs4758231 | G | T | -0.055 | 0.012 | 6.16E-06 | 0.008 | 0.059 | 0.895 |
| ErysipelotrichaceaeUCG003 | rs8053479 | A | G | -0.084 | 0.019 | 6.98E-06 | 0.088 | 0.083 | 0.290 |
| ErysipelotrichaceaeUCG003 | rs2044550 | T | C | -0.061 | 0.014 | 7.50E-06 | -0.061 | 0.066 | 0.356 |
| ErysipelotrichaceaeUCG003 | rs11666127 | A | G | -0.072 | 0.016 | 8.32E-06 | 0 | 0.070 | 1.000 |
| ErysipelotrichaceaeUCG003 | rs12251396 | A | G | -0.071 | 0.016 | 8.87E-06 | -0.010 | 0.073 | 0.893 |
| ErysipelotrichaceaeUCG003 | rs73074432 | C | T | 0.072 | 0.016 | 1.14E-05 | -0.117 | 0.085 | 0.170 |
| ErysipelotrichaceaeUCG003 | rs74988980 | G | A | -0.133 | 0.035 | 1.34E-04 | 0.060 | 0.133 | 0.652 |
| Escherichia.Shigella | rs73208162 | A | G | -0.119 | 0.025 | 1.56E-06 | -0.026 | 0.153 | 0.866 |
| Escherichia.Shigella | rs1154904 | A | G | -0.061 | 0.013 | 2.67E-06 | -0.028 | 0.053 | 0.595 |
| Escherichia.Shigella | rs113127095 | A | G | 0.151 | 0.032 | 3.03E-06 | -0.106 | 0.134 | 0.429 |
| Escherichia.Shigella | rs592299 | T | C | -0.059 | 0.013 | 4.83E-06 | -0.074 | 0.054 | 0.166 |
| Escherichia.Shigella | rs2798105 | A | G | -0.101 | 0.022 | 5.57E-06 | -0.108 | 0.088 | 0.222 |
| Escherichia.Shigella | rs113513883 | A | G | 0.172 | 0.038 | 5.93E-06 | 0.040 | 0.150 | 0.788 |
| Escherichia.Shigella | rs4731451 | G | A | -0.061 | 0.014 | 6.41E-06 | -0.041 | 0.057 | 0.473 |
| Escherichia.Shigella | rs112767262 | T | C | 0.073 | 0.016 | 7.45E-06 | -0.046 | 0.064 | 0.467 |
| Escherichia.Shigella | rs57024273 | T | C | 0.063 | 0.014 | 7.63E-06 | 0.146 | 0.061 | 0.016 |
| Escherichia.Shigella | rs118526 | C | A | -0.059 | 0.014 | 1.24E-05 | -0.062 | 0.058 | 0.284 |
| Eubacteriumbrachygroup | rs55932844 | A | G | -0.171 | 0.036 | 2.26E-06 | 0.019 | 0.096 | 0.841 |
| Eubacteriumbrachygroup | rs62348779 | T | C | -0.201 | 0.043 | 3.25E-06 | -0.172 | 0.100 | 0.086 |
| Eubacteriumbrachygroup | rs4862235 | G | A | 0.105 | 0.023 | 3.44E-06 | 0.049 | 0.053 | 0.359 |
| Eubacteriumbrachygroup | rs2913110 | C | T | 0.105 | 0.023 | 4.58E-06 | -0.079 | 0.056 | 0.157 |
| Eubacteriumbrachygroup | rs1384962 | A | G | 0.121 | 0.027 | 5.62E-06 | 0.071 | 0.057 | 0.214 |
| Eubacteriumbrachygroup | rs6591893 | G | A | 0.108 | 0.024 | 6.69E-06 | -0.033 | 0.056 | 0.548 |
| Eubacteriumbrachygroup | rs13139592 | T | C | -0.146 | 0.033 | 8.12E-06 | -0.075 | 0.078 | 0.337 |
| Eubacteriumbrachygroup | rs12151423 | A | G | 0.101 | 0.023 | 8.29E-06 | 0.029 | 0.053 | 0.582 |
| Eubacteriumbrachygroup | rs73199919 | T | C | -0.237 | 0.053 | 8.38E-06 | -0.013 | 0.123 | 0.916 |
| Eubacteriumbrachygroup | rs720439 | A | G | -0.112 | 0.025 | 8.40E-06 | 0.009 | 0.062 | 0.890 |
| Eubacteriumcoprostanoligenesgroup | rs17159861 | C | T | 0.096 | 0.017 | 1.10E-08 | 0.105 | 0.086 | 0.221 |
| Eubacteriumcoprostanoligenesgroup | rs9648214 | T | C | -0.083 | 0.016 | 4.57E-07 | -0.136 | 0.095 | 0.153 |
| Eubacteriumcoprostanoligenesgroup | rs4076415 | T | G | 0.052 | 0.011 | 3.00E-06 | 0.039 | 0.055 | 0.482 |
| Eubacteriumcoprostanoligenesgroup | rs6762473 | C | A | 0.052 | 0.011 | 3.44E-06 | -0.032 | 0.056 | 0.568 |
| Eubacteriumcoprostanoligenesgroup | rs76898927 | G | A | 0.123 | 0.027 | 3.84E-06 | 0.053 | 0.117 | 0.647 |
| Eubacteriumcoprostanoligenesgroup | rs12906958 | C | T | -0.053 | 0.012 | 4.25E-06 | 0.037 | 0.058 | 0.525 |
| Eubacteriumcoprostanoligenesgroup | rs11808093 | T | C | 0.079 | 0.017 | 5.39E-06 | 0.053 | 0.073 | 0.471 |
| Eubacteriumcoprostanoligenesgroup | rs79895140 | T | C | -0.064 | 0.014 | 5.59E-06 | -0.062 | 0.085 | 0.467 |
| Eubacteriumcoprostanoligenesgroup | rs62024432 | C | T | -0.077 | 0.017 | 7.74E-06 | 0.021 | 0.090 | 0.814 |
| Eubacteriumcoprostanoligenesgroup | rs10444197 | A | G | -0.051 | 0.011 | 8.26E-06 | 0.014 | 0.056 | 0.809 |
| Eubacteriumcoprostanoligenesgroup | rs2644213 | G | A | 0.054 | 0.012 | 8.82E-06 | 0.041 | 0.058 | 0.481 |
| Eubacteriumcoprostanoligenesgroup | rs1020520 | T | G | -0.059 | 0.013 | 8.84E-06 | 0.037 | 0.074 | 0.613 |
| Eubacteriumcoprostanoligenesgroup | rs11052069 | T | C | 0.048 | 0.011 | 9.37E-06 | -0.005 | 0.054 | 0.926 |
| Eubacteriumcoprostanoligenesgroup | rs11720857 | C | T | 0.063 | 0.014 | 1.27E-05 | -0.015 | 0.070 | 0.836 |
| Eubacteriumeligensgroup | rs4583233 | A | C | 0.067 | 0.013 | 1.69E-07 | 0.014 | 0.059 | 0.819 |
| Eubacteriumeligensgroup | rs265534 | T | G | -0.056 | 0.012 | 2.69E-06 | 0.057 | 0.053 | 0.286 |
| Eubacteriumeligensgroup | rs6923695 | T | G | 0.103 | 0.023 | 6.82E-06 | 0.047 | 0.106 | 0.657 |
| Eubacteriumeligensgroup | rs2200429 | A | G | -0.089 | 0.020 | 7.53E-06 | -0.071 | 0.089 | 0.429 |
| Eubacteriumeligensgroup | rs56080211 | C | T | 0.123 | 0.028 | 1.34E-05 | 0.126 | 0.100 | 0.207 |
| Eubacteriumeligensgroup | rs182318 | G | A | -0.082 | 0.020 | 2.47E-05 | -0.080 | 0.098 | 0.412 |
| Eubacteriumfissicatenagroup | rs3771393 | C | T | 0.131 | 0.027 | 9.27E-07 | 0.095 | 0.067 | 0.155 |
| Eubacteriumfissicatenagroup | rs2733072 | G | A | 0.110 | 0.023 | 1.57E-06 | -0.029 | 0.053 | 0.582 |
| Eubacteriumfissicatenagroup | rs7104872 | G | A | 0.139 | 0.029 | 2.05E-06 | -0.006 | 0.086 | 0.946 |
| Eubacteriumfissicatenagroup | rs11876297 | T | C | 0.131 | 0.028 | 3.06E-06 | -0.134 | 0.060 | 0.025 |
| Eubacteriumfissicatenagroup | rs151257695 | A | G | 0.210 | 0.045 | 4.10E-06 | 0.188 | 0.104 | 0.071 |
| Eubacteriumfissicatenagroup | rs11818408 | G | A | 0.106 | 0.024 | 8.04E-06 | 0.058 | 0.054 | 0.288 |
| Eubacteriumfissicatenagroup | rs1768152 | T | C | 0.139 | 0.032 | 1.03E-05 | 0.024 | 0.087 | 0.785 |
| Eubacteriumfissicatenagroup | rs6934739 | A | G | 0.111 | 0.025 | 1.04E-05 | -0.010 | 0.056 | 0.858 |
| Eubacteriumfissicatenagroup | rs10147907 | T | G | 0.172 | 0.040 | 1.36E-05 | 0.131 | 0.100 | 0.191 |
| Eubacteriumhalliigroup | rs13116360 | T | C | 0.154 | 0.030 | 2.15E-07 | 0.117 | 0.108 | 0.280 |
| Eubacteriumhalliigroup | rs74018587 | C | T | 0.209 | 0.044 | 1.86E-06 | 0.091 | 0.133 | 0.494 |
| Eubacteriumhalliigroup | rs10798999 | C | T | 0.060 | 0.013 | 2.07E-06 | 0.060 | 0.060 | 0.315 |
| Eubacteriumhalliigroup | rs28584818 | A | G | 0.126 | 0.027 | 2.67E-06 | -0.107 | 0.099 | 0.280 |
| Eubacteriumhalliigroup | rs60254196 | A | G | -0.052 | 0.011 | 2.96E-06 | 0.022 | 0.053 | 0.683 |
| Eubacteriumhalliigroup | rs949971 | T | G | -0.054 | 0.012 | 3.28E-06 | 0.008 | 0.056 | 0.885 |
| Eubacteriumhalliigroup | rs10808115 | A | C | -0.050 | 0.011 | 4.39E-06 | 0.023 | 0.053 | 0.666 |
| Eubacteriumhalliigroup | rs10501370 | C | T | -0.116 | 0.025 | 4.69E-06 | -0.107 | 0.110 | 0.330 |
| Eubacteriumhalliigroup | rs6550770 | T | C | -0.198 | 0.044 | 7.97E-06 | -0.087 | 0.132 | 0.509 |
| Eubacteriumhalliigroup | rs78056098 | G | T | -0.051 | 0.011 | 8.18E-06 | 0.007 | 0.055 | 0.896 |
| Eubacteriumhalliigroup | rs281379 | A | G | -0.050 | 0.011 | 8.43E-06 | -0.044 | 0.054 | 0.419 |
| Eubacteriumhalliigroup | rs630939 | C | T | -0.051 | 0.011 | 8.57E-06 | 0.035 | 0.054 | 0.511 |
| Eubacteriumhalliigroup | rs117748144 | T | C | -0.127 | 0.029 | 1.04E-05 | 0.095 | 0.121 | 0.432 |
| Eubacteriumhalliigroup | rs17474256 | G | A | 0.081 | 0.018 | 1.12E-05 | -0.125 | 0.090 | 0.164 |
| Eubacteriumhalliigroup | rs17074066 | T | C | -0.081 | 0.019 | 1.70E-05 | 0.049 | 0.193 | 0.801 |
| Eubacteriumnodatumgroup | rs34297067 | A | G | -0.187 | 0.034 | 4.41E-08 | 0 | 0.075 | 1.000 |
| Eubacteriumnodatumgroup | rs77910827 | C | T | 0.202 | 0.041 | 1.07E-06 | -0.018 | 0.085 | 0.835 |
| Eubacteriumnodatumgroup | rs61841040 | G | T | 0.161 | 0.034 | 2.58E-06 | -0.138 | 0.067 | 0.040 |
| Eubacteriumnodatumgroup | rs113893692 | C | T | -0.185 | 0.040 | 4.50E-06 | 0.094 | 0.081 | 0.244 |
| Eubacteriumnodatumgroup | rs7880204 | T | C | -0.125 | 0.028 | 5.19E-06 | -0.067 | 0.061 | 0.273 |
| Eubacteriumnodatumgroup | rs7827125 | C | T | 0.122 | 0.027 | 6.56E-06 | 0.091 | 0.059 | 0.124 |
| Eubacteriumnodatumgroup | rs11006576 | A | G | -0.110 | 0.025 | 7.48E-06 | 0.056 | 0.054 | 0.300 |
| Eubacteriumnodatumgroup | rs6818880 | A | G | -0.110 | 0.025 | 7.62E-06 | 0.091 | 0.053 | 0.087 |
| Eubacteriumnodatumgroup | rs10458299 | T | C | -0.188 | 0.042 | 7.69E-06 | 0.024 | 0.101 | 0.816 |
| Eubacteriumnodatumgroup | rs9425984 | T | C | -0.130 | 0.029 | 8.40E-06 | 0.016 | 0.065 | 0.808 |
| Eubacteriumnodatumgroup | rs10263623 | C | T | 0.193 | 0.044 | 1.05E-05 | -0.093 | 0.139 | 0.501 |
| Eubacteriumoxidoreducensgroup | rs12423772 | G | T | 0.141 | 0.030 | 1.77E-06 | 0.122 | 0.076 | 0.109 |
| Eubacteriumoxidoreducensgroup | rs440215 | C | T | 0.093 | 0.020 | 1.79E-06 | -0.043 | 0.053 | 0.416 |
| Eubacteriumoxidoreducensgroup | rs2973294 | G | T | 0.092 | 0.020 | 2.29E-06 | -0.026 | 0.054 | 0.622 |
| Eubacteriumoxidoreducensgroup | rs34561138 | G | A | 0.216 | 0.046 | 2.61E-06 | -0.044 | 0.140 | 0.752 |
| Eubacteriumoxidoreducensgroup | rs12129908 | C | A | 0.089 | 0.020 | 6.67E-06 | -0.055 | 0.054 | 0.307 |
| Eubacteriumrectalegroup | rs35398954 | A | G | -0.090 | 0.017 | 2.44E-07 | 0.085 | 0.073 | 0.243 |
| Eubacteriumrectalegroup | rs314726 | T | C | 0.053 | 0.011 | 1.37E-06 | -0.074 | 0.053 | 0.163 |
| Eubacteriumrectalegroup | rs10248854 | C | A | -0.053 | 0.011 | 3.29E-06 | 0.060 | 0.054 | 0.269 |
| Eubacteriumrectalegroup | rs10797540 | A | G | 0.050 | 0.011 | 3.45E-06 | -0.041 | 0.053 | 0.440 |
| Eubacteriumrectalegroup | rs2884897 | A | G | -0.129 | 0.029 | 7.61E-06 | -0.081 | 0.142 | 0.569 |
| Eubacteriumrectalegroup | rs62547233 | A | G | 0.054 | 0.012 | 8.12E-06 | 0.017 | 0.059 | 0.777 |
| Eubacteriumrectalegroup | rs59427698 | A | G | -0.058 | 0.013 | 1.07E-05 | -0.044 | 0.068 | 0.518 |
| Eubacteriumrectalegroup | rs143694765 | T | C | 0.087 | 0.020 | 1.09E-05 | 0.053 | 0.087 | 0.547 |
| Eubacteriumruminantiumgroup | rs2116427 | A | G | 0.091 | 0.018 | 5.78E-07 | 0.022 | 0.061 | 0.723 |
| Eubacteriumruminantiumgroup | rs139749 | C | T | -0.085 | 0.017 | 8.60E-07 | 0.063 | 0.056 | 0.263 |
| Eubacteriumruminantiumgroup | rs10131724 | A | C | -0.200 | 0.041 | 1.43E-06 | -0.089 | 0.090 | 0.320 |
| Eubacteriumruminantiumgroup | rs2229917 | A | G | 0.154 | 0.032 | 2.14E-06 | 0.021 | 0.132 | 0.874 |
| Eubacteriumruminantiumgroup | rs72836424 | C | T | -0.140 | 0.030 | 3.32E-06 | 0.083 | 0.084 | 0.324 |
| Eubacteriumruminantiumgroup | rs73139629 | A | C | -0.115 | 0.025 | 3.44E-06 | -0.048 | 0.093 | 0.606 |
| Eubacteriumruminantiumgroup | rs57340348 | T | C | -0.098 | 0.021 | 3.91E-06 | 0.068 | 0.066 | 0.304 |
| Eubacteriumruminantiumgroup | rs606117 | A | G | 0.083 | 0.018 | 3.94E-06 | -0.084 | 0.060 | 0.160 |
| Eubacteriumruminantiumgroup | rs7000472 | A | G | -0.076 | 0.017 | 3.96E-06 | 0.115 | 0.054 | 0.033 |
| Eubacteriumruminantiumgroup | rs17519472 | C | T | 0.108 | 0.023 | 4.08E-06 | 0.077 | 0.077 | 0.315 |
| Eubacteriumruminantiumgroup | rs11637981 | G | T | -0.073 | 0.016 | 5.28E-06 | 0.015 | 0.053 | 0.779 |
| Eubacteriumruminantiumgroup | rs6676699 | G | T | -0.089 | 0.020 | 6.16E-06 | -0.099 | 0.057 | 0.084 |
| Eubacteriumruminantiumgroup | rs2418654 | C | T | -0.075 | 0.017 | 6.32E-06 | -0.048 | 0.054 | 0.378 |
| Eubacteriumruminantiumgroup | rs10923018 | G | A | 0.073 | 0.016 | 6.36E-06 | -0.023 | 0.054 | 0.669 |
| Eubacteriumruminantiumgroup | rs13025464 | T | C | -0.074 | 0.016 | 6.79E-06 | 0.077 | 0.054 | 0.157 |
| Eubacteriumruminantiumgroup | rs2817174 | C | T | -0.073 | 0.016 | 7.26E-06 | 0.061 | 0.054 | 0.264 |
| Eubacteriumruminantiumgroup | rs16891896 | G | A | -0.175 | 0.039 | 7.64E-06 | -0.004 | 0.095 | 0.970 |
| Eubacteriumruminantiumgroup | rs209813 | G | A | -0.103 | 0.024 | 1.20E-05 | -0.023 | 0.075 | 0.762 |
| Eubacteriumventriosumgroup | rs11617697 | A | G | -0.143 | 0.029 | 5.50E-07 | -0.066 | 0.116 | 0.570 |
| Eubacteriumventriosumgroup | rs73615400 | T | C | -0.096 | 0.019 | 7.65E-07 | -0.037 | 0.090 | 0.681 |
| Eubacteriumventriosumgroup | rs57199565 | T | C | 0.078 | 0.016 | 1.02E-06 | -0.102 | 0.070 | 0.143 |
| Eubacteriumventriosumgroup | rs12964517 | G | A | 0.059 | 0.012 | 1.97E-06 | -0.018 | 0.059 | 0.758 |
| Eubacteriumventriosumgroup | rs16884680 | G | T | -0.091 | 0.019 | 2.32E-06 | -0.173 | 0.087 | 0.046 |
| Eubacteriumventriosumgroup | rs876734 | C | T | -0.062 | 0.013 | 3.04E-06 | 0.062 | 0.059 | 0.294 |
| Eubacteriumventriosumgroup | rs3809430 | T | C | -0.055 | 0.012 | 3.68E-06 | -0.003 | 0.057 | 0.966 |
| Eubacteriumventriosumgroup | rs72783037 | C | A | 0.066 | 0.014 | 4.56E-06 | 0.082 | 0.065 | 0.208 |
| Eubacteriumventriosumgroup | rs78250280 | G | A | 0.075 | 0.016 | 5.10E-06 | -0.212 | 0.076 | 0.005 |
| Eubacteriumventriosumgroup | rs66746423 | C | T | 0.075 | 0.016 | 5.23E-06 | 0.142 | 0.074 | 0.054 |
| Eubacteriumventriosumgroup | rs35179274 | C | T | -0.063 | 0.014 | 5.36E-06 | -0.092 | 0.069 | 0.185 |
| Eubacteriumventriosumgroup | rs9316536 | T | G | -0.082 | 0.018 | 8.11E-06 | -0.027 | 0.076 | 0.718 |
| Eubacteriumventriosumgroup | rs6704822 | A | G | 0.074 | 0.017 | 9.25E-06 | -0.086 | 0.078 | 0.273 |
| Eubacteriumventriosumgroup | rs13082419 | C | T | -0.072 | 0.016 | 9.25E-06 | -0.159 | 0.085 | 0.062 |
| Eubacteriumventriosumgroup | rs78869071 | C | T | 0.098 | 0.023 | 1.34E-05 | 0.220 | 0.096 | 0.022 |
| Eubacteriumxylanophilumgroup | rs17830032 | G | A | -0.161 | 0.031 | 2.32E-07 | 0.009 | 0.097 | 0.930 |
| Eubacteriumxylanophilumgroup | rs13239072 | G | A | 0.069 | 0.014 | 1.46E-06 | -0.014 | 0.059 | 0.816 |
| Eubacteriumxylanophilumgroup | rs10917203 | A | C | 0.061 | 0.013 | 2.87E-06 | 0.048 | 0.054 | 0.373 |
| Eubacteriumxylanophilumgroup | rs1999224 | G | T | -0.095 | 0.020 | 3.11E-06 | 0.006 | 0.089 | 0.943 |
| Eubacteriumxylanophilumgroup | rs2213117 | T | G | 0.088 | 0.019 | 3.42E-06 | -0.153 | 0.073 | 0.036 |
| Eubacteriumxylanophilumgroup | rs112176119 | C | T | -0.113 | 0.025 | 3.90E-06 | -0.118 | 0.092 | 0.198 |
| Eubacteriumxylanophilumgroup | rs10140184 | A | C | 0.058 | 0.013 | 4.69E-06 | 0.069 | 0.054 | 0.200 |
| Eubacteriumxylanophilumgroup | rs2012708 | A | G | 0.057 | 0.013 | 6.28E-06 | -0.042 | 0.056 | 0.454 |
| Eubacteriumxylanophilumgroup | rs75586835 | A | G | -0.114 | 0.026 | 1.39E-05 | -0.101 | 0.104 | 0.333 |
| Faecalibacterium | rs75499067 | C | T | 0.228 | 0.047 | 1.01E-06 | 0.050 | 0.101 | 0.623 |
| Faecalibacterium | rs6910935 | A | G | 0.135 | 0.028 | 1.13E-06 | -0.138 | 0.112 | 0.217 |
| Faecalibacterium | rs1271565 | C | T | -0.058 | 0.012 | 1.46E-06 | -0.046 | 0.060 | 0.444 |
| Faecalibacterium | rs11776390 | T | C | -0.078 | 0.017 | 5.12E-06 | -0.007 | 0.106 | 0.947 |
| Faecalibacterium | rs114946999 | C | T | -0.086 | 0.019 | 5.52E-06 | -0.140 | 0.080 | 0.081 |
| Faecalibacterium | rs10927394 | G | T | -0.232 | 0.051 | 5.81E-06 | -0.147 | 0.194 | 0.449 |
| Faecalibacterium | rs79656633 | T | C | 0.146 | 0.032 | 6.53E-06 | -0.021 | 0.088 | 0.809 |
| Faecalibacterium | rs9536330 | T | C | -0.048 | 0.011 | 7.64E-06 | 0.101 | 0.053 | 0.058 |
| Faecalibacterium | rs2835874 | T | C | -0.087 | 0.020 | 1.04E-05 | -0.037 | 0.147 | 0.802 |
| Faecalibacterium | rs12753492 | A | C | 0.064 | 0.015 | 1.89E-05 | -0.039 | 0.085 | 0.643 |
| FamilyXIIIAD3011group | rs72730932 | C | A | -0.090 | 0.018 | 3.79E-07 | 0.047 | 0.092 | 0.609 |
| FamilyXIIIAD3011group | rs16840310 | A | G | -0.061 | 0.012 | 6.43E-07 | 0.009 | 0.054 | 0.874 |
| FamilyXIIIAD3011group | rs62200412 | C | T | -0.080 | 0.016 | 1.02E-06 | -0.013 | 0.061 | 0.826 |
| FamilyXIIIAD3011group | rs62029761 | A | G | 0.129 | 0.028 | 3.09E-06 | 0.032 | 0.119 | 0.786 |
| FamilyXIIIAD3011group | rs12314465 | A | G | -0.092 | 0.020 | 3.10E-06 | 0.116 | 0.096 | 0.228 |
| FamilyXIIIAD3011group | rs11126423 | C | T | 0.090 | 0.020 | 4.08E-06 | -0.019 | 0.094 | 0.843 |
| FamilyXIIIAD3011group | rs17156849 | G | A | -0.113 | 0.025 | 4.18E-06 | 0.104 | 0.112 | 0.355 |
| FamilyXIIIAD3011group | rs16940167 | C | T | 0.073 | 0.016 | 4.62E-06 | -0.059 | 0.067 | 0.382 |
| FamilyXIIIAD3011group | rs149302 | T | C | -0.065 | 0.014 | 6.55E-06 | 0.084 | 0.063 | 0.179 |
| FamilyXIIIAD3011group | rs9837139 | A | G | 0.108 | 0.024 | 7.78E-06 | -0.111 | 0.096 | 0.248 |
| FamilyXIIIAD3011group | rs11736617 | G | A | -0.076 | 0.017 | 1.02E-05 | 0.179 | 0.117 | 0.126 |
| FamilyXIIIAD3011group | rs739451 | C | T | 0.065 | 0.015 | 1.07E-05 | -0.005 | 0.065 | 0.941 |
| FamilyXIIIAD3011group | rs9276029 | A | G | -0.081 | 0.019 | 1.24E-05 | 0.013 | 0.072 | 0.858 |
| FamilyXIIIUCG001 | rs1426266 | T | C | -0.067 | 0.014 | 1.22E-06 | 0.080 | 0.060 | 0.183 |
| FamilyXIIIUCG001 | rs12049454 | T | C | -0.065 | 0.013 | 1.38E-06 | 0.041 | 0.055 | 0.449 |
| FamilyXIIIUCG001 | rs3842897 | G | A | -0.113 | 0.024 | 3.48E-06 | -0.040 | 0.093 | 0.664 |
| FamilyXIIIUCG001 | rs7119679 | G | A | -0.081 | 0.017 | 3.67E-06 | 0.089 | 0.062 | 0.151 |
| FamilyXIIIUCG001 | rs76463770 | A | G | 0.193 | 0.042 | 4.23E-06 | -0.240 | 0.158 | 0.129 |
| FamilyXIIIUCG001 | rs62414802 | C | T | -0.061 | 0.013 | 5.44E-06 | 0.002 | 0.061 | 0.979 |
| FamilyXIIIUCG001 | rs8076666 | A | G | 0.089 | 0.020 | 7.63E-06 | -0.072 | 0.081 | 0.371 |
| FamilyXIIIUCG001 | rs112362903 | A | G | -0.149 | 0.033 | 7.74E-06 | 0.024 | 0.146 | 0.869 |
| Flavonifractor | rs12030302 | A | G | -0.069 | 0.014 | 4.76E-07 | 0.049 | 0.053 | 0.355 |
| Flavonifractor | rs806808 | T | C | 0.067 | 0.014 | 1.03E-06 | -3.00E-04 | 0.054 | 0.995 |
| Flavonifractor | rs11811696 | T | C | -0.116 | 0.024 | 1.47E-06 | -0.004 | 0.096 | 0.969 |
| Flavonifractor | rs34066017 | A | G | 0.076 | 0.016 | 1.73E-06 | -0.021 | 0.066 | 0.750 |
| Flavonifractor | rs114873521 | C | T | -0.130 | 0.029 | 9.75E-06 | -0.040 | 0.103 | 0.701 |
| Fusicatenibacter | rs4378146 | A | C | -0.062 | 0.013 | 8.51E-07 | 0.074 | 0.061 | 0.224 |
| Fusicatenibacter | rs704418 | T | C | 0.074 | 0.015 | 9.96E-07 | -0.017 | 0.080 | 0.835 |
| Fusicatenibacter | rs62353480 | A | G | -0.070 | 0.015 | 1.45E-06 | -0.076 | 0.071 | 0.288 |
| Fusicatenibacter | rs2132128 | G | A | -0.077 | 0.016 | 1.48E-06 | 0.028 | 0.089 | 0.748 |
| Fusicatenibacter | rs2025938 | G | A | -0.097 | 0.021 | 2.49E-06 | -0.021 | 0.108 | 0.849 |
| Fusicatenibacter | rs8063430 | T | C | -0.104 | 0.022 | 2.84E-06 | -0.084 | 0.121 | 0.488 |
| Fusicatenibacter | rs3303 | T | C | -0.095 | 0.020 | 2.97E-06 | -0.010 | 0.109 | 0.927 |
| Fusicatenibacter | rs1864685 | A | C | -0.049 | 0.011 | 4.72E-06 | 0.028 | 0.054 | 0.609 |
| Fusicatenibacter | rs6515626 | G | A | 0.142 | 0.031 | 6.33E-06 | 0.089 | 0.106 | 0.401 |
| Fusicatenibacter | rs16866708 | G | A | -0.070 | 0.016 | 6.34E-06 | 0.091 | 0.067 | 0.178 |
| Fusicatenibacter | rs9905659 | G | A | -0.062 | 0.014 | 6.44E-06 | 0.064 | 0.068 | 0.352 |
| Fusicatenibacter | rs60254196 | A | G | -0.049 | 0.011 | 6.71E-06 | 0.022 | 0.053 | 0.683 |
| Fusicatenibacter | rs792108 | T | C | -0.051 | 0.011 | 8.00E-06 | -0.038 | 0.054 | 0.483 |
| Fusicatenibacter | rs206581 | A | G | -0.057 | 0.013 | 8.84E-06 | -0.040 | 0.065 | 0.537 |
| Fusicatenibacter | rs73103914 | A | G | -0.060 | 0.013 | 8.89E-06 | -0.011 | 0.074 | 0.885 |
| Fusicatenibacter | rs167879 | C | T | -0.066 | 0.015 | 9.27E-06 | -0.026 | 0.075 | 0.731 |
| Fusicatenibacter | rs10439674 | A | G | -0.057 | 0.013 | 1.08E-05 | 0.032 | 0.065 | 0.630 |
| Fusicatenibacter | rs8028026 | A | G | -0.079 | 0.018 | 1.14E-05 | 0.041 | 0.093 | 0.660 |
| Gordonibacter | rs7294633 | C | T | 0.129 | 0.025 | 2.63E-07 | -0.087 | 0.060 | 0.146 |
| Gordonibacter | rs72714787 | C | A | 0.181 | 0.038 | 1.51E-06 | -0.009 | 0.078 | 0.908 |
| Gordonibacter | rs322296 | G | A | 0.179 | 0.038 | 2.17E-06 | 0.091 | 0.102 | 0.371 |
| Gordonibacter | rs71545975 | A | G | -0.154 | 0.034 | 5.54E-06 | -0.126 | 0.070 | 0.074 |
| Gordonibacter | rs16955299 | G | A | -0.196 | 0.043 | 5.88E-06 | -0.202 | 0.089 | 0.024 |
| Gordonibacter | rs13412653 | A | C | 0.108 | 0.024 | 6.87E-06 | -0.014 | 0.055 | 0.795 |
| Gordonibacter | rs768830 | G | A | 0.150 | 0.033 | 6.93E-06 | 0.040 | 0.073 | 0.582 |
| Gordonibacter | rs35042269 | C | A | -0.180 | 0.040 | 7.80E-06 | 0.015 | 0.084 | 0.859 |
| Gordonibacter | rs4596722 | A | G | 0.103 | 0.023 | 8.83E-06 | 0.090 | 0.053 | 0.088 |
| Gordonibacter | rs61934597 | C | T | -0.172 | 0.039 | 9.03E-06 | 0.151 | 0.109 | 0.165 |
| Gordonibacter | rs3765837 | T | G | -0.191 | 0.043 | 1.09E-05 | -0.164 | 0.104 | 0.115 |
| Gordonibacter | rs72939513 | A | G | -0.214 | 0.049 | 1.29E-05 | 0.175 | 0.121 | 0.147 |
| Haemophilus | rs9382510 | C | T | -0.094 | 0.017 | 6.07E-08 | -0.017 | 0.060 | 0.772 |
| Haemophilus | rs78909003 | T | C | -0.246 | 0.050 | 1.03E-06 | 0.015 | 0.118 | 0.897 |
| Haemophilus | rs9328464 | T | C | 0.072 | 0.015 | 1.18E-06 | -0.026 | 0.053 | 0.624 |
| Haemophilus | rs76022354 | C | T | 0.245 | 0.051 | 1.30E-06 | 0.077 | 0.123 | 0.532 |
| Haemophilus | rs111582866 | G | A | -0.124 | 0.026 | 1.78E-06 | 0.011 | 0.095 | 0.905 |
| Haemophilus | rs35509 | G | A | 0.128 | 0.027 | 1.83E-06 | -0.122 | 0.132 | 0.356 |
| Haemophilus | rs10781340 | G | A | 0.095 | 0.020 | 3.02E-06 | 0.091 | 0.081 | 0.263 |
| Haemophilus | rs4822728 | T | C | 0.071 | 0.015 | 3.12E-06 | -0.086 | 0.053 | 0.105 |
| Haemophilus | rs9895850 | T | C | -0.193 | 0.042 | 3.66E-06 | 0.042 | 0.130 | 0.746 |
| Holdemanella | rs607782 | T | C | -0.085 | 0.017 | 7.36E-07 | 0.020 | 0.055 | 0.711 |
| Holdemanella | rs73011279 | T | C | -0.096 | 0.020 | 1.40E-06 | 0.059 | 0.063 | 0.354 |
| Holdemanella | rs4541991 | T | C | -0.093 | 0.019 | 1.85E-06 | 0.003 | 0.057 | 0.965 |
| Holdemanella | rs75764681 | T | C | -0.283 | 0.060 | 2.29E-06 | -0.182 | 0.129 | 0.157 |
| Holdemanella | rs1926302 | G | A | -0.108 | 0.023 | 3.08E-06 | 0.013 | 0.064 | 0.841 |
| Holdemanella | rs12513188 | G | A | 0.090 | 0.020 | 3.68E-06 | -0.055 | 0.060 | 0.360 |
| Holdemanella | rs34187114 | C | A | -0.105 | 0.023 | 3.77E-06 | -0.213 | 0.085 | 0.012 |
| Holdemanella | rs35228298 | G | A | 0.093 | 0.020 | 4.07E-06 | -0.087 | 0.073 | 0.231 |
| Holdemanella | rs4802175 | G | A | 0.079 | 0.017 | 5.19E-06 | 0.088 | 0.057 | 0.122 |
| Holdemanella | rs62113381 | T | C | -0.105 | 0.023 | 5.50E-06 | -0.086 | 0.079 | 0.273 |
| Holdemanella | rs17586763 | T | C | -0.227 | 0.051 | 8.37E-06 | -0.030 | 0.121 | 0.801 |
| Holdemania | rs1867876 | T | C | 0.084 | 0.016 | 2.02E-07 | -0.016 | 0.058 | 0.781 |
| Holdemania | rs9500080 | C | T | 0.093 | 0.018 | 2.21E-07 | 0.090 | 0.071 | 0.202 |
| Holdemania | rs77293403 | A | G | 0.165 | 0.034 | 1.47E-06 | 0.158 | 0.144 | 0.272 |
| Holdemania | rs116500994 | G | T | -0.138 | 0.029 | 2.75E-06 | 0.103 | 0.125 | 0.407 |
| Holdemania | rs9529719 | T | C | 0.074 | 0.016 | 3.97E-06 | -0.047 | 0.057 | 0.405 |
| Holdemania | rs113593397 | A | G | -0.129 | 0.028 | 5.03E-06 | 0.006 | 0.090 | 0.947 |
| Holdemania | rs111745969 | A | G | 0.121 | 0.027 | 5.61E-06 | 0.033 | 0.078 | 0.675 |
| Holdemania | rs73139538 | G | A | -0.149 | 0.033 | 5.69E-06 | 0.212 | 0.157 | 0.176 |
| Holdemania | rs4146507 | C | T | 0.079 | 0.018 | 7.10E-06 | -0.002 | 0.062 | 0.969 |
| Holdemania | rs80149660 | C | T | -0.233 | 0.052 | 7.20E-06 | -0.065 | 0.128 | 0.610 |
| Holdemania | rs10885477 | T | C | -0.135 | 0.030 | 7.60E-06 | 0.030 | 0.123 | 0.810 |
| Holdemania | rs967319 | T | C | 0.079 | 0.018 | 8.12E-06 | -0.057 | 0.062 | 0.360 |
| Holdemania | rs4636956 | A | G | -0.067 | 0.015 | 8.84E-06 | 0.002 | 0.054 | 0.964 |
| Holdemania | rs12701617 | A | G | -0.066 | 0.015 | 9.82E-06 | -0.106 | 0.053 | 0.044 |
| Howardella | rs1484873 | A | G | -0.228 | 0.046 | 8.79E-07 | -0.007 | 0.074 | 0.930 |
| Howardella | rs17167098 | G | A | -0.169 | 0.035 | 1.50E-06 | -0.060 | 0.077 | 0.440 |
| Howardella | rs672217 | G | A | 0.164 | 0.035 | 2.73E-06 | -0.095 | 0.066 | 0.153 |
| Howardella | rs609430 | T | G | -0.112 | 0.024 | 2.85E-06 | 0.051 | 0.055 | 0.356 |
| Howardella | rs12452946 | A | G | -0.106 | 0.023 | 3.79E-06 | -0.042 | 0.053 | 0.424 |
| Howardella | rs2154047 | C | A | -0.193 | 0.042 | 4.57E-06 | 0.061 | 0.094 | 0.515 |
| Howardella | rs36081916 | T | C | -0.181 | 0.040 | 6.89E-06 | 0.214 | 0.094 | 0.023 |
| Howardella | rs10048062 | C | T | -0.147 | 0.034 | 1.19E-05 | -0.049 | 0.096 | 0.607 |
| Howardella | rs3791893 | A | G | 0.147 | 0.034 | 1.55E-05 | 0.032 | 0.076 | 0.676 |
| Hungatella | rs34471047 | A | G | -0.163 | 0.033 | 1.15E-06 | -0.011 | 0.069 | 0.871 |
| Hungatella | rs17092615 | G | A | 0.152 | 0.034 | 6.61E-06 | 0.060 | 0.077 | 0.433 |
| Hungatella | rs72759041 | G | T | -0.126 | 0.028 | 8.00E-06 | -0.008 | 0.066 | 0.907 |
| Hungatella | rs13249325 | T | G | -0.100 | 0.023 | 9.51E-06 | -0.026 | 0.053 | 0.626 |
| Hungatella | rs10044993 | C | A | 0.140 | 0.032 | 1.05E-05 | -0.186 | 0.095 | 0.051 |
| Intestinibacter | rs10805326 | G | A | 0.078 | 0.014 | 2.86E-08 | 0.003 | 0.058 | 0.958 |
| Intestinibacter | rs4327025 | G | A | -0.081 | 0.015 | 1.53E-07 | 0.085 | 0.068 | 0.213 |
| Intestinibacter | rs478972 | T | C | -0.143 | 0.030 | 1.57E-06 | -0.035 | 0.092 | 0.706 |
| Intestinibacter | rs16938435 | T | C | -0.112 | 0.024 | 1.89E-06 | 0.107 | 0.090 | 0.234 |
| Intestinibacter | rs118030283 | G | A | -0.152 | 0.032 | 2.88E-06 | -0.169 | 0.129 | 0.189 |
| Intestinibacter | rs2702387 | A | G | 0.061 | 0.013 | 4.12E-06 | 0.010 | 0.054 | 0.860 |
| Intestinibacter | rs447950 | A | G | 0.063 | 0.014 | 4.26E-06 | -0.022 | 0.055 | 0.689 |
| Intestinibacter | rs6875660 | C | T | 0.089 | 0.019 | 4.39E-06 | -0.139 | 0.112 | 0.215 |
| Intestinibacter | rs6062862 | A | G | 0.092 | 0.020 | 6.26E-06 | 0.040 | 0.096 | 0.679 |
| Intestinibacter | rs11109097 | C | T | 0.062 | 0.014 | 6.60E-06 | 0.057 | 0.054 | 0.291 |
| Intestinibacter | rs2098844 | C | T | -0.058 | 0.013 | 7.47E-06 | 0.106 | 0.055 | 0.054 |
| Intestinibacter | rs9348442 | C | T | 0.099 | 0.022 | 7.80E-06 | -0.019 | 0.079 | 0.806 |
| Intestinibacter | rs893394 | G | A | 0.058 | 0.013 | 8.12E-06 | 0.008 | 0.054 | 0.888 |
| Intestinibacter | rs68093214 | C | T | 0.066 | 0.015 | 9.93E-06 | -0.002 | 0.062 | 0.978 |
| Intestinibacter | rs12210583 | T | G | 0.155 | 0.035 | 1.12E-05 | -0.062 | 0.143 | 0.664 |
| Intestinimonas | rs716604 | A | G | 0.082 | 0.017 | 8.29E-07 | -3.00E-04 | 0.063 | 0.996 |
| Intestinimonas | rs11258178 | A | G | 0.066 | 0.013 | 8.40E-07 | 0.001 | 0.053 | 0.982 |
| Intestinimonas | rs12226153 | A | G | -0.151 | 0.031 | 8.46E-07 | -0.069 | 0.230 | 0.763 |
| Intestinimonas | rs62240188 | G | A | 0.130 | 0.027 | 1.13E-06 | 0.060 | 0.090 | 0.509 |
| Intestinimonas | rs2930225 | G | T | 0.073 | 0.015 | 1.84E-06 | 0.008 | 0.063 | 0.898 |
| Intestinimonas | rs10262702 | T | C | 0.092 | 0.019 | 2.47E-06 | 0.060 | 0.081 | 0.454 |
| Intestinimonas | rs2731794 | C | T | 0.121 | 0.026 | 2.81E-06 | -0.086 | 0.147 | 0.558 |
| Intestinimonas | rs7170984 | T | C | -0.066 | 0.014 | 2.94E-06 | -0.042 | 0.059 | 0.481 |
| Intestinimonas | rs6934519 | C | T | 0.069 | 0.015 | 4.64E-06 | 0.016 | 0.061 | 0.796 |
| Intestinimonas | rs1859797 | G | A | 0.060 | 0.013 | 4.64E-06 | -0.073 | 0.053 | 0.169 |
| Intestinimonas | rs72982915 | C | T | 0.183 | 0.040 | 5.42E-06 | -0.044 | 0.116 | 0.708 |
| Intestinimonas | rs4784055 | T | C | -0.175 | 0.039 | 5.57E-06 | -0.154 | 0.122 | 0.205 |
| Intestinimonas | rs2276760 | A | G | -0.069 | 0.015 | 7.06E-06 | -0.132 | 0.062 | 0.034 |
| Intestinimonas | rs4113676 | A | C | -0.219 | 0.049 | 8.27E-06 | 0.127 | 0.224 | 0.570 |
| Intestinimonas | rs9823439 | T | C | -0.058 | 0.013 | 9.56E-06 | -0.008 | 0.053 | 0.880 |
| Intestinimonas | rs17067892 | C | T | 0.107 | 0.025 | 1.81E-05 | -0.093 | 0.093 | 0.317 |
| Lachnoclostridium | rs6112314 | A | C | -0.056 | 0.011 | 2.07E-07 | 0.065 | 0.056 | 0.253 |
| Lachnoclostridium | rs615997 | T | C | 0.051 | 0.011 | 1.54E-06 | 0.027 | 0.053 | 0.612 |
| Lachnoclostridium | rs62285313 | A | G | 0.086 | 0.018 | 1.94E-06 | -0.100 | 0.090 | 0.264 |
| Lachnoclostridium | rs789029 | C | T | -0.064 | 0.014 | 3.35E-06 | -0.027 | 0.076 | 0.719 |
| Lachnoclostridium | rs78068103 | A | G | 0.089 | 0.019 | 5.06E-06 | -0.068 | 0.083 | 0.412 |
| Lachnoclostridium | rs4738679 | G | A | -0.052 | 0.011 | 5.06E-06 | -0.012 | 0.054 | 0.830 |
| Lachnoclostridium | rs3821998 | C | A | -0.086 | 0.019 | 7.18E-06 | -0.005 | 0.086 | 0.951 |
| Lachnoclostridium | rs1031599 | G | T | -0.079 | 0.018 | 7.59E-06 | 0.005 | 0.110 | 0.966 |
| Lachnoclostridium | rs1997204 | T | C | -0.108 | 0.024 | 7.99E-06 | 0.075 | 0.128 | 0.558 |
| Lachnoclostridium | rs1528479 | G | A | -0.050 | 0.011 | 8.67E-06 | 0.030 | 0.055 | 0.585 |
| Lachnoclostridium | rs12566975 | T | C | -0.047 | 0.011 | 9.65E-06 | -0.008 | 0.053 | 0.881 |
| Lachnoclostridium | rs72829893 | G | T | 0.117 | 0.027 | 1.18E-05 | -0.095 | 0.087 | 0.276 |
| Lachnoclostridium | rs2385421 | A | G | 0.075 | 0.018 | 3.65E-05 | 0.045 | 0.082 | 0.585 |
| Lachnospira | rs13157098 | A | G | -0.077 | 0.016 | 7.60E-07 | -0.070 | 0.072 | 0.336 |
| Lachnospira | rs56791201 | T | C | 0.052 | 0.011 | 2.85E-06 | -0.037 | 0.055 | 0.500 |
| Lachnospira | rs4686798 | T | C | 0.053 | 0.011 | 2.93E-06 | -0.041 | 0.055 | 0.455 |
| Lachnospira | rs4923324 | G | A | -0.062 | 0.013 | 3.69E-06 | -0.062 | 0.072 | 0.387 |
| Lachnospira | rs2520509 | A | G | 0.052 | 0.012 | 7.35E-06 | 0.061 | 0.058 | 0.299 |
| LachnospiraceaeFCS020group | rs7249113 | G | A | 0.068 | 0.013 | 3.58E-07 | -0.028 | 0.058 | 0.633 |
| LachnospiraceaeFCS020group | rs9788306 | C | T | -0.063 | 0.013 | 1.56E-06 | 0.042 | 0.059 | 0.476 |
| LachnospiraceaeFCS020group | rs72793667 | A | G | -0.117 | 0.025 | 2.14E-06 | 0.127 | 0.136 | 0.349 |
| LachnospiraceaeFCS020group | rs2322265 | C | T | -0.067 | 0.014 | 2.52E-06 | -0.017 | 0.060 | 0.774 |
| LachnospiraceaeFCS020group | rs10093861 | G | A | -0.057 | 0.012 | 2.66E-06 | -0.008 | 0.054 | 0.880 |
| LachnospiraceaeFCS020group | rs2862811 | T | C | 0.056 | 0.012 | 3.47E-06 | -0.031 | 0.058 | 0.595 |
| LachnospiraceaeFCS020group | rs35035870 | T | C | -0.191 | 0.041 | 4.23E-06 | -0.105 | 0.137 | 0.444 |
| LachnospiraceaeFCS020group | rs1254846 | G | A | 0.106 | 0.023 | 5.18E-06 | -0.165 | 0.079 | 0.035 |
| LachnospiraceaeFCS020group | rs3999074 | G | T | -0.055 | 0.012 | 6.22E-06 | -0.091 | 0.053 | 0.085 |
| LachnospiraceaeFCS020group | rs10030408 | A | G | 0.055 | 0.012 | 7.48E-06 | -0.037 | 0.053 | 0.481 |
| LachnospiraceaeFCS020group | rs1363769 | T | C | -0.201 | 0.045 | 8.02E-06 | -0.079 | 0.153 | 0.606 |
| LachnospiraceaeFCS020group | rs4452603 | T | G | 0.060 | 0.014 | 8.84E-06 | -0.042 | 0.060 | 0.486 |
| LachnospiraceaeNC2004group | rs6116753 | G | A | 0.099 | 0.021 | 1.97E-06 | 0.005 | 0.069 | 0.947 |
| LachnospiraceaeNC2004group | rs3756315 | A | G | -0.088 | 0.019 | 2.74E-06 | 0.016 | 0.058 | 0.779 |
| LachnospiraceaeNC2004group | rs12127733 | G | A | 0.115 | 0.025 | 2.84E-06 | 0.024 | 0.069 | 0.727 |
| LachnospiraceaeNC2004group | rs34514761 | C | T | 0.114 | 0.025 | 3.51E-06 | 0.024 | 0.069 | 0.727 |
| LachnospiraceaeNC2004group | rs35598946 | G | T | 0.097 | 0.021 | 3.53E-06 | 0.007 | 0.070 | 0.919 |
| LachnospiraceaeNC2004group | rs12131508 | A | G | 0.113 | 0.025 | 4.04E-06 | 0.030 | 0.069 | 0.664 |
| LachnospiraceaeNC2004group | rs12734575 | T | C | 0.113 | 0.025 | 4.06E-06 | 0.024 | 0.069 | 0.727 |
| LachnospiraceaeNC2004group | rs12732301 | G | A | 0.112 | 0.025 | 4.84E-06 | 0.024 | 0.069 | 0.727 |
| LachnospiraceaeNC2004group | rs36062763 | T | C | 0.112 | 0.025 | 5.06E-06 | 0.024 | 0.069 | 0.727 |
| LachnospiraceaeNC2004group | rs12208226 | C | A | -0.155 | 0.034 | 5.46E-06 | 0.045 | 0.085 | 0.597 |
| LachnospiraceaeNC2004group | rs34421765 | A | G | 0.112 | 0.025 | 5.74E-06 | 0.025 | 0.069 | 0.720 |
| LachnospiraceaeNC2004group | rs1928659 | T | C | 0.103 | 0.023 | 5.97E-06 | -0.041 | 0.066 | 0.538 |
| LachnospiraceaeNC2004group | rs12863463 | G | A | -0.156 | 0.035 | 5.97E-06 | -0.035 | 0.100 | 0.723 |
| LachnospiraceaeNC2004group | rs12855868 | T | C | -0.156 | 0.035 | 6.03E-06 | -0.035 | 0.100 | 0.727 |
| LachnospiraceaeNC2004group | rs6673202 | T | G | 0.111 | 0.025 | 6.18E-06 | 0.026 | 0.069 | 0.705 |
| LachnospiraceaeNC2004group | rs12138162 | A | G | 0.111 | 0.025 | 6.35E-06 | 0.024 | 0.069 | 0.725 |
| LachnospiraceaeNC2004group | rs12137822 | T | G | 0.111 | 0.025 | 6.71E-06 | 0.024 | 0.069 | 0.723 |
| LachnospiraceaeNC2004group | rs35914870 | C | T | 0.111 | 0.025 | 6.73E-06 | 0.025 | 0.069 | 0.722 |
| LachnospiraceaeNC2004group | rs12134680 | C | A | 0.111 | 0.025 | 7.00E-06 | 0.025 | 0.069 | 0.723 |
| LachnospiraceaeNC2004group | rs147584039 | T | C | -0.154 | 0.035 | 7.87E-06 | -0.035 | 0.100 | 0.723 |
| LachnospiraceaeNC2004group | rs1929518 | C | T | 0.093 | 0.021 | 8.08E-06 | -0.108 | 0.062 | 0.083 |
| LachnospiraceaeNC2004group | rs6478407 | C | T | 0.093 | 0.021 | 8.41E-06 | -0.108 | 0.062 | 0.083 |
| LachnospiraceaeNC2004group | rs117467633 | T | C | -0.170 | 0.038 | 9.49E-06 | 0.025 | 0.138 | 0.857 |
| LachnospiraceaeNC2004group | rs1929743 | T | C | 0.084 | 0.019 | 1.09E-05 | 0.027 | 0.056 | 0.631 |
| LachnospiraceaeNC2004group | rs17067076 | G | A | -0.155 | 0.035 | 1.13E-05 | -0.058 | 0.083 | 0.484 |
| LachnospiraceaeND3007group | rs9932954 | A | G | -0.056 | 0.012 | 1.27E-06 | -0.070 | 0.056 | 0.211 |
| LachnospiraceaeND3007group | rs2861203 | G | A | 0.057 | 0.013 | 6.91E-06 | -0.118 | 0.058 | 0.043 |
| LachnospiraceaeND3007group | rs72776675 | T | C | -0.065 | 0.015 | 1.23E-05 | 0.108 | 0.070 | 0.123 |
| LachnospiraceaeNK4A136group | rs954878 | A | G | -0.052 | 0.011 | 1.81E-06 | 0.051 | 0.055 | 0.353 |
| LachnospiraceaeNK4A136group | rs7616165 | G | T | -0.231 | 0.048 | 1.86E-06 | -0.170 | 0.172 | 0.323 |
| LachnospiraceaeNK4A136group | rs68104925 | T | C | -0.055 | 0.012 | 1.95E-06 | 0.116 | 0.057 | 0.043 |
| LachnospiraceaeNK4A136group | rs160061 | A | G | 0.051 | 0.011 | 2.00E-06 | 0.051 | 0.053 | 0.339 |
| LachnospiraceaeNK4A136group | rs7832116 | A | G | -0.071 | 0.015 | 2.46E-06 | -0.006 | 0.080 | 0.945 |
| LachnospiraceaeNK4A136group | rs73044693 | A | G | -0.108 | 0.023 | 2.87E-06 | 0.165 | 0.106 | 0.119 |
| LachnospiraceaeNK4A136group | rs76193507 | A | G | -0.230 | 0.050 | 4.29E-06 | -0.029 | 0.094 | 0.756 |
| LachnospiraceaeNK4A136group | rs28540839 | A | C | 0.051 | 0.011 | 4.31E-06 | 0.014 | 0.053 | 0.795 |
| LachnospiraceaeNK4A136group | rs7073658 | T | G | -0.050 | 0.011 | 5.25E-06 | 0.073 | 0.053 | 0.171 |
| LachnospiraceaeNK4A136group | rs12611395 | A | G | -0.090 | 0.020 | 6.17E-06 | 0.165 | 0.086 | 0.056 |
| LachnospiraceaeNK4A136group | rs59805249 | T | C | 0.094 | 0.021 | 6.76E-06 | -0.068 | 0.091 | 0.458 |
| LachnospiraceaeNK4A136group | rs4955932 | T | C | -0.049 | 0.011 | 6.78E-06 | 0.044 | 0.055 | 0.421 |
| LachnospiraceaeNK4A136group | rs11263806 | A | G | -0.052 | 0.012 | 7.02E-06 | 0.017 | 0.056 | 0.765 |
| LachnospiraceaeNK4A136group | rs2880566 | T | C | 0.060 | 0.013 | 8.53E-06 | -0.178 | 0.075 | 0.018 |
| LachnospiraceaeNK4A136group | rs10952110 | G | T | 0.049 | 0.011 | 8.61E-06 | -0.064 | 0.054 | 0.233 |
| LachnospiraceaeUCG001 | rs437876 | T | C | 0.078 | 0.014 | 5.96E-08 | -0.066 | 0.056 | 0.233 |
| LachnospiraceaeUCG001 | rs985416 | C | T | 0.097 | 0.018 | 9.46E-08 | -0.062 | 0.069 | 0.369 |
| LachnospiraceaeUCG001 | rs2050911 | G | A | 0.075 | 0.015 | 1.05E-06 | 0.031 | 0.056 | 0.583 |
| LachnospiraceaeUCG001 | rs9403580 | C | T | 0.108 | 0.023 | 2.71E-06 | 0.030 | 0.079 | 0.703 |
| LachnospiraceaeUCG001 | rs573933 | T | C | -0.108 | 0.023 | 3.42E-06 | 0.034 | 0.086 | 0.693 |
| LachnospiraceaeUCG001 | rs78848836 | A | G | -0.119 | 0.026 | 4.74E-06 | -0.013 | 0.087 | 0.880 |
| LachnospiraceaeUCG001 | rs4981345 | T | C | -0.068 | 0.015 | 5.32E-06 | -0.002 | 0.056 | 0.970 |
| LachnospiraceaeUCG001 | rs12131224 | C | T | 0.117 | 0.026 | 6.20E-06 | -0.063 | 0.085 | 0.455 |
| LachnospiraceaeUCG001 | rs62496417 | T | G | -0.075 | 0.017 | 6.25E-06 | 0.071 | 0.064 | 0.267 |
| LachnospiraceaeUCG001 | rs8104225 | A | G | 0.089 | 0.020 | 6.39E-06 | 0.103 | 0.064 | 0.106 |
| LachnospiraceaeUCG001 | rs2371284 | T | C | -0.076 | 0.017 | 7.52E-06 | 0.057 | 0.063 | 0.366 |
| LachnospiraceaeUCG001 | rs8052586 | T | C | 0.176 | 0.040 | 8.42E-06 | 0.025 | 0.111 | 0.824 |
| LachnospiraceaeUCG001 | rs7341608 | T | C | -0.078 | 0.018 | 9.99E-06 | 0.063 | 0.078 | 0.420 |
| LachnospiraceaeUCG004 | rs12747809 | G | A | -0.062 | 0.013 | 7.47E-07 | -0.022 | 0.059 | 0.707 |
| LachnospiraceaeUCG004 | rs2882478 | G | A | -0.058 | 0.012 | 1.08E-06 | 0.028 | 0.053 | 0.605 |
| LachnospiraceaeUCG004 | rs12673420 | G | A | 0.055 | 0.012 | 2.83E-06 | -4.00E-04 | 0.053 | 0.994 |
| LachnospiraceaeUCG004 | rs12894272 | A | G | 0.058 | 0.013 | 3.66E-06 | 0.006 | 0.056 | 0.913 |
| LachnospiraceaeUCG004 | rs11128180 | A | G | 0.065 | 0.014 | 3.72E-06 | -0.093 | 0.062 | 0.135 |
| LachnospiraceaeUCG004 | rs2444793 | C | T | -0.054 | 0.012 | 4.44E-06 | -0.041 | 0.054 | 0.453 |
| LachnospiraceaeUCG004 | rs7629954 | A | G | 0.108 | 0.024 | 5.32E-06 | 0.046 | 0.130 | 0.726 |
| LachnospiraceaeUCG004 | rs6656451 | C | T | -0.054 | 0.012 | 5.36E-06 | 0.024 | 0.053 | 0.657 |
| LachnospiraceaeUCG004 | rs2726805 | A | G | 0.055 | 0.012 | 5.68E-06 | -0.006 | 0.054 | 0.913 |
| LachnospiraceaeUCG004 | rs35182105 | A | G | -0.110 | 0.024 | 5.89E-06 | -0.056 | 0.117 | 0.633 |
| LachnospiraceaeUCG004 | rs233486 | A | G | -0.080 | 0.018 | 6.86E-06 | -0.075 | 0.077 | 0.327 |
| LachnospiraceaeUCG004 | rs12072562 | T | C | 0.133 | 0.030 | 1.16E-05 | 0.134 | 0.138 | 0.331 |
| LachnospiraceaeUCG008 | rs10793103 | C | T | 0.097 | 0.018 | 7.67E-08 | 0.011 | 0.053 | 0.830 |
| LachnospiraceaeUCG008 | rs10741777 | T | C | -0.097 | 0.019 | 5.75E-07 | -0.007 | 0.057 | 0.906 |
| LachnospiraceaeUCG008 | rs67078837 | T | C | -0.085 | 0.017 | 7.22E-07 | -0.023 | 0.054 | 0.666 |
| LachnospiraceaeUCG008 | rs955844 | A | C | 0.112 | 0.023 | 9.24E-07 | 0.031 | 0.077 | 0.690 |
| LachnospiraceaeUCG008 | rs62277846 | C | T | 0.102 | 0.021 | 1.45E-06 | -0.097 | 0.068 | 0.151 |
| LachnospiraceaeUCG008 | rs10801803 | G | A | -0.117 | 0.024 | 1.48E-06 | -0.012 | 0.075 | 0.872 |
| LachnospiraceaeUCG008 | rs13024781 | T | C | -0.080 | 0.017 | 2.24E-06 | 0.055 | 0.053 | 0.298 |
| LachnospiraceaeUCG008 | rs57091572 | A | G | -0.110 | 0.024 | 2.82E-06 | -0.061 | 0.078 | 0.436 |
| LachnospiraceaeUCG008 | rs61944774 | A | G | 0.180 | 0.039 | 4.95E-06 | 0.027 | 0.118 | 0.820 |
| LachnospiraceaeUCG008 | rs75356640 | G | A | 0.137 | 0.030 | 6.67E-06 | 0.014 | 0.080 | 0.860 |
| LachnospiraceaeUCG008 | rs57254474 | G | A | 0.089 | 0.020 | 8.44E-06 | 0.118 | 0.065 | 0.070 |
| LachnospiraceaeUCG010 | rs11192447 | A | G | 0.127 | 0.024 | 1.99E-07 | -0.132 | 0.124 | 0.288 |
| LachnospiraceaeUCG010 | rs9981767 | A | C | 0.066 | 0.013 | 6.95E-07 | -0.003 | 0.061 | 0.964 |
| LachnospiraceaeUCG010 | rs74315802 | G | T | 0.087 | 0.018 | 2.28E-06 | -0.082 | 0.070 | 0.241 |
| LachnospiraceaeUCG010 | rs12346653 | C | T | 0.066 | 0.014 | 2.45E-06 | -0.030 | 0.065 | 0.644 |
| LachnospiraceaeUCG010 | rs72894957 | G | A | 0.222 | 0.049 | 4.89E-06 | 0.267 | 0.176 | 0.128 |
| LachnospiraceaeUCG010 | rs10414815 | T | C | 0.105 | 0.023 | 5.63E-06 | 0.024 | 0.128 | 0.852 |
| LachnospiraceaeUCG010 | rs336138 | G | T | 0.078 | 0.017 | 5.74E-06 | 0.005 | 0.083 | 0.950 |
| LachnospiraceaeUCG010 | rs4576377 | A | C | -0.057 | 0.013 | 6.72E-06 | -0.025 | 0.055 | 0.649 |
| LachnospiraceaeUCG010 | rs17730011 | G | A | -0.070 | 0.016 | 7.75E-06 | -0.098 | 0.065 | 0.129 |
| LachnospiraceaeUCG010 | rs2833528 | C | T | -0.056 | 0.013 | 1.08E-05 | -0.078 | 0.055 | 0.157 |
| Lactobacillus | rs921925 | A | C | 0.099 | 0.020 | 1.25E-06 | -0.021 | 0.065 | 0.746 |
| Lactobacillus | rs7399658 | G | A | -0.107 | 0.022 | 1.38E-06 | -0.092 | 0.069 | 0.184 |
| Lactobacillus | rs16861661 | G | A | -0.183 | 0.038 | 1.58E-06 | -0.147 | 0.107 | 0.170 |
| Lactobacillus | rs62314653 | C | A | 0.188 | 0.039 | 1.97E-06 | 0.203 | 0.113 | 0.072 |
| Lactobacillus | rs77478751 | A | G | -0.220 | 0.048 | 3.80E-06 | -0.125 | 0.083 | 0.132 |
| Lactobacillus | rs768253 | T | G | -0.079 | 0.017 | 4.03E-06 | -0.010 | 0.053 | 0.859 |
| Lactobacillus | rs12693845 | C | T | -0.081 | 0.018 | 5.64E-06 | 0.003 | 0.055 | 0.952 |
| Lactobacillus | rs1530559 | G | A | 0.080 | 0.018 | 6.43E-06 | 0.060 | 0.053 | 0.263 |
| Lactobacillus | rs75127669 | C | A | 0.140 | 0.031 | 6.70E-06 | -0.069 | 0.101 | 0.495 |
| Lactococcus | rs123059 | T | C | -0.137 | 0.027 | 6.46E-07 | -0.054 | 0.065 | 0.399 |
| Lactococcus | rs10417872 | T | G | 0.118 | 0.025 | 1.40E-06 | 0.012 | 0.059 | 0.835 |
| Lactococcus | rs4766997 | C | T | 0.115 | 0.024 | 1.53E-06 | -0.033 | 0.054 | 0.537 |
| Lactococcus | rs55910161 | C | T | 0.146 | 0.031 | 1.90E-06 | 0.001 | 0.085 | 0.993 |
| Lactococcus | rs2293361 | C | T | -0.199 | 0.043 | 3.79E-06 | -0.032 | 0.118 | 0.784 |
| Lactococcus | rs6674304 | C | T | 0.201 | 0.044 | 5.60E-06 | 0.034 | 0.140 | 0.809 |
| Lactococcus | rs12621813 | G | A | 0.108 | 0.024 | 6.24E-06 | 0.074 | 0.060 | 0.215 |
| Lactococcus | rs17168302 | G | A | 0.192 | 0.042 | 6.28E-06 | -0.106 | 0.086 | 0.218 |
| Lactococcus | rs7992246 | T | C | 0.104 | 0.023 | 6.29E-06 | -0.053 | 0.054 | 0.332 |
| Marvinbryantia | rs2724813 | A | G | -0.084 | 0.017 | 5.22E-07 | 0.017 | 0.063 | 0.784 |
| Marvinbryantia | rs61884471 | G | A | 0.124 | 0.025 | 5.49E-07 | 0.019 | 0.085 | 0.823 |
| Marvinbryantia | rs2842896 | C | T | -0.065 | 0.013 | 7.36E-07 | 0.033 | 0.054 | 0.538 |
| Marvinbryantia | rs1187983 | C | T | -0.094 | 0.019 | 1.28E-06 | 0.111 | 0.088 | 0.208 |
| Marvinbryantia | rs2863363 | A | G | 0.063 | 0.014 | 3.21E-06 | -0.055 | 0.062 | 0.371 |
| Marvinbryantia | rs72948274 | A | C | -0.126 | 0.027 | 3.45E-06 | -0.005 | 0.109 | 0.963 |
| Marvinbryantia | rs3125832 | A | C | 0.068 | 0.015 | 6.03E-06 | -0.063 | 0.064 | 0.325 |
| Marvinbryantia | rs146541147 | G | A | 0.119 | 0.027 | 9.53E-06 | -0.086 | 0.150 | 0.567 |
| Marvinbryantia | rs11620597 | T | C | 0.119 | 0.027 | 1.09E-05 | 0.099 | 0.174 | 0.571 |
| Marvinbryantia | rs8006832 | G | T | -0.095 | 0.022 | 1.11E-05 | -0.030 | 0.093 | 0.744 |
| Methanobrevibacter | rs73457410 | A | G | 0.218 | 0.044 | 9.32E-07 | -0.058 | 0.109 | 0.597 |
| Methanobrevibacter | rs10202904 | T | G | -0.113 | 0.024 | 2.38E-06 | 0.055 | 0.054 | 0.307 |
| Methanobrevibacter | rs894996 | C | A | 0.214 | 0.046 | 2.64E-06 | 0.179 | 0.105 | 0.088 |
| Methanobrevibacter | rs1334944 | T | C | 0.115 | 0.026 | 6.52E-06 | -0.034 | 0.059 | 0.558 |
| Methanobrevibacter | rs6776814 | T | C | -0.189 | 0.042 | 6.80E-06 | 0.069 | 0.188 | 0.714 |
| Methanobrevibacter | rs4802933 | A | G | -0.136 | 0.031 | 1.07E-05 | -0.047 | 0.063 | 0.451 |
| Odoribacter | rs77779484 | G | A | -0.133 | 0.027 | 6.65E-07 | -0.051 | 0.112 | 0.645 |
| Odoribacter | rs10093869 | A | G | -0.058 | 0.013 | 4.07E-06 | -0.039 | 0.054 | 0.471 |
| Odoribacter | rs74553962 | T | G | 0.121 | 0.026 | 4.26E-06 | -0.028 | 0.101 | 0.778 |
| Odoribacter | rs10423795 | C | T | 0.055 | 0.012 | 5.49E-06 | -0.033 | 0.055 | 0.547 |
| Odoribacter | rs6856150 | G | A | 0.088 | 0.019 | 5.56E-06 | -0.136 | 0.081 | 0.094 |
| Odoribacter | rs28417404 | A | G | -0.073 | 0.016 | 6.65E-06 | -0.023 | 0.090 | 0.795 |
| Odoribacter | rs4793970 | A | G | -0.058 | 0.013 | 8.11E-06 | 0.008 | 0.055 | 0.887 |
| Olsenella | rs62112538 | C | T | -0.199 | 0.041 | 9.60E-07 | 0.148 | 0.084 | 0.079 |
| Olsenella | rs72691585 | C | A | -0.249 | 0.052 | 1.73E-06 | 0.144 | 0.077 | 0.063 |
| Olsenella | rs17148768 | G | A | 0.140 | 0.030 | 2.03E-06 | -0.024 | 0.071 | 0.740 |
| Olsenella | rs2759329 | G | A | -0.111 | 0.024 | 2.80E-06 | 0.021 | 0.055 | 0.700 |
| Olsenella | rs35225860 | A | G | -0.224 | 0.048 | 3.56E-06 | -0.044 | 0.138 | 0.752 |
| Olsenella | rs7540303 | C | T | 0.108 | 0.024 | 4.86E-06 | 0.013 | 0.055 | 0.818 |
| Olsenella | rs1035588 | A | G | -0.108 | 0.024 | 4.97E-06 | -0.001 | 0.055 | 0.980 |
| Olsenella | rs61090148 | A | G | -0.105 | 0.023 | 5.92E-06 | 0.008 | 0.054 | 0.882 |
| Olsenella | rs9460691 | C | A | 0.120 | 0.027 | 7.98E-06 | -0.060 | 0.068 | 0.375 |
| Olsenella | rs8066522 | G | A | -0.107 | 0.024 | 9.35E-06 | 0.021 | 0.057 | 0.717 |
| Oscillibacter | rs11627628 | T | C | 0.144 | 0.029 | 7.04E-07 | -0.076 | 0.100 | 0.447 |
| Oscillibacter | rs9393920 | A | G | -0.074 | 0.015 | 8.27E-07 | 0.065 | 0.056 | 0.249 |
| Oscillibacter | rs234108 | A | G | 0.075 | 0.015 | 9.07E-07 | 0.063 | 0.054 | 0.241 |
| Oscillibacter | rs133832 | A | C | -0.080 | 0.016 | 9.67E-07 | 0.019 | 0.059 | 0.743 |
| Oscillibacter | rs36095275 | C | T | -0.075 | 0.016 | 1.62E-06 | -0.001 | 0.054 | 0.979 |
| Oscillibacter | rs16866406 | A | G | 0.099 | 0.021 | 2.18E-06 | 0.004 | 0.073 | 0.956 |
| Oscillibacter | rs12649930 | T | G | 0.122 | 0.026 | 2.82E-06 | 0.127 | 0.088 | 0.148 |
| Oscillibacter | rs4506202 | A | G | -0.071 | 0.015 | 2.99E-06 | 0.047 | 0.053 | 0.378 |
| Oscillibacter | rs16934185 | A | G | -0.130 | 0.028 | 4.19E-06 | -0.074 | 0.087 | 0.397 |
| Oscillibacter | rs61883564 | A | G | -0.101 | 0.022 | 4.52E-06 | 0.027 | 0.077 | 0.731 |
| Oscillibacter | rs11990279 | T | C | -0.082 | 0.018 | 4.85E-06 | -0.018 | 0.077 | 0.817 |
| Oscillibacter | rs75453768 | G | T | 0.122 | 0.027 | 5.47E-06 | -0.117 | 0.089 | 0.188 |
| Oscillibacter | rs761240 | T | G | -0.177 | 0.039 | 5.54E-06 | 0.164 | 0.123 | 0.183 |
| Oscillospira | rs12206468 | G | A | -0.133 | 0.027 | 8.17E-07 | 0.034 | 0.099 | 0.728 |
| Oscillospira | rs1954532 | T | C | -0.083 | 0.018 | 2.42E-06 | -0.084 | 0.067 | 0.210 |
| Oscillospira | rs72866977 | A | C | -0.131 | 0.028 | 3.56E-06 | -0.024 | 0.098 | 0.808 |
| Oscillospira | rs8076323 | A | G | 0.072 | 0.016 | 4.88E-06 | -0.028 | 0.056 | 0.625 |
| Oscillospira | rs62422654 | C | T | 0.090 | 0.020 | 5.74E-06 | -0.060 | 0.065 | 0.355 |
| Oscillospira | rs28889936 | A | C | 0.114 | 0.025 | 6.46E-06 | 0.131 | 0.090 | 0.144 |
| Oscillospira | rs751183 | T | C | -0.077 | 0.017 | 6.92E-06 | -0.116 | 0.069 | 0.091 |
| Oxalobacter | rs736744 | C | T | 0.118 | 0.021 | 2.41E-08 | -0.044 | 0.053 | 0.411 |
| Oxalobacter | rs4428215 | G | A | 0.130 | 0.024 | 7.50E-08 | 0.028 | 0.061 | 0.650 |
| Oxalobacter | rs6000536 | C | T | -0.131 | 0.025 | 2.45E-07 | -0.023 | 0.071 | 0.744 |
| Oxalobacter | rs36057338 | G | T | 0.208 | 0.042 | 8.15E-07 | -0.216 | 0.149 | 0.147 |
| Oxalobacter | rs10464997 | G | A | 0.138 | 0.029 | 3.00E-06 | -0.003 | 0.068 | 0.964 |
| Oxalobacter | rs11108500 | A | G | -0.199 | 0.043 | 3.17E-06 | -0.068 | 0.094 | 0.468 |
| Oxalobacter | rs12002250 | A | C | 0.217 | 0.047 | 3.22E-06 | 0.088 | 0.131 | 0.504 |
| Oxalobacter | rs1569853 | T | C | -0.138 | 0.030 | 3.33E-06 | -0.019 | 0.081 | 0.812 |
| Oxalobacter | rs6993398 | G | A | 0.127 | 0.028 | 5.06E-06 | -0.139 | 0.068 | 0.043 |
| Oxalobacter | rs111966731 | T | C | 0.213 | 0.047 | 6.22E-06 | -0.079 | 0.094 | 0.406 |
| Oxalobacter | rs3862635 | C | T | -0.172 | 0.039 | 1.25E-05 | -0.087 | 0.092 | 0.343 |
| Parabacteroides | rs60884758 | C | T | -0.070 | 0.014 | 7.82E-07 | 0.152 | 0.069 | 0.028 |
| Parabacteroides | rs4236095 | G | A | 0.076 | 0.016 | 1.22E-06 | -0.018 | 0.088 | 0.836 |
| Parabacteroides | rs6657302 | T | C | -0.105 | 0.023 | 3.58E-06 | 0.092 | 0.108 | 0.395 |
| Parabacteroides | rs115602804 | G | A | 0.103 | 0.022 | 3.69E-06 | 0.061 | 0.086 | 0.474 |
| Parabacteroides | rs7298818 | C | T | 0.089 | 0.020 | 9.68E-06 | -0.064 | 0.087 | 0.462 |
| Paraprevotella | rs2081023 | A | G | -0.123 | 0.024 | 2.19E-07 | -0.068 | 0.076 | 0.367 |
| Paraprevotella | rs9900242 | A | G | -0.085 | 0.018 | 1.13E-06 | -0.068 | 0.056 | 0.225 |
| Paraprevotella | rs9602779 | A | C | -0.107 | 0.022 | 1.27E-06 | 0.024 | 0.062 | 0.697 |
| Paraprevotella | rs4767113 | C | T | 0.088 | 0.018 | 1.58E-06 | 0.012 | 0.057 | 0.834 |
| Paraprevotella | rs4756632 | G | T | -0.139 | 0.029 | 1.65E-06 | 0.062 | 0.078 | 0.430 |
| Paraprevotella | rs145020347 | A | G | -0.125 | 0.026 | 2.02E-06 | 0.002 | 0.076 | 0.982 |
| Paraprevotella | rs17785622 | A | G | 0.248 | 0.052 | 2.23E-06 | -0.115 | 0.139 | 0.408 |
| Paraprevotella | rs3008582 | T | C | 0.106 | 0.023 | 3.28E-06 | -0.011 | 0.067 | 0.866 |
| Paraprevotella | rs58117850 | C | A | -0.150 | 0.032 | 3.68E-06 | -0.045 | 0.115 | 0.696 |
| Paraprevotella | rs17109926 | A | G | -0.099 | 0.022 | 4.83E-06 | 0.037 | 0.059 | 0.530 |
| Paraprevotella | rs3801748 | G | A | 0.078 | 0.017 | 5.59E-06 | 0.114 | 0.055 | 0.039 |
| Paraprevotella | rs7240324 | T | G | -0.102 | 0.023 | 6.57E-06 | -0.142 | 0.062 | 0.021 |
| Paraprevotella | rs10842464 | T | C | -0.076 | 0.017 | 1.11E-05 | 0.004 | 0.058 | 0.943 |
| Parasutterella | rs7572229 | G | A | 0.066 | 0.013 | 5.95E-07 | 0.094 | 0.053 | 0.077 |
| Parasutterella | rs78383039 | T | C | -0.146 | 0.030 | 8.46E-07 | -0.092 | 0.134 | 0.493 |
| Parasutterella | rs10899911 | A | G | -0.072 | 0.015 | 1.30E-06 | -0.025 | 0.063 | 0.689 |
| Parasutterella | rs7303158 | C | T | 0.065 | 0.013 | 1.45E-06 | 0.002 | 0.053 | 0.977 |
| Parasutterella | rs6828768 | C | T | 0.064 | 0.013 | 1.58E-06 | -0.003 | 0.053 | 0.949 |
| Parasutterella | rs2090816 | A | C | 0.084 | 0.018 | 2.11E-06 | -0.060 | 0.069 | 0.384 |
| Parasutterella | rs35055552 | T | C | 0.110 | 0.024 | 3.27E-06 | 0.007 | 0.077 | 0.930 |
| Parasutterella | rs8039785 | T | G | 0.062 | 0.013 | 3.33E-06 | 0.003 | 0.053 | 0.957 |
| Parasutterella | rs55877868 | A | C | -0.104 | 0.023 | 4.66E-06 | 0.018 | 0.088 | 0.834 |
| Parasutterella | rs62273907 | A | G | 0.229 | 0.050 | 4.91E-06 | -0.121 | 0.106 | 0.253 |
| Parasutterella | rs823424 | G | A | -0.071 | 0.016 | 5.48E-06 | -0.024 | 0.061 | 0.700 |
| Parasutterella | rs6809952 | G | A | -0.068 | 0.015 | 5.64E-06 | 0.001 | 0.060 | 0.993 |
| Parasutterella | rs11715853 | G | A | -0.066 | 0.015 | 5.70E-06 | -0.080 | 0.058 | 0.169 |
| Parasutterella | rs7311004 | T | C | -0.062 | 0.014 | 6.01E-06 | 0.088 | 0.053 | 0.097 |
| Peptococcus | rs10031059 | T | C | -0.121 | 0.023 | 8.09E-08 | -0.013 | 0.062 | 0.830 |
| Peptococcus | rs77681628 | C | T | 0.200 | 0.039 | 2.32E-07 | -0.152 | 0.096 | 0.115 |
| Peptococcus | rs11001948 | C | A | -0.191 | 0.038 | 5.35E-07 | -0.010 | 0.089 | 0.911 |
| Peptococcus | rs2054133 | G | A | 0.090 | 0.019 | 1.99E-06 | 0.032 | 0.056 | 0.568 |
| Peptococcus | rs7033353 | T | G | 0.090 | 0.019 | 2.07E-06 | 0.068 | 0.053 | 0.200 |
| Peptococcus | rs62424012 | G | A | 0.137 | 0.029 | 2.66E-06 | 0.096 | 0.112 | 0.390 |
| Peptococcus | rs413827 | G | A | 0.110 | 0.024 | 3.47E-06 | -0.059 | 0.062 | 0.343 |
| Peptococcus | rs36121075 | A | G | -0.141 | 0.031 | 4.37E-06 | 0.006 | 0.071 | 0.932 |
| Peptococcus | rs74592222 | G | A | 0.138 | 0.030 | 5.27E-06 | -0.112 | 0.084 | 0.179 |
| Peptococcus | rs5770862 | T | C | 0.162 | 0.036 | 5.61E-06 | 0.140 | 0.091 | 0.126 |
| Peptococcus | rs72850165 | T | C | -0.134 | 0.030 | 7.80E-06 | -0.234 | 0.101 | 0.020 |
| Peptococcus | rs12069354 | C | T | 0.168 | 0.038 | 1.00E-05 | -0.079 | 0.111 | 0.474 |
| Phascolarctobacterium | rs75882962 | T | C | 0.097 | 0.019 | 3.72E-07 | -0.103 | 0.080 | 0.197 |
| Phascolarctobacterium | rs56157888 | A | C | 0.095 | 0.019 | 8.54E-07 | -0.102 | 0.064 | 0.112 |
| Phascolarctobacterium | rs56069061 | G | A | -0.111 | 0.023 | 1.40E-06 | 0.025 | 0.107 | 0.812 |
| Phascolarctobacterium | rs74540770 | G | A | -0.121 | 0.026 | 2.88E-06 | -0.034 | 0.097 | 0.723 |
| Phascolarctobacterium | rs12618201 | A | G | 0.064 | 0.014 | 3.44E-06 | -0.038 | 0.053 | 0.482 |
| Phascolarctobacterium | rs1264476 | T | G | 0.077 | 0.017 | 3.81E-06 | -0.071 | 0.068 | 0.295 |
| Phascolarctobacterium | rs130483 | A | G | 0.066 | 0.014 | 5.07E-06 | -0.024 | 0.054 | 0.653 |
| Phascolarctobacterium | rs7982713 | G | A | 0.073 | 0.016 | 8.42E-06 | 0.079 | 0.059 | 0.182 |
| Phascolarctobacterium | rs11929846 | T | C | -0.070 | 0.016 | 1.02E-05 | 0.058 | 0.064 | 0.366 |
| Phascolarctobacterium | rs28525131 | G | A | -0.119 | 0.027 | 1.03E-05 | -0.072 | 0.123 | 0.556 |
| Prevotella7 | rs57404562 | C | A | 0.155 | 0.032 | 8.67E-07 | -0.096 | 0.079 | 0.224 |
| Prevotella7 | rs9959718 | G | A | 0.133 | 0.028 | 1.36E-06 | 0.090 | 0.066 | 0.175 |
| Prevotella7 | rs9608249 | A | G | -0.158 | 0.034 | 2.55E-06 | -0.104 | 0.085 | 0.223 |
| Prevotella7 | rs385483 | A | G | 0.137 | 0.029 | 2.67E-06 | 0.058 | 0.065 | 0.370 |
| Prevotella7 | rs11035469 | A | G | -0.144 | 0.031 | 3.80E-06 | -0.035 | 0.063 | 0.575 |
| Prevotella7 | rs2240542 | C | T | 0.121 | 0.026 | 3.90E-06 | -0.074 | 0.060 | 0.219 |
| Prevotella7 | rs2918132 | C | T | -0.115 | 0.025 | 6.88E-06 | -0.046 | 0.055 | 0.405 |
| Prevotella7 | rs12195431 | T | C | 0.197 | 0.044 | 8.87E-06 | 0.027 | 0.090 | 0.766 |
| Prevotella7 | rs9426434 | T | C | -0.124 | 0.028 | 9.00E-06 | 0.034 | 0.056 | 0.538 |
| Prevotella7 | rs12124567 | A | G | -0.121 | 0.028 | 1.03E-05 | 0.064 | 0.066 | 0.339 |
| Prevotella7 | rs118038478 | A | G | 0.206 | 0.047 | 1.15E-05 | 0.007 | 0.104 | 0.945 |
| Prevotella9 | rs111509883 | T | C | 0.171 | 0.035 | 8.53E-07 | -0.025 | 0.085 | 0.767 |
| Prevotella9 | rs11685699 | C | T | -0.141 | 0.030 | 1.74E-06 | -0.005 | 0.099 | 0.960 |
| Prevotella9 | rs2683313 | A | G | -0.072 | 0.015 | 1.76E-06 | 0.042 | 0.057 | 0.464 |
| Prevotella9 | rs746764 | T | C | -0.092 | 0.019 | 2.15E-06 | 0.026 | 0.065 | 0.687 |
| Prevotella9 | rs117271932 | A | G | 0.208 | 0.044 | 2.30E-06 | -0.187 | 0.115 | 0.105 |
| Prevotella9 | rs1304512 | G | A | 0.076 | 0.017 | 4.41E-06 | 0.092 | 0.060 | 0.125 |
| Prevotella9 | rs9613013 | G | A | 0.092 | 0.020 | 5.99E-06 | -0.029 | 0.081 | 0.724 |
| Prevotella9 | rs7237249 | C | T | -0.082 | 0.018 | 6.10E-06 | 0.075 | 0.068 | 0.269 |
| Prevotella9 | rs72815774 | T | C | -0.176 | 0.039 | 7.38E-06 | 0.122 | 0.119 | 0.308 |
| Prevotella9 | rs2495052 | A | G | 0.084 | 0.019 | 8.58E-06 | 0.028 | 0.074 | 0.706 |
| Prevotella9 | rs2104588 | T | C | 0.106 | 0.024 | 8.98E-06 | -0.146 | 0.115 | 0.203 |
| Prevotella9 | rs4968431 | G | T | 0.064 | 0.014 | 8.99E-06 | 0.007 | 0.055 | 0.904 |
| Prevotella9 | rs9428102 | A | G | -0.078 | 0.018 | 9.71E-06 | 0.034 | 0.064 | 0.598 |
| Prevotella9 | rs12648235 | T | C | 0.079 | 0.018 | 9.72E-06 | -0.013 | 0.063 | 0.833 |
| Prevotella9 | rs7976209 | T | C | -0.087 | 0.020 | 1.09E-05 | 0.010 | 0.073 | 0.896 |
| RikenellaceaeRC9gutgroup | rs2900503 | G | T | -0.172 | 0.033 | 1.33E-07 | -0.033 | 0.072 | 0.650 |
| RikenellaceaeRC9gutgroup | rs2998141 | T | C | -0.136 | 0.029 | 3.28E-06 | -0.024 | 0.063 | 0.705 |
| RikenellaceaeRC9gutgroup | rs17582787 | A | G | -0.158 | 0.034 | 3.48E-06 | 0.078 | 0.071 | 0.276 |
| RikenellaceaeRC9gutgroup | rs9887954 | G | A | -0.115 | 0.025 | 3.98E-06 | 0.015 | 0.054 | 0.777 |
| RikenellaceaeRC9gutgroup | rs17032291 | T | C | -0.170 | 0.037 | 4.13E-06 | -0.025 | 0.080 | 0.761 |
| RikenellaceaeRC9gutgroup | rs4717843 | G | T | -0.119 | 0.026 | 4.61E-06 | -0.057 | 0.053 | 0.286 |
| RikenellaceaeRC9gutgroup | rs80309088 | G | A | 0.174 | 0.038 | 5.64E-06 | -0.010 | 0.082 | 0.901 |
| RikenellaceaeRC9gutgroup | rs7712231 | A | G | 0.156 | 0.035 | 8.22E-06 | -0.016 | 0.078 | 0.841 |
| RikenellaceaeRC9gutgroup | rs12501673 | A | G | 0.116 | 0.026 | 9.07E-06 | 0.031 | 0.060 | 0.605 |
| RikenellaceaeRC9gutgroup | rs2074881 | T | C | -0.142 | 0.032 | 1.13E-05 | 0.021 | 0.078 | 0.791 |
| RikenellaceaeRC9gutgroup | rs4270579 | G | A | -0.118 | 0.027 | 1.33E-05 | 0.087 | 0.057 | 0.127 |
| Romboutsia | rs61841503 | G | A | 0.093 | 0.017 | 6.04E-08 | -0.128 | 0.079 | 0.104 |
| Romboutsia | rs10279978 | A | G | -0.062 | 0.013 | 1.10E-06 | -0.079 | 0.057 | 0.165 |
| Romboutsia | rs75987356 | G | A | -0.130 | 0.028 | 3.83E-06 | 0.121 | 0.100 | 0.226 |
| Romboutsia | rs11221428 | T | C | -0.073 | 0.016 | 4.39E-06 | -0.024 | 0.063 | 0.709 |
| Romboutsia | rs34302036 | A | G | 0.055 | 0.012 | 5.27E-06 | 0.058 | 0.054 | 0.280 |
| Romboutsia | rs9567264 | C | T | 0.058 | 0.013 | 5.38E-06 | -0.036 | 0.056 | 0.521 |
| Romboutsia | rs62504452 | A | G | -0.071 | 0.016 | 5.83E-06 | 0.003 | 0.077 | 0.969 |
| Romboutsia | rs75200530 | T | G | -0.191 | 0.042 | 5.96E-06 | -0.066 | 0.157 | 0.675 |
| Romboutsia | rs28603357 | T | C | -0.215 | 0.047 | 5.98E-06 | -0.037 | 0.191 | 0.848 |
| Romboutsia | rs77702691 | A | G | -0.094 | 0.021 | 5.99E-06 | 0.022 | 0.094 | 0.811 |
| Romboutsia | rs7109293 | A | G | 0.092 | 0.021 | 7.92E-06 | -0.128 | 0.083 | 0.124 |
| Romboutsia | rs9389266 | T | G | 0.072 | 0.016 | 8.50E-06 | -0.009 | 0.070 | 0.896 |
| Romboutsia | rs16843578 | C | T | -0.088 | 0.020 | 8.67E-06 | -0.075 | 0.119 | 0.529 |
| Roseburia | rs2160994 | T | C | 0.055 | 0.011 | 9.79E-07 | 0.062 | 0.056 | 0.271 |
| Roseburia | rs6930661 | C | T | -0.096 | 0.020 | 2.72E-06 | 0.158 | 0.111 | 0.152 |
| Roseburia | rs16910295 | T | C | -0.098 | 0.021 | 2.89E-06 | -0.148 | 0.118 | 0.208 |
| Roseburia | rs9300744 | C | T | -0.059 | 0.013 | 3.13E-06 | 0.031 | 0.069 | 0.655 |
| Roseburia | rs2943022 | T | C | 0.049 | 0.011 | 3.75E-06 | 0.038 | 0.054 | 0.486 |
| Roseburia | rs6445851 | G | A | -0.050 | 0.011 | 4.26E-06 | -0.045 | 0.055 | 0.409 |
| Roseburia | rs12740451 | T | C | 0.070 | 0.015 | 5.60E-06 | -0.020 | 0.078 | 0.795 |
| Roseburia | rs75326254 | C | T | -0.105 | 0.023 | 5.86E-06 | -0.102 | 0.113 | 0.365 |
| Roseburia | rs78753150 | A | C | 0.097 | 0.021 | 6.03E-06 | 0.111 | 0.088 | 0.205 |
| Roseburia | rs329182 | T | C | 0.069 | 0.015 | 6.32E-06 | 0.066 | 0.072 | 0.365 |
| Roseburia | rs302266 | T | C | -0.078 | 0.017 | 7.01E-06 | 0.064 | 0.079 | 0.417 |
| Roseburia | rs55858165 | A | C | 0.179 | 0.040 | 9.54E-06 | 0.048 | 0.141 | 0.733 |
| Roseburia | rs147990086 | A | G | -0.058 | 0.013 | 1.23E-05 | 0.114 | 0.071 | 0.106 |
| Roseburia | rs57466170 | C | T | 0.074 | 0.017 | 1.56E-05 | -0.048 | 0.102 | 0.635 |
| Ruminiclostridium5 | rs79968837 | A | G | -0.095 | 0.019 | 9.06E-07 | -0.110 | 0.121 | 0.366 |
| Ruminiclostridium5 | rs2482038 | C | A | 0.052 | 0.011 | 1.83E-06 | -0.018 | 0.054 | 0.739 |
| Ruminiclostridium5 | rs10827477 | A | G | -0.055 | 0.012 | 2.00E-06 | -0.086 | 0.055 | 0.120 |
| Ruminiclostridium5 | rs113753996 | T | C | 0.082 | 0.017 | 2.55E-06 | -0.050 | 0.068 | 0.463 |
| Ruminiclostridium5 | rs6121460 | G | A | 0.093 | 0.020 | 2.82E-06 | 0.089 | 0.098 | 0.365 |
| Ruminiclostridium5 | rs8053158 | A | G | -0.074 | 0.016 | 3.27E-06 | -0.021 | 0.082 | 0.795 |
| Ruminiclostridium5 | rs1492620 | T | C | -0.083 | 0.018 | 3.99E-06 | 0.014 | 0.079 | 0.857 |
| Ruminiclostridium5 | rs2791343 | T | C | 0.052 | 0.011 | 5.07E-06 | 0.044 | 0.054 | 0.418 |
| Ruminiclostridium5 | rs2833828 | G | A | 0.049 | 0.011 | 6.66E-06 | -0.002 | 0.054 | 0.978 |
| Ruminiclostridium5 | rs1223978 | T | C | 0.048 | 0.011 | 7.73E-06 | -0.100 | 0.054 | 0.061 |
| Ruminiclostridium5 | rs4955951 | A | G | -0.071 | 0.017 | 1.68E-05 | -0.167 | 0.083 | 0.043 |
| Ruminiclostridium6 | rs71414120 | T | G | 0.201 | 0.041 | 7.70E-07 | 0.113 | 0.121 | 0.352 |
| Ruminiclostridium6 | rs61060922 | T | G | 0.159 | 0.032 | 7.91E-07 | 0.061 | 0.147 | 0.681 |
| Ruminiclostridium6 | rs12362316 | A | G | 0.074 | 0.015 | 1.48E-06 | -0.047 | 0.063 | 0.453 |
| Ruminiclostridium6 | rs79968172 | G | A | 0.116 | 0.024 | 1.77E-06 | 0.103 | 0.114 | 0.365 |
| Ruminiclostridium6 | rs72991535 | T | G | 0.136 | 0.030 | 4.35E-06 | 0.051 | 0.147 | 0.726 |
| Ruminiclostridium6 | rs9555756 | A | C | -0.080 | 0.018 | 5.34E-06 | -0.036 | 0.096 | 0.705 |
| Ruminiclostridium6 | rs11992182 | A | C | 0.063 | 0.014 | 5.75E-06 | 0.020 | 0.064 | 0.758 |
| Ruminiclostridium6 | rs2548459 | C | T | 0.055 | 0.012 | 6.18E-06 | -0.042 | 0.054 | 0.430 |
| Ruminiclostridium6 | rs10829821 | T | C | -0.098 | 0.022 | 6.26E-06 | -0.166 | 0.093 | 0.075 |
| Ruminiclostridium6 | rs67479537 | T | C | 0.119 | 0.026 | 7.04E-06 | -0.008 | 0.123 | 0.947 |
| Ruminiclostridium6 | rs56212330 | A | G | 0.059 | 0.013 | 8.68E-06 | 0.048 | 0.060 | 0.421 |
| Ruminiclostridium6 | rs589368 | G | A | -0.147 | 0.033 | 8.81E-06 | 0.023 | 0.149 | 0.877 |
| Ruminiclostridium6 | rs116969552 | A | G | -0.167 | 0.038 | 9.48E-06 | -0.097 | 0.159 | 0.541 |
| Ruminiclostridium6 | rs792058 | G | A | 0.055 | 0.013 | 9.92E-06 | -0.053 | 0.054 | 0.328 |
| Ruminiclostridium6 | rs35362464 | C | A | 0.072 | 0.017 | 1.34E-05 | -0.071 | 0.076 | 0.351 |
| Ruminiclostridium9 | rs918449 | A | G | -0.095 | 0.020 | 1.42E-06 | -0.063 | 0.101 | 0.536 |
| Ruminiclostridium9 | rs12040548 | G | T | 0.057 | 0.012 | 3.13E-06 | -0.083 | 0.060 | 0.162 |
| Ruminiclostridium9 | rs7137760 | C | T | 0.051 | 0.011 | 5.95E-06 | -0.001 | 0.053 | 0.985 |
| Ruminiclostridium9 | rs9522712 | T | C | 0.070 | 0.015 | 6.30E-06 | 0.016 | 0.074 | 0.835 |
| Ruminiclostridium9 | rs78191726 | T | C | 0.094 | 0.021 | 6.94E-06 | -0.010 | 0.102 | 0.920 |
| Ruminiclostridium9 | rs9809789 | C | T | -0.072 | 0.016 | 7.01E-06 | 0.175 | 0.069 | 0.011 |
| Ruminiclostridium9 | rs6082461 | A | C | 0.059 | 0.013 | 7.59E-06 | 0.025 | 0.065 | 0.706 |
| Ruminiclostridium9 | rs74303178 | T | C | 0.053 | 0.012 | 7.95E-06 | -0.032 | 0.057 | 0.576 |
| RuminococcaceaeNK4A214group | rs5994253 | A | G | -0.081 | 0.016 | 2.64E-07 | 0.090 | 0.076 | 0.239 |
| RuminococcaceaeNK4A214group | rs11586410 | G | A | -0.086 | 0.017 | 3.76E-07 | -0.112 | 0.074 | 0.130 |
| RuminococcaceaeNK4A214group | rs136761 | G | A | -0.059 | 0.012 | 8.19E-07 | -0.049 | 0.055 | 0.372 |
| RuminococcaceaeNK4A214group | rs4814689 | C | T | -0.108 | 0.023 | 2.68E-06 | -0.096 | 0.127 | 0.449 |
| RuminococcaceaeNK4A214group | rs12642039 | T | C | -0.055 | 0.012 | 3.63E-06 | 0.075 | 0.055 | 0.174 |
| RuminococcaceaeNK4A214group | rs7573569 | T | C | 0.108 | 0.023 | 4.00E-06 | -0.143 | 0.110 | 0.195 |
| RuminococcaceaeNK4A214group | rs12731 | A | G | -0.053 | 0.012 | 4.51E-06 | 0.010 | 0.055 | 0.852 |
| RuminococcaceaeNK4A214group | rs13087692 | T | G | 0.057 | 0.013 | 5.05E-06 | 0.017 | 0.058 | 0.765 |
| RuminococcaceaeNK4A214group | rs35559912 | T | C | -0.093 | 0.020 | 5.57E-06 | 0.034 | 0.081 | 0.674 |
| RuminococcaceaeNK4A214group | rs147475196 | A | G | -0.134 | 0.030 | 5.86E-06 | -0.131 | 0.087 | 0.132 |
| RuminococcaceaeNK4A214group | rs62027366 | T | C | 0.062 | 0.014 | 7.75E-06 | 0.066 | 0.067 | 0.325 |
| RuminococcaceaeNK4A214group | rs11241747 | C | T | 0.053 | 0.012 | 8.69E-06 | 0.063 | 0.059 | 0.286 |
| RuminococcaceaeNK4A214group | rs6681678 | C | T | -0.100 | 0.024 | 2.99E-05 | 0.336 | 0.150 | 0.025 |
| RuminococcaceaeUCG002 | rs77564310 | A | C | -0.071 | 0.014 | 4.14E-07 | -0.027 | 0.065 | 0.679 |
| RuminococcaceaeUCG002 | rs55793120 | T | C | 0.137 | 0.027 | 5.39E-07 | 0.028 | 0.112 | 0.803 |
| RuminococcaceaeUCG002 | rs7155595 | C | A | 0.057 | 0.012 | 1.11E-06 | 0.028 | 0.058 | 0.626 |
| RuminococcaceaeUCG002 | rs10927423 | C | A | -0.071 | 0.015 | 1.36E-06 | 0.111 | 0.069 | 0.109 |
| RuminococcaceaeUCG002 | rs62374283 | T | C | -0.058 | 0.012 | 1.45E-06 | 0.007 | 0.055 | 0.895 |
| RuminococcaceaeUCG002 | rs116974815 | C | A | -0.190 | 0.040 | 1.72E-06 | -0.004 | 0.106 | 0.974 |
| RuminococcaceaeUCG002 | rs10916131 | C | T | -0.069 | 0.015 | 2.31E-06 | -0.081 | 0.072 | 0.266 |
| RuminococcaceaeUCG002 | rs79016051 | C | T | -0.089 | 0.019 | 2.78E-06 | -0.005 | 0.079 | 0.946 |
| RuminococcaceaeUCG002 | rs12463378 | A | G | -0.052 | 0.011 | 3.20E-06 | 0.058 | 0.058 | 0.318 |
| RuminococcaceaeUCG002 | rs7120052 | A | C | 0.062 | 0.014 | 4.02E-06 | -0.045 | 0.068 | 0.506 |
| RuminococcaceaeUCG002 | rs113147300 | A | G | -0.076 | 0.016 | 4.05E-06 | 0.013 | 0.078 | 0.869 |
| RuminococcaceaeUCG002 | rs76847269 | A | G | 0.164 | 0.036 | 4.41E-06 | -0.297 | 0.169 | 0.078 |
| RuminococcaceaeUCG002 | rs7342369 | C | A | -0.053 | 0.012 | 5.39E-06 | -0.011 | 0.061 | 0.855 |
| RuminococcaceaeUCG002 | rs56030423 | G | A | -0.098 | 0.022 | 5.54E-06 | 0.040 | 0.103 | 0.697 |
| RuminococcaceaeUCG002 | rs882348 | A | G | -0.080 | 0.018 | 7.52E-06 | -0.030 | 0.083 | 0.719 |
| RuminococcaceaeUCG002 | rs6542556 | A | G | 0.051 | 0.011 | 7.86E-06 | 0.018 | 0.055 | 0.736 |
| RuminococcaceaeUCG002 | rs6793778 | C | T | -0.056 | 0.013 | 8.18E-06 | 0.047 | 0.060 | 0.432 |
| RuminococcaceaeUCG002 | rs7249614 | A | G | -0.049 | 0.011 | 8.70E-06 | -0.107 | 0.055 | 0.049 |
| RuminococcaceaeUCG002 | rs57079348 | T | G | -0.077 | 0.017 | 9.39E-06 | -0.098 | 0.116 | 0.401 |
| RuminococcaceaeUCG002 | rs11607472 | A | G | -0.078 | 0.018 | 9.65E-06 | 0.023 | 0.106 | 0.828 |
| RuminococcaceaeUCG002 | rs15256 | C | T | 0.073 | 0.017 | 1.36E-05 | -0.050 | 0.081 | 0.542 |
| RuminococcaceaeUCG002 | rs10964441 | G | A | -0.149 | 0.034 | 1.54E-05 | 0.072 | 0.087 | 0.408 |
| RuminococcaceaeUCG003 | rs73341548 | T | G | -0.170 | 0.032 | 9.50E-08 | 0.045 | 0.114 | 0.692 |
| RuminococcaceaeUCG003 | rs6759615 | A | G | 0.103 | 0.020 | 3.04E-07 | 0.008 | 0.089 | 0.926 |
| RuminococcaceaeUCG003 | rs646327 | G | A | 0.059 | 0.012 | 7.18E-07 | -0.040 | 0.054 | 0.460 |
| RuminococcaceaeUCG003 | rs11243416 | T | C | -0.093 | 0.019 | 1.29E-06 | -0.011 | 0.106 | 0.917 |
| RuminococcaceaeUCG003 | rs16959793 | A | C | -0.063 | 0.013 | 1.90E-06 | 0.053 | 0.054 | 0.327 |
| RuminococcaceaeUCG003 | rs4452755 | A | C | -0.063 | 0.013 | 2.49E-06 | 0.008 | 0.056 | 0.894 |
| RuminococcaceaeUCG003 | rs11831525 | A | C | 0.071 | 0.015 | 2.71E-06 | -0.066 | 0.064 | 0.297 |
| RuminococcaceaeUCG003 | rs10490280 | C | T | -0.067 | 0.014 | 2.73E-06 | -0.020 | 0.068 | 0.769 |
| RuminococcaceaeUCG003 | rs78720113 | A | G | -0.115 | 0.025 | 3.81E-06 | 0.206 | 0.099 | 0.038 |
| RuminococcaceaeUCG003 | rs3013089 | G | A | -0.055 | 0.012 | 4.60E-06 | 0.051 | 0.055 | 0.355 |
| RuminococcaceaeUCG003 | rs2523124 | T | C | -0.055 | 0.012 | 6.06E-06 | 0.030 | 0.054 | 0.575 |
| RuminococcaceaeUCG003 | rs4532474 | G | A | 0.077 | 0.017 | 6.39E-06 | 0.004 | 0.072 | 0.956 |
| RuminococcaceaeUCG004 | rs6769553 | A | G | 0.085 | 0.016 | 6.78E-08 | 0.015 | 0.060 | 0.804 |
| RuminococcaceaeUCG004 | rs12125734 | G | T | 0.134 | 0.026 | 1.95E-07 | -0.086 | 0.091 | 0.340 |
| RuminococcaceaeUCG004 | rs516741 | T | G | -0.076 | 0.016 | 3.09E-06 | -0.071 | 0.068 | 0.294 |
| RuminococcaceaeUCG004 | rs9818949 | G | T | 0.086 | 0.019 | 5.17E-06 | 0.005 | 0.067 | 0.939 |
| RuminococcaceaeUCG004 | rs3800178 | C | T | -0.080 | 0.018 | 6.15E-06 | 0.148 | 0.061 | 0.015 |
| RuminococcaceaeUCG004 | rs2248146 | T | C | 0.069 | 0.015 | 7.22E-06 | -0.102 | 0.056 | 0.067 |
| RuminococcaceaeUCG004 | rs872501 | G | A | 0.116 | 0.026 | 7.62E-06 | -0.102 | 0.100 | 0.305 |
| RuminococcaceaeUCG004 | rs10976229 | T | G | 0.096 | 0.021 | 7.70E-06 | -0.185 | 0.079 | 0.019 |
| RuminococcaceaeUCG004 | rs7569771 | A | G | -0.076 | 0.017 | 8.29E-06 | -0.053 | 0.061 | 0.390 |
| RuminococcaceaeUCG004 | rs11961899 | G | A | -0.071 | 0.016 | 1.15E-05 | -0.054 | 0.059 | 0.358 |
| RuminococcaceaeUCG004 | rs550351 | A | C | 0.079 | 0.018 | 1.27E-05 | 0.050 | 0.054 | 0.355 |
| RuminococcaceaeUCG005 | rs10950694 | T | C | 0.058 | 0.011 | 4.16E-07 | -0.005 | 0.055 | 0.931 |
| RuminococcaceaeUCG005 | rs34781347 | G | A | 0.189 | 0.039 | 1.05E-06 | -0.068 | 0.103 | 0.506 |
| RuminococcaceaeUCG005 | rs2893871 | G | A | -0.074 | 0.016 | 2.17E-06 | -0.123 | 0.084 | 0.146 |
| RuminococcaceaeUCG005 | rs12604884 | G | T | 0.068 | 0.014 | 2.63E-06 | 0.063 | 0.069 | 0.362 |
| RuminococcaceaeUCG005 | rs7555878 | A | G | 0.059 | 0.013 | 2.83E-06 | -0.056 | 0.062 | 0.366 |
| RuminococcaceaeUCG005 | rs114279581 | A | G | -0.147 | 0.032 | 3.50E-06 | 0.020 | 0.100 | 0.838 |
| RuminococcaceaeUCG005 | rs12288512 | A | G | 0.067 | 0.014 | 3.91E-06 | 0.055 | 0.062 | 0.376 |
| RuminococcaceaeUCG005 | rs7449320 | C | A | 0.060 | 0.013 | 4.66E-06 | 0.030 | 0.063 | 0.636 |
| RuminococcaceaeUCG005 | rs10873449 | T | C | 0.065 | 0.014 | 5.41E-06 | 0.036 | 0.067 | 0.588 |
| RuminococcaceaeUCG005 | rs10937802 | G | A | 0.076 | 0.017 | 7.03E-06 | -0.029 | 0.082 | 0.726 |
| RuminococcaceaeUCG005 | rs7586445 | G | A | 0.078 | 0.018 | 9.27E-06 | -0.089 | 0.079 | 0.258 |
| RuminococcaceaeUCG005 | rs72776570 | C | A | 0.087 | 0.020 | 1.01E-05 | -0.001 | 0.089 | 0.993 |
| RuminococcaceaeUCG005 | rs55793120 | T | C | 0.122 | 0.028 | 1.37E-05 | 0.028 | 0.112 | 0.803 |
| RuminococcaceaeUCG005 | rs898577 | T | C | -0.123 | 0.029 | 1.78E-05 | 0.025 | 0.115 | 0.827 |
| RuminococcaceaeUCG009 | rs1550196 | G | A | 0.131 | 0.026 | 6.20E-07 | -0.275 | 0.089 | 0.002 |
| RuminococcaceaeUCG009 | rs4708333 | T | G | -0.084 | 0.017 | 1.52E-06 | -0.021 | 0.056 | 0.704 |
| RuminococcaceaeUCG009 | rs758191 | T | G | 0.177 | 0.038 | 2.37E-06 | -0.123 | 0.089 | 0.168 |
| RuminococcaceaeUCG009 | rs2058609 | A | G | 0.082 | 0.017 | 2.97E-06 | -0.092 | 0.059 | 0.121 |
| RuminococcaceaeUCG009 | rs2192926 | A | G | -0.089 | 0.019 | 3.95E-06 | 0.030 | 0.056 | 0.598 |
| RuminococcaceaeUCG009 | rs4079028 | C | T | 0.092 | 0.020 | 4.37E-06 | 0.036 | 0.062 | 0.565 |
| RuminococcaceaeUCG009 | rs12508214 | C | T | -0.077 | 0.017 | 4.53E-06 | 0.068 | 0.057 | 0.232 |
| RuminococcaceaeUCG009 | rs9558661 | T | C | -0.090 | 0.020 | 7.73E-06 | 0.057 | 0.066 | 0.390 |
| RuminococcaceaeUCG009 | rs113006825 | T | C | -0.093 | 0.021 | 8.00E-06 | 0.068 | 0.065 | 0.294 |
| RuminococcaceaeUCG009 | rs138460696 | A | G | 0.139 | 0.032 | 1.03E-05 | 0.182 | 0.101 | 0.072 |
| RuminococcaceaeUCG009 | rs6952765 | G | A | 0.073 | 0.017 | 1.14E-05 | -0.039 | 0.057 | 0.494 |
| RuminococcaceaeUCG009 | rs78410648 | A | G | 0.121 | 0.028 | 1.28E-05 | 0.050 | 0.085 | 0.558 |
| RuminococcaceaeUCG010 | rs682403 | A | G | -0.059 | 0.012 | 2.39E-06 | -0.019 | 0.053 | 0.717 |
| RuminococcaceaeUCG010 | rs2820282 | A | C | -0.059 | 0.013 | 2.55E-06 | -0.074 | 0.054 | 0.172 |
| RuminococcaceaeUCG010 | rs6958419 | C | T | -0.059 | 0.012 | 2.79E-06 | -0.032 | 0.053 | 0.553 |
| RuminococcaceaeUCG010 | rs12597105 | G | A | 0.067 | 0.014 | 3.40E-06 | -0.049 | 0.068 | 0.469 |
| RuminococcaceaeUCG010 | rs73218807 | G | A | -0.166 | 0.037 | 6.26E-06 | 0.133 | 0.097 | 0.173 |
| RuminococcaceaeUCG010 | rs7441445 | C | T | -0.057 | 0.013 | 6.74E-06 | -0.048 | 0.053 | 0.371 |
| RuminococcaceaeUCG011 | rs1416041 | A | C | -0.182 | 0.034 | 8.12E-08 | -0.038 | 0.066 | 0.562 |
| RuminococcaceaeUCG011 | rs12724320 | C | T | -0.121 | 0.025 | 1.23E-06 | -0.002 | 0.054 | 0.968 |
| RuminococcaceaeUCG011 | rs79113084 | C | T | -0.152 | 0.032 | 1.65E-06 | 0.026 | 0.088 | 0.772 |
| RuminococcaceaeUCG011 | rs12636310 | G | A | 0.133 | 0.028 | 2.53E-06 | -0.032 | 0.061 | 0.604 |
| RuminococcaceaeUCG011 | rs9729514 | A | G | 0.185 | 0.039 | 2.78E-06 | 0.003 | 0.091 | 0.972 |
| RuminococcaceaeUCG011 | rs2729556 | C | T | -0.109 | 0.023 | 3.04E-06 | 0.015 | 0.053 | 0.776 |
| RuminococcaceaeUCG011 | rs10274562 | C | T | 0.111 | 0.024 | 5.75E-06 | 0.051 | 0.054 | 0.352 |
| RuminococcaceaeUCG011 | rs4490371 | T | C | -0.112 | 0.025 | 7.08E-06 | -0.010 | 0.054 | 0.848 |
| RuminococcaceaeUCG013 | rs12781711 | C | T | -0.066 | 0.012 | 2.33E-08 | -0.008 | 0.061 | 0.898 |
| RuminococcaceaeUCG013 | rs12189346 | G | A | 0.068 | 0.015 | 2.54E-06 | 0.020 | 0.068 | 0.772 |
| RuminococcaceaeUCG013 | rs75088940 | T | C | -0.094 | 0.020 | 2.63E-06 | 0.026 | 0.105 | 0.804 |
| RuminococcaceaeUCG013 | rs76973485 | G | T | 0.195 | 0.042 | 3.13E-06 | -0.116 | 0.135 | 0.389 |
| RuminococcaceaeUCG013 | rs12485353 | G | A | -0.061 | 0.013 | 3.39E-06 | -0.010 | 0.061 | 0.877 |
| RuminococcaceaeUCG013 | rs16918863 | A | C | 0.111 | 0.024 | 3.44E-06 | -0.177 | 0.108 | 0.103 |
| RuminococcaceaeUCG013 | rs11581881 | C | T | 0.066 | 0.014 | 4.92E-06 | 0.007 | 0.063 | 0.913 |
| RuminococcaceaeUCG013 | rs4385846 | G | T | 0.060 | 0.013 | 5.63E-06 | 0.045 | 0.067 | 0.505 |
| RuminococcaceaeUCG013 | rs12336782 | T | C | -0.086 | 0.019 | 6.13E-06 | 0.211 | 0.099 | 0.033 |
| RuminococcaceaeUCG013 | rs9313055 | T | C | 0.105 | 0.023 | 7.39E-06 | -0.100 | 0.097 | 0.305 |
| RuminococcaceaeUCG013 | rs7784330 | G | A | -0.050 | 0.011 | 8.72E-06 | -0.006 | 0.055 | 0.916 |
| RuminococcaceaeUCG013 | rs2730183 | G | A | -0.049 | 0.011 | 8.73E-06 | 0.029 | 0.054 | 0.591 |
| RuminococcaceaeUCG014 | rs115777838 | T | C | -0.188 | 0.039 | 1.11E-06 | 0.143 | 0.084 | 0.088 |
| RuminococcaceaeUCG014 | rs12638134 | T | G | 0.058 | 0.012 | 1.12E-06 | 0.022 | 0.053 | 0.674 |
| RuminococcaceaeUCG014 | rs72809222 | T | C | 0.067 | 0.014 | 1.56E-06 | -0.169 | 0.065 | 0.009 |
| RuminococcaceaeUCG014 | rs995642 | C | T | 0.060 | 0.013 | 2.03E-06 | 0.084 | 0.062 | 0.177 |
| RuminococcaceaeUCG014 | rs10941294 | C | T | -0.122 | 0.026 | 2.68E-06 | 0.030 | 0.118 | 0.796 |
| RuminococcaceaeUCG014 | rs56105232 | G | A | 0.139 | 0.030 | 3.22E-06 | 0.136 | 0.109 | 0.210 |
| RuminococcaceaeUCG014 | rs73186226 | G | A | -0.099 | 0.022 | 4.57E-06 | 0.015 | 0.103 | 0.882 |
| RuminococcaceaeUCG014 | rs10791168 | A | G | -0.066 | 0.015 | 9.48E-06 | 0.056 | 0.069 | 0.418 |
| RuminococcaceaeUCG014 | rs853612 | A | G | -0.053 | 0.012 | 9.62E-06 | 0.015 | 0.054 | 0.782 |
| RuminococcaceaeUCG014 | rs34402072 | C | T | -0.069 | 0.016 | 1.04E-05 | 0.046 | 0.076 | 0.544 |
| RuminococcaceaeUCG014 | rs10495392 | C | T | -0.082 | 0.019 | 1.05E-05 | 0.063 | 0.102 | 0.538 |
| Ruminococcus1 | rs7117576 | A | G | 0.083 | 0.017 | 1.21E-06 | 0.133 | 0.092 | 0.149 |
| Ruminococcus1 | rs17781867 | C | T | 0.100 | 0.021 | 2.36E-06 | 0.242 | 0.104 | 0.020 |
| Ruminococcus1 | rs7583465 | C | T | 0.053 | 0.011 | 2.80E-06 | 0.127 | 0.053 | 0.017 |
| Ruminococcus1 | rs6493760 | C | T | 0.054 | 0.012 | 3.86E-06 | -0.085 | 0.056 | 0.125 |
| Ruminococcus1 | rs78613526 | G | A | 0.167 | 0.037 | 5.21E-06 | 0.010 | 0.122 | 0.936 |
| Ruminococcus1 | rs11783695 | G | T | -0.073 | 0.016 | 5.40E-06 | -0.058 | 0.073 | 0.428 |
| Ruminococcus1 | rs6105066 | T | C | -0.061 | 0.013 | 6.13E-06 | -0.039 | 0.060 | 0.517 |
| Ruminococcus1 | rs78572139 | G | A | 0.125 | 0.028 | 7.64E-06 | 0.047 | 0.088 | 0.597 |
| Ruminococcus1 | rs6750529 | C | T | 0.057 | 0.013 | 7.79E-06 | -0.029 | 0.061 | 0.631 |
| Ruminococcus1 | rs3819978 | C | T | -0.115 | 0.026 | 9.77E-06 | -0.053 | 0.099 | 0.593 |
| Ruminococcus2 | rs78120384 | A | G | -0.193 | 0.039 | 8.73E-07 | -0.043 | 0.092 | 0.638 |
| Ruminococcus2 | rs7635831 | G | A | 0.062 | 0.013 | 1.65E-06 | 0.082 | 0.055 | 0.138 |
| Ruminococcus2 | rs75140805 | T | G | 0.084 | 0.018 | 2.11E-06 | -0.126 | 0.070 | 0.070 |
| Ruminococcus2 | rs2997412 | A | G | -0.057 | 0.012 | 3.37E-06 | 0.031 | 0.060 | 0.608 |
| Ruminococcus2 | rs4799823 | C | T | 0.084 | 0.018 | 4.38E-06 | -0.097 | 0.068 | 0.156 |
| Ruminococcus2 | rs55707116 | C | A | 0.087 | 0.019 | 4.74E-06 | -0.008 | 0.101 | 0.936 |
| Ruminococcus2 | rs1819812 | G | T | 0.084 | 0.018 | 5.22E-06 | 0.061 | 0.120 | 0.614 |
| Ruminococcus2 | rs2368224 | T | G | 0.200 | 0.044 | 5.28E-06 | -0.124 | 0.123 | 0.315 |
| Ruminococcus2 | rs4400279 | A | G | 0.055 | 0.012 | 5.42E-06 | 0.016 | 0.056 | 0.777 |
| Ruminococcus2 | rs58681734 | A | G | 0.072 | 0.016 | 6.73E-06 | 0.005 | 0.065 | 0.938 |
| Ruminococcus2 | rs2846589 | G | T | 0.052 | 0.012 | 7.26E-06 | -0.059 | 0.054 | 0.279 |
| Ruminococcus2 | rs61791565 | T | C | -0.052 | 0.012 | 7.83E-06 | 0.051 | 0.053 | 0.335 |
| Ruminococcus2 | rs12406309 | A | C | -0.063 | 0.014 | 8.93E-06 | 0.077 | 0.064 | 0.230 |
| Ruminococcus2 | rs7693984 | G | A | -0.103 | 0.024 | 1.22E-05 | -0.023 | 0.130 | 0.859 |
| Ruminococcusgauvreauiigroup | rs71386687 | T | G | 0.121 | 0.024 | 3.92E-07 | -0.169 | 0.085 | 0.045 |
| Ruminococcusgauvreauiigroup | rs2047242 | A | G | -0.068 | 0.013 | 4.31E-07 | -0.012 | 0.063 | 0.846 |
| Ruminococcusgauvreauiigroup | rs9870933 | A | G | 0.062 | 0.013 | 8.19E-07 | -0.018 | 0.054 | 0.744 |
| Ruminococcusgauvreauiigroup | rs1391597 | C | T | 0.059 | 0.012 | 2.28E-06 | 0.017 | 0.054 | 0.748 |
| Ruminococcusgauvreauiigroup | rs289410 | G | A | -0.065 | 0.014 | 2.50E-06 | -0.026 | 0.059 | 0.660 |
| Ruminococcusgauvreauiigroup | rs10931481 | G | A | 0.061 | 0.013 | 2.93E-06 | 0.050 | 0.059 | 0.392 |
| Ruminococcusgauvreauiigroup | rs12539819 | C | T | 0.111 | 0.024 | 4.28E-06 | 0.104 | 0.107 | 0.331 |
| Ruminococcusgauvreauiigroup | rs2166943 | A | C | 0.057 | 0.012 | 4.41E-06 | -0.017 | 0.054 | 0.745 |
| Ruminococcusgauvreauiigroup | rs2105937 | A | G | 0.058 | 0.013 | 5.63E-06 | 0.037 | 0.056 | 0.513 |
| Ruminococcusgauvreauiigroup | rs11750752 | C | T | 0.071 | 0.016 | 5.64E-06 | 0.015 | 0.059 | 0.801 |
| Ruminococcusgauvreauiigroup | rs431418 | A | G | -0.095 | 0.021 | 6.55E-06 | -0.059 | 0.090 | 0.510 |
| Ruminococcusgauvreauiigroup | rs12079579 | A | G | 0.096 | 0.021 | 7.61E-06 | 0.167 | 0.096 | 0.082 |
| Ruminococcusgauvreauiigroup | rs73802842 | C | A | 0.074 | 0.017 | 1.41E-05 | -0.043 | 0.062 | 0.492 |
| Ruminococcusgnavusgroup | rs13163520 | G | A | -0.127 | 0.023 | 5.15E-08 | -0.044 | 0.069 | 0.520 |
| Ruminococcusgnavusgroup | rs2909242 | C | A | -0.091 | 0.018 | 7.10E-07 | -0.056 | 0.056 | 0.318 |
| Ruminococcusgnavusgroup | rs9872758 | T | C | 0.085 | 0.018 | 1.53E-06 | 0.029 | 0.053 | 0.589 |
| Ruminococcusgnavusgroup | rs62167033 | T | C | 0.185 | 0.040 | 2.93E-06 | 0.022 | 0.134 | 0.868 |
| Ruminococcusgnavusgroup | rs3124783 | A | G | -0.116 | 0.025 | 3.22E-06 | -0.028 | 0.079 | 0.722 |
| Ruminococcusgnavusgroup | rs12136548 | C | T | 0.090 | 0.020 | 4.42E-06 | -0.092 | 0.058 | 0.116 |
| Ruminococcusgnavusgroup | rs934940 | A | C | -0.105 | 0.023 | 4.75E-06 | 0.102 | 0.078 | 0.193 |
| Ruminococcusgnavusgroup | rs11597105 | A | G | 0.115 | 0.025 | 4.76E-06 | -0.003 | 0.067 | 0.961 |
| Ruminococcusgnavusgroup | rs12989336 | G | A | -0.085 | 0.019 | 6.60E-06 | -0.022 | 0.059 | 0.712 |
| Ruminococcusgnavusgroup | rs4388134 | C | T | -0.090 | 0.020 | 8.74E-06 | -0.007 | 0.059 | 0.912 |
| Ruminococcusgnavusgroup | rs78399089 | T | C | 0.144 | 0.033 | 9.76E-06 | 0.006 | 0.083 | 0.939 |
| Ruminococcusgnavusgroup | rs11864644 | T | C | -0.140 | 0.032 | 1.12E-05 | -0.158 | 0.083 | 0.058 |
| Ruminococcustorquesgroup | rs35866622 | T | C | -0.061 | 0.011 | 2.23E-08 | -0.050 | 0.056 | 0.372 |
| Ruminococcustorquesgroup | rs12434631 | A | G | 0.075 | 0.015 | 1.12E-06 | 0.021 | 0.086 | 0.811 |
| Ruminococcustorquesgroup | rs8080469 | G | A | 0.049 | 0.011 | 4.55E-06 | 0.042 | 0.053 | 0.424 |
| Ruminococcustorquesgroup | rs4073731 | T | C | 0.065 | 0.014 | 4.56E-06 | -0.064 | 0.072 | 0.370 |
| Ruminococcustorquesgroup | rs158487 | A | G | 0.053 | 0.012 | 4.86E-06 | -0.029 | 0.059 | 0.619 |
| Ruminococcustorquesgroup | rs77034621 | T | G | -0.152 | 0.034 | 6.42E-06 | -0.004 | 0.205 | 0.983 |
| Ruminococcustorquesgroup | rs10967781 | C | A | 0.051 | 0.011 | 7.37E-06 | -0.007 | 0.058 | 0.907 |
| Ruminococcustorquesgroup | rs8141465 | A | G | 0.048 | 0.011 | 7.65E-06 | -0.036 | 0.055 | 0.519 |
| Ruminococcustorquesgroup | rs1475330 | T | C | 0.052 | 0.012 | 9.47E-06 | 0.026 | 0.063 | 0.679 |
| Sellimonas | rs2371572 | A | C | 0.127 | 0.025 | 3.85E-07 | -0.044 | 0.053 | 0.409 |
| Sellimonas | rs13417181 | T | C | 0.167 | 0.034 | 8.09E-07 | 0.065 | 0.064 | 0.308 |
| Sellimonas | rs2016057 | A | C | -0.126 | 0.026 | 8.92E-07 | 0.090 | 0.054 | 0.094 |
| Sellimonas | rs2187447 | A | C | 0.243 | 0.053 | 3.96E-06 | -0.023 | 0.112 | 0.837 |
| Sellimonas | rs56203279 | T | C | -0.124 | 0.027 | 4.04E-06 | -0.040 | 0.056 | 0.471 |
| Sellimonas | rs113379006 | T | C | -0.163 | 0.036 | 5.15E-06 | -0.010 | 0.069 | 0.889 |
| Sellimonas | rs4600608 | A | G | -0.137 | 0.030 | 5.47E-06 | -0.054 | 0.065 | 0.409 |
| Sellimonas | rs41816 | A | G | 0.132 | 0.029 | 5.58E-06 | 0.031 | 0.058 | 0.589 |
| Sellimonas | rs553697 | T | C | -0.154 | 0.034 | 5.77E-06 | 0.038 | 0.068 | 0.580 |
| Senegalimassilia | rs11787826 | C | A | 0.081 | 0.017 | 2.02E-06 | -0.136 | 0.054 | 0.012 |
| Senegalimassilia | rs7225245 | G | A | 0.079 | 0.017 | 3.39E-06 | 0.042 | 0.054 | 0.440 |
| Senegalimassilia | rs10036909 | C | T | 0.186 | 0.040 | 3.70E-06 | 0.026 | 0.133 | 0.846 |
| Senegalimassilia | rs10221578 | G | A | 0.078 | 0.017 | 5.72E-06 | 0.026 | 0.053 | 0.626 |
| Senegalimassilia | rs1990708 | A | C | -0.110 | 0.025 | 9.69E-06 | 0.065 | 0.098 | 0.509 |
| Senegalimassilia | rs2017373 | C | T | 0.078 | 0.018 | 9.71E-06 | -0.043 | 0.055 | 0.429 |
| Slackia | rs8901 | C | T | 0.093 | 0.019 | 5.65E-07 | -0.039 | 0.058 | 0.505 |
| Slackia | rs12440440 | A | G | 0.090 | 0.019 | 2.22E-06 | -0.071 | 0.056 | 0.204 |
| Slackia | rs4492265 | A | G | -0.091 | 0.019 | 2.29E-06 | -0.015 | 0.058 | 0.801 |
| Slackia | rs16894137 | C | T | -0.123 | 0.026 | 3.04E-06 | -0.037 | 0.078 | 0.636 |
| Slackia | rs10409783 | A | G | 0.095 | 0.021 | 6.76E-06 | -0.015 | 0.059 | 0.801 |
| Slackia | rs35156985 | T | C | -0.156 | 0.035 | 7.70E-06 | -0.042 | 0.134 | 0.755 |
| Streptococcus | rs11110281 | T | C | -0.138 | 0.023 | 1.47E-09 | 0.162 | 0.125 | 0.194 |
| Streptococcus | rs957755 | T | G | -0.070 | 0.014 | 1.22E-06 | -0.032 | 0.076 | 0.672 |
| Streptococcus | rs10028567 | C | T | -0.092 | 0.019 | 1.58E-06 | 0.012 | 0.081 | 0.883 |
| Streptococcus | rs7916711 | A | G | 0.103 | 0.022 | 2.21E-06 | 0.091 | 0.076 | 0.232 |
| Streptococcus | rs11720390 | G | A | 0.107 | 0.023 | 2.71E-06 | -0.125 | 0.110 | 0.253 |
| Streptococcus | rs1918540 | G | A | 0.060 | 0.013 | 3.26E-06 | -0.028 | 0.069 | 0.689 |
| Streptococcus | rs17708276 | A | G | -0.079 | 0.017 | 3.27E-06 | -0.023 | 0.091 | 0.803 |
| Streptococcus | rs10448310 | A | G | -0.052 | 0.011 | 3.28E-06 | -0.035 | 0.055 | 0.525 |
| Streptococcus | rs6806351 | T | C | -0.063 | 0.014 | 3.51E-06 | -0.012 | 0.064 | 0.855 |
| Streptococcus | rs9895557 | T | C | -0.051 | 0.011 | 4.29E-06 | -0.018 | 0.053 | 0.731 |
| Streptococcus | rs9903102 | C | A | -0.071 | 0.016 | 4.90E-06 | 0.024 | 0.067 | 0.724 |
| Streptococcus | rs60486012 | G | A | -0.091 | 0.020 | 5.52E-06 | 0.277 | 0.137 | 0.043 |
| Streptococcus | rs71481756 | T | G | 0.093 | 0.021 | 7.56E-06 | 0.012 | 0.108 | 0.915 |
| Streptococcus | rs2370083 | G | T | -0.082 | 0.019 | 1.11E-05 | -0.042 | 0.108 | 0.694 |
| Subdoligranulum | rs6555306 | T | C | -0.074 | 0.016 | 1.89E-06 | 0.089 | 0.076 | 0.237 |
| Subdoligranulum | rs10065321 | T | C | -0.051 | 0.011 | 2.10E-06 | -0.040 | 0.054 | 0.458 |
| Subdoligranulum | rs4347804 | A | G | 0.166 | 0.036 | 3.40E-06 | 0.050 | 0.150 | 0.738 |
| Subdoligranulum | rs35940633 | G | A | -0.051 | 0.011 | 3.40E-06 | -0.002 | 0.057 | 0.977 |
| Subdoligranulum | rs76528319 | G | T | -0.143 | 0.031 | 3.99E-06 | -0.127 | 0.097 | 0.190 |
| Subdoligranulum | rs2171249 | C | T | 0.107 | 0.023 | 4.71E-06 | 0.100 | 0.104 | 0.339 |
| Subdoligranulum | rs3761728 | T | G | -0.054 | 0.012 | 4.83E-06 | 0.086 | 0.061 | 0.163 |
| Subdoligranulum | rs75158211 | T | C | -0.072 | 0.016 | 5.61E-06 | -0.053 | 0.074 | 0.476 |
| Subdoligranulum | rs1667315 | G | A | 0.049 | 0.011 | 6.37E-06 | 0.110 | 0.054 | 0.043 |
| Subdoligranulum | rs2114677 | C | T | -0.104 | 0.023 | 6.39E-06 | 0.082 | 0.082 | 0.316 |
| Subdoligranulum | rs10497836 | C | T | -0.052 | 0.012 | 1.01E-05 | 0.018 | 0.065 | 0.779 |
| Sutterella | rs2321387 | G | A | -0.059 | 0.012 | 1.92E-06 | -0.006 | 0.054 | 0.907 |
| Sutterella | rs143438747 | T | C | -0.146 | 0.031 | 2.02E-06 | 0.020 | 0.100 | 0.839 |
| Sutterella | rs13173038 | A | G | -0.072 | 0.015 | 2.18E-06 | 0.045 | 0.061 | 0.464 |
| Sutterella | rs7499539 | A | G | 0.062 | 0.013 | 2.43E-06 | -0.051 | 0.060 | 0.396 |
| Sutterella | rs62501473 | G | A | 0.069 | 0.015 | 3.38E-06 | -0.065 | 0.060 | 0.282 |
| Sutterella | rs11591622 | T | G | -0.069 | 0.015 | 5.43E-06 | -0.136 | 0.072 | 0.060 |
| Sutterella | rs1145877 | A | G | -0.074 | 0.016 | 5.94E-06 | -0.083 | 0.077 | 0.278 |
| Sutterella | rs7638039 | T | C | 0.065 | 0.014 | 7.22E-06 | -0.079 | 0.061 | 0.200 |
| Sutterella | rs2613606 | C | T | -0.056 | 0.012 | 7.26E-06 | -0.066 | 0.054 | 0.223 |
| Sutterella | rs607327 | C | T | 0.058 | 0.013 | 7.42E-06 | -0.012 | 0.055 | 0.829 |
| Sutterella | rs2050185 | G | A | 0.058 | 0.013 | 7.95E-06 | -0.136 | 0.055 | 0.013 |
| Sutterella | rs9350083 | T | G | -0.059 | 0.013 | 9.50E-06 | -0.050 | 0.055 | 0.370 |
| Terrisporobacter | rs1883097 | C | T | 0.226 | 0.045 | 6.37E-07 | -0.157 | 0.139 | 0.258 |
| Terrisporobacter | rs2872237 | C | A | -0.081 | 0.018 | 3.67E-06 | -0.022 | 0.054 | 0.683 |
| Terrisporobacter | rs58405430 | G | T | 0.135 | 0.030 | 7.83E-06 | 0.005 | 0.113 | 0.963 |
| Terrisporobacter | rs2569953 | A | C | -0.078 | 0.017 | 8.95E-06 | 0.093 | 0.054 | 0.084 |
| Terrisporobacter | rs7184125 | T | C | 0.091 | 0.021 | 8.96E-06 | -0.092 | 0.060 | 0.124 |
| Turicibacter | rs149744580 | A | G | 0.170 | 0.032 | 7.25E-08 | 0.064 | 0.119 | 0.589 |
| Turicibacter | rs12603364 | T | C | 0.111 | 0.023 | 8.92E-07 | 0.002 | 0.074 | 0.977 |
| Turicibacter | rs4869133 | G | A | 0.131 | 0.027 | 1.41E-06 | 0.063 | 0.070 | 0.369 |
| Turicibacter | rs55756211 | T | C | -0.115 | 0.024 | 1.73E-06 | -0.014 | 0.101 | 0.893 |
| Turicibacter | rs11054680 | T | C | -0.105 | 0.023 | 3.94E-06 | -0.061 | 0.070 | 0.386 |
| Turicibacter | rs2834977 | T | C | -0.096 | 0.021 | 4.03E-06 | -0.033 | 0.074 | 0.657 |
| Turicibacter | rs2952020 | G | A | -0.076 | 0.017 | 4.67E-06 | 0.053 | 0.062 | 0.387 |
| Turicibacter | rs7199484 | G | A | -0.073 | 0.016 | 4.96E-06 | 0.061 | 0.057 | 0.289 |
| Turicibacter | rs11666533 | C | T | -0.112 | 0.025 | 6.93E-06 | -0.006 | 0.098 | 0.949 |
| Tyzzerella3 | rs67476743 | T | G | 0.132 | 0.022 | 2.66E-09 | 0.092 | 0.060 | 0.126 |
| Tyzzerella3 | rs17706273 | T | C | -0.140 | 0.027 | 3.23E-07 | 0.068 | 0.099 | 0.490 |
| Tyzzerella3 | rs75091807 | G | T | -0.185 | 0.038 | 1.37E-06 | -0.146 | 0.111 | 0.189 |
| Tyzzerella3 | rs55799124 | A | G | -0.114 | 0.024 | 1.65E-06 | 0.063 | 0.060 | 0.294 |
| Tyzzerella3 | rs7019909 | T | C | 0.144 | 0.030 | 1.76E-06 | 0.122 | 0.082 | 0.134 |
| Tyzzerella3 | rs4904512 | T | C | -0.117 | 0.025 | 2.86E-06 | -0.043 | 0.079 | 0.582 |
| Tyzzerella3 | rs6920448 | C | T | -0.141 | 0.031 | 3.86E-06 | -0.023 | 0.092 | 0.806 |
| Tyzzerella3 | rs7561370 | T | C | 0.131 | 0.029 | 4.48E-06 | 0.019 | 0.074 | 0.795 |
| Tyzzerella3 | rs7333521 | T | C | -0.207 | 0.045 | 4.82E-06 | 0.179 | 0.150 | 0.233 |
| Tyzzerella3 | rs112102233 | A | G | -0.216 | 0.048 | 5.89E-06 | -0.012 | 0.128 | 0.922 |
| Tyzzerella3 | rs1232220 | G | T | -0.144 | 0.032 | 6.18E-06 | 0.009 | 0.090 | 0.924 |
| Tyzzerella3 | rs191093 | G | A | 0.159 | 0.035 | 6.78E-06 | -0.161 | 0.086 | 0.062 |
| Tyzzerella3 | rs10898797 | C | T | 0.122 | 0.027 | 8.38E-06 | 0.022 | 0.085 | 0.795 |
| Veillonella | rs1882878 | A | G | -0.077 | 0.016 | 2.71E-06 | 0.091 | 0.058 | 0.116 |
| Veillonella | rs62376424 | C | T | -0.076 | 0.016 | 3.13E-06 | 0.020 | 0.058 | 0.727 |
| Veillonella | rs2013594 | T | C | -0.072 | 0.016 | 3.40E-06 | -0.094 | 0.054 | 0.085 |
| Veillonella | rs742016 | A | G | -0.069 | 0.015 | 4.27E-06 | 0.023 | 0.056 | 0.688 |
| Veillonella | rs6656807 | A | G | 0.070 | 0.015 | 4.95E-06 | -0.085 | 0.055 | 0.123 |
| Veillonella | rs55807413 | A | G | 0.107 | 0.024 | 6.28E-06 | 0.122 | 0.088 | 0.167 |
| Victivallis | rs56349194 | A | G | -0.159 | 0.032 | 4.95E-07 | -0.020 | 0.080 | 0.802 |
| Victivallis | rs4764863 | G | A | 0.122 | 0.025 | 7.80E-07 | 0.067 | 0.053 | 0.207 |
| Victivallis | rs12512543 | A | C | -0.178 | 0.037 | 1.98E-06 | 0.109 | 0.095 | 0.251 |
| Victivallis | rs11899949 | G | A | 0.131 | 0.028 | 2.28E-06 | 0.057 | 0.057 | 0.318 |
| Victivallis | rs4895919 | T | C | -0.117 | 0.025 | 2.32E-06 | -0.138 | 0.053 | 0.009 |
| Victivallis | rs173120 | T | C | 0.134 | 0.029 | 3.99E-06 | -0.017 | 0.068 | 0.805 |
| Victivallis | rs911666 | T | C | -0.119 | 0.026 | 6.60E-06 | 0.009 | 0.058 | 0.882 |
| Victivallis | rs2546432 | T | C | -0.111 | 0.025 | 9.07E-06 | -0.014 | 0.053 | 0.791 |
| Victivallis | rs1882775 | A | G | -0.138 | 0.031 | 9.81E-06 | 0.103 | 0.069 | 0.132 |
| Victivallis | rs342302 | A | G | -0.153 | 0.035 | 1.39E-05 | -0.074 | 0.077 | 0.337 |

**Supplementary Table 2.** Full result of MR estimates for the association between gut microbiota and FM

| Bacterial taxa (exposure) | MR method | No. of SNP | Beta | Standard error | P-value | OR | Lower 95%CI | Upper 95%CI |
| --- | --- | --- | --- | --- | --- | --- | --- | --- |
| Actinomyces | IVW | 7 | -0.118 | 0.246 | 0.631 | 0.889 | 0.548 | 1.440 |
| Actinomyces | MR Egger | 7 | 0.151 | 0.601 | 0.811 | 1.163 | 0.358 | 3.782 |
| Actinomyces | Weighted median | 7 | -0.168 | 0.316 | 0.595 | 0.845 | 0.455 | 1.569 |
| Adlercreutzia | IVW | 8 | 0.002 | 0.376 | 0.996 | 1.002 | 0.480 | 2.092 |
| Adlercreutzia | MR Egger | 8 | 2.117 | 1.535 | 0.217 | 8.309 | 0.410 | 168.311 |
| Adlercreutzia | Weighted median | 8 | -0.340 | 0.407 | 0.403 | 0.711 | 0.320 | 1.580 |
| Akkermansia | IVW | 11 | 0.140 | 0.254 | 0.583 | 1.150 | 0.699 | 1.892 |
| Akkermansia | MR Egger | 11 | 0.105 | 0.858 | 0.906 | 1.110 | 0.207 | 5.967 |
| Akkermansia | Weighted median | 11 | 0.194 | 0.326 | 0.551 | 1.214 | 0.641 | 2.298 |
| Alistipes | IVW | 13 | -0.107 | 0.322 | 0.740 | 0.899 | 0.478 | 1.691 |
| Alistipes | MR Egger | 13 | -2.438 | 1.492 | 0.131 | 0.087 | 0.005 | 1.628 |
| Alistipes | Weighted median | 13 | -0.274 | 0.445 | 0.538 | 0.760 | 0.318 | 1.817 |
| Allisonella | IVW | 8 | 0.051 | 0.157 | 0.743 | 1.053 | 0.774 | 1.433 |
| Allisonella | MR Egger | 8 | 0.851 | 1.099 | 0.468 | 2.341 | 0.272 | 20.170 |
| Allisonella | Weighted median | 8 | -0.024 | 0.209 | 0.910 | 0.977 | 0.648 | 1.471 |
| Alloprevotella | IVW | 6 | -0.147 | 0.237 | 0.535 | 0.864 | 0.543 | 1.373 |
| Alloprevotella | MR Egger | 6 | -1.492 | 2.367 | 0.563 | 0.225 | 0.002 | 23.282 |
| Alloprevotella | Weighted median | 6 | -0.051 | 0.236 | 0.830 | 0.951 | 0.598 | 1.511 |
| Anaerofilum | IVW | 11 | -0.077 | 0.175 | 0.661 | 0.926 | 0.657 | 1.306 |
| Anaerofilum | MR Egger | 11 | -1.362 | 0.955 | 0.188 | 0.256 | 0.039 | 1.666 |
| Anaerofilum | Weighted median | 11 | -0.029 | 0.221 | 0.895 | 0.971 | 0.630 | 1.498 |
| Anaerostipes | IVW | 13 | -0.188 | 0.340 | 0.581 | 0.829 | 0.426 | 1.614 |
| Anaerostipes | MR Egger | 13 | -1.883 | 1.174 | 0.137 | 0.152 | 0.015 | 1.517 |
| Anaerostipes | Weighted median | 13 | -0.099 | 0.401 | 0.806 | 0.906 | 0.413 | 1.990 |
| Anaerotruncus | IVW | 13 | 0.129 | 0.283 | 0.649 | 1.137 | 0.653 | 1.980 |
| Anaerotruncus | MR Egger | 13 | -0.653 | 0.823 | 0.444 | 0.520 | 0.104 | 2.614 |
| Anaerotruncus | Weighted median | 13 | 0.062 | 0.373 | 0.868 | 1.064 | 0.512 | 2.212 |
| Bacteroides | IVW | 9 | -0.041 | 0.443 | 0.926 | 0.960 | 0.403 | 2.287 |
| Bacteroides | MR Egger | 9 | 0.868 | 2.430 | 0.732 | 2.382 | 0.020 | 279.064 |
| Bacteroides | Weighted median | 9 | -0.585 | 0.455 | 0.198 | 0.557 | 0.228 | 1.357 |
| Barnesiella | IVW | 13 | 0.196 | 0.264 | 0.458 | 1.217 | 0.725 | 2.043 |
| Barnesiella | MR Egger | 13 | 0.932 | 1.074 | 0.404 | 2.539 | 0.309 | 20.850 |
| Barnesiella | Weighted median | 13 | -0.064 | 0.369 | 0.863 | 0.938 | 0.455 | 1.934 |
| Bifidobacterium | IVW | 13 | 0.075 | 0.226 | 0.742 | 1.077 | 0.692 | 1.678 |
| Bifidobacterium | MR Egger | 13 | 0.998 | 0.575 | 0.110 | 2.714 | 0.880 | 8.372 |
| Bifidobacterium | Weighted median | 13 | -0.096 | 0.333 | 0.774 | 0.909 | 0.474 | 1.744 |
| Bilophila | IVW | 29 | 0.156 | 0.178 | 0.379 | 1.169 | 0.825 | 1.656 |
| Bilophila | MR Egger | 29 | -0.262 | 1.167 | 0.824 | 0.769 | 0.078 | 7.577 |
| Bilophila | Weighted median | 29 | 0.386 | 0.242 | 0.110 | 1.471 | 0.916 | 2.363 |
| Blautia | IVW | 13 | -0.307 | 0.301 | 0.308 | 0.736 | 0.408 | 1.327 |
| Blautia | MR Egger | 13 | 0.195 | 0.796 | 0.811 | 1.216 | 0.255 | 5.786 |
| Blautia | Weighted median | 13 | -0.234 | 0.422 | 0.579 | 0.791 | 0.346 | 1.809 |
| Butyricicoccus | IVW | 8 | -0.607 | 0.321 | 0.058 | 0.545 | 0.290 | 1.022 |
| Butyricicoccus | MR Egger | 8 | 0.238 | 0.625 | 0.716 | 1.269 | 0.373 | 4.317 |
| Butyricicoccus | Weighted median | 8 | -0.351 | 0.431 | 0.415 | 0.704 | 0.303 | 1.637 |
| Butyricimonas | IVW | 13 | 0.267 | 0.254 | 0.294 | 1.306 | 0.793 | 2.149 |
| Butyricimonas | MR Egger | 13 | -0.185 | 0.930 | 0.846 | 0.831 | 0.134 | 5.140 |
| Butyricimonas | Weighted median | 13 | -0.069 | 0.343 | 0.840 | 0.933 | 0.476 | 1.828 |
| Butyrivibrio | IVW | 15 | -0.017 | 0.118 | 0.888 | 0.984 | 0.781 | 1.239 |
| Butyrivibrio | MR Egger | 15 | -0.377 | 0.525 | 0.486 | 0.686 | 0.245 | 1.921 |
| Butyrivibrio | Weighted median | 15 | -0.037 | 0.165 | 0.823 | 0.964 | 0.697 | 1.332 |
| CandidatusSoleaferrea | IVW | 10 | -0.023 | 0.260 | 0.930 | 0.977 | 0.587 | 1.628 |
| CandidatusSoleaferrea | MR Egger | 10 | -1.269 | 2.966 | 0.680 | 0.281 | 0.001 | 94.149 |
| CandidatusSoleaferrea | Weighted median | 10 | -0.012 | 0.275 | 0.967 | 0.989 | 0.577 | 1.694 |
| Catenibacterium | IVW | 4 | -0.205 | 0.311 | 0.510 | 0.815 | 0.443 | 1.498 |
| Catenibacterium | MR Egger | 4 | 3.312 | 4.065 | 0.501 | 27.436 | 0.010 | >1000 |
| Catenibacterium | Weighted median | 4 | -0.269 | 0.314 | 0.392 | 0.764 | 0.413 | 1.414 |
| ChristensenellaceaeR.7group | IVW | 10 | 0.164 | 0.331 | 0.620 | 1.179 | 0.616 | 2.257 |
| ChristensenellaceaeR.7group | MR Egger | 10 | -0.234 | 1.054 | 0.830 | 0.791 | 0.100 | 6.239 |
| ChristensenellaceaeR.7group | Weighted median | 10 | -0.012 | 0.450 | 0.978 | 0.988 | 0.409 | 2.387 |
| Clostridiuminnocuumgroup | IVW | 9 | 0.034 | 0.181 | 0.851 | 1.035 | 0.725 | 1.476 |
| Clostridiuminnocuumgroup | MR Egger | 9 | 0.027 | 0.937 | 0.977 | 1.028 | 0.164 | 6.450 |
| Clostridiuminnocuumgroup | Weighted median | 9 | -0.189 | 0.227 | 0.404 | 0.828 | 0.531 | 1.291 |
| Clostridiumsensustricto1 | IVW | 7 | -0.104 | 0.362 | 0.774 | 0.901 | 0.443 | 1.834 |
| Clostridiumsensustricto1 | MR Egger | 7 | -0.486 | 1.006 | 0.649 | 0.615 | 0.086 | 4.416 |
| Clostridiumsensustricto1 | Weighted median | 7 | 0.165 | 0.427 | 0.699 | 1.179 | 0.511 | 2.721 |
| Collinsella | IVW | 8 | 0.384 | 0.368 | 0.297 | 1.468 | 0.714 | 3.020 |
| Collinsella | MR Egger | 8 | -1.284 | 1.377 | 0.387 | 0.277 | 0.019 | 4.113 |
| Collinsella | Weighted median | 8 | 0.441 | 0.436 | 0.312 | 1.554 | 0.661 | 3.653 |
| Coprobacter | IVW | 11 | -0.076 | 0.237 | 0.749 | 0.927 | 0.583 | 1.475 |
| Coprobacter | MR Egger | 11 | -1.241 | 0.890 | 0.197 | 0.289 | 0.050 | 1.655 |
| Coprobacter | Weighted median | 11 | 0.093 | 0.283 | 0.742 | 1.098 | 0.630 | 1.913 |
| Coprococcus1 | IVW | 12 | -0.168 | 0.272 | 0.537 | 0.845 | 0.496 | 1.441 |
| Coprococcus1 | MR Egger | 12 | -0.365 | 0.688 | 0.607 | 0.694 | 0.180 | 2.673 |
| Coprococcus1 | Weighted median | 12 | -0.377 | 0.363 | 0.299 | 0.686 | 0.336 | 1.398 |
| Coprococcus2 | IVW | 8 | 0.840 | 0.299 | 0.005 | 2.317 | 1.289 | 4.167 |
| Coprococcus2 | MR Egger | 8 | 0.545 | 1.730 | 0.764 | 1.724 | 0.058 | 51.169 |
| Coprococcus2 | Weighted median | 8 | 0.614 | 0.399 | 0.124 | 1.847 | 0.846 | 4.034 |
| Coprococcus3 | IVW | 9 | -0.426 | 0.350 | 0.224 | 0.653 | 0.329 | 1.297 |
| Coprococcus3 | MR Egger | 9 | -2.489 | 1.957 | 0.244 | 0.083 | 0.002 | 3.849 |
| Coprococcus3 | Weighted median | 9 | -0.240 | 0.458 | 0.600 | 0.786 | 0.320 | 1.930 |
| DefluviitaleaceaeUCG011 | IVW | 9 | 0.082 | 0.254 | 0.747 | 1.085 | 0.660 | 1.785 |
| DefluviitaleaceaeUCG011 | MR Egger | 9 | 0.242 | 0.970 | 0.810 | 1.274 | 0.190 | 8.528 |
| DefluviitaleaceaeUCG011 | Weighted median | 9 | 0.177 | 0.335 | 0.596 | 1.194 | 0.620 | 2.301 |
| Desulfovibrio | IVW | 10 | 0.101 | 0.246 | 0.683 | 1.106 | 0.682 | 1.792 |
| Desulfovibrio | MR Egger | 10 | -0.313 | 0.724 | 0.677 | 0.731 | 0.177 | 3.022 |
| Desulfovibrio | Weighted median | 10 | 0.201 | 0.343 | 0.557 | 1.223 | 0.625 | 2.393 |
| Dialister | IVW | 11 | 0.101 | 0.290 | 0.727 | 1.107 | 0.626 | 1.955 |
| Dialister | MR Egger | 11 | 0.367 | 1.253 | 0.776 | 1.443 | 0.124 | 16.834 |
| Dialister | Weighted median | 11 | 0.195 | 0.371 | 0.599 | 1.215 | 0.588 | 2.512 |
| Dorea | IVW | 10 | -0.395 | 0.339 | 0.244 | 0.673 | 0.346 | 1.309 |
| Dorea | MR Egger | 10 | -0.363 | 0.943 | 0.710 | 0.696 | 0.110 | 4.413 |
| Dorea | Weighted median | 10 | -0.204 | 0.470 | 0.664 | 0.815 | 0.324 | 2.049 |
| Eggerthella | IVW | 10 | 0.640 | 0.188 | 0.001 | 1.897 | 1.313 | 2.741 |
| Eggerthella | MR Egger | 10 | -0.459 | 0.867 | 0.611 | 0.632 | 0.116 | 3.456 |
| Eggerthella | Weighted median | 10 | 0.633 | 0.273 | 0.020 | 1.883 | 1.103 | 3.216 |
| Eisenbergiella | IVW | 11 | -0.271 | 0.190 | 0.154 | 0.763 | 0.525 | 1.107 |
| Eisenbergiella | MR Egger | 11 | 0.431 | 1.469 | 0.776 | 1.539 | 0.086 | 27.418 |
| Eisenbergiella | Weighted median | 11 | -0.409 | 0.251 | 0.103 | 0.664 | 0.406 | 1.087 |
| Enterorhabdus | IVW | 6 | -0.103 | 0.289 | 0.721 | 0.902 | 0.512 | 1.588 |
| Enterorhabdus | MR Egger | 6 | -0.063 | 0.764 | 0.938 | 0.939 | 0.210 | 4.194 |
| Enterorhabdus | Weighted median | 6 | -0.223 | 0.353 | 0.528 | 0.800 | 0.401 | 1.598 |
| Erysipelatoclostridium | IVW | 15 | -0.267 | 0.234 | 0.254 | 0.765 | 0.484 | 1.211 |
| Erysipelatoclostridium | MR Egger | 15 | 0.294 | 0.941 | 0.760 | 1.342 | 0.212 | 8.484 |
| Erysipelatoclostridium | Weighted median | 15 | -0.158 | 0.285 | 0.580 | 0.854 | 0.488 | 1.494 |
| ErysipelotrichaceaeUCG003 | IVW | 15 | -0.010 | 0.255 | 0.967 | 0.990 | 0.600 | 1.633 |
| ErysipelotrichaceaeUCG003 | MR Egger | 15 | -0.306 | 0.694 | 0.667 | 0.736 | 0.189 | 2.869 |
| ErysipelotrichaceaeUCG003 | Weighted median | 15 | 0.137 | 0.326 | 0.674 | 1.147 | 0.606 | 2.171 |
| Escherichia.Shigella | IVW | 10 | 0.566 | 0.293 | 0.053 | 1.762 | 0.993 | 3.127 |
| Escherichia.Shigella | MR Egger | 10 | -0.730 | 0.912 | 0.446 | 0.482 | 0.081 | 2.879 |
| Escherichia.Shigella | Weighted median | 10 | 0.529 | 0.383 | 0.167 | 1.698 | 0.802 | 3.595 |
| Eubacteriumbrachygroup | IVW | 10 | 0.175 | 0.164 | 0.286 | 1.192 | 0.864 | 1.644 |
| Eubacteriumbrachygroup | MR Egger | 10 | 0.763 | 0.661 | 0.282 | 2.144 | 0.586 | 7.837 |
| Eubacteriumbrachygroup | Weighted median | 10 | 0.215 | 0.228 | 0.347 | 1.239 | 0.792 | 1.939 |
| Eubacteriumcoprostanoligenesgroup | IVW | 14 | 0.291 | 0.287 | 0.311 | 1.338 | 0.762 | 2.347 |
| Eubacteriumcoprostanoligenesgroup | MR Egger | 14 | 1.646 | 1.130 | 0.171 | 5.185 | 0.566 | 47.494 |
| Eubacteriumcoprostanoligenesgroup | Weighted median | 14 | 0.385 | 0.380 | 0.312 | 1.469 | 0.698 | 3.093 |
| Eubacteriumeligensgroup | IVW | 6 | 0.394 | 0.390 | 0.313 | 1.482 | 0.690 | 3.183 |
| Eubacteriumeligensgroup | MR Egger | 6 | 2.624 | 1.456 | 0.146 | 13.785 | 0.794 | 239.386 |
| Eubacteriumeligensgroup | Weighted median | 6 | 0.578 | 0.504 | 0.251 | 1.783 | 0.664 | 4.787 |
| Eubacteriumfissicatenagroup | IVW | 9 | 0.142 | 0.222 | 0.522 | 1.152 | 0.746 | 1.779 |
| Eubacteriumfissicatenagroup | MR Egger | 9 | 1.474 | 1.120 | 0.230 | 4.365 | 0.486 | 39.188 |
| Eubacteriumfissicatenagroup | Weighted median | 9 | 0.119 | 0.249 | 0.634 | 1.126 | 0.691 | 1.835 |
| Eubacteriumhalliigroup | IVW | 15 | 0.063 | 0.236 | 0.789 | 1.065 | 0.671 | 1.690 |
| Eubacteriumhalliigroup | MR Egger | 15 | 0.384 | 0.493 | 0.450 | 1.468 | 0.559 | 3.857 |
| Eubacteriumhalliigroup | Weighted median | 15 | 0.259 | 0.328 | 0.429 | 1.296 | 0.681 | 2.465 |
| Eubacteriumnodatumgroup | IVW | 11 | -0.206 | 0.154 | 0.180 | 0.814 | 0.602 | 1.100 |
| Eubacteriumnodatumgroup | MR Egger | 11 | -0.340 | 0.720 | 0.648 | 0.712 | 0.174 | 2.918 |
| Eubacteriumnodatumgroup | Weighted median | 11 | -0.123 | 0.207 | 0.553 | 0.885 | 0.590 | 1.326 |
| Eubacteriumoxidoreducensgroup | IVW | 5 | -0.102 | 0.277 | 0.714 | 0.903 | 0.525 | 1.554 |
| Eubacteriumoxidoreducensgroup | MR Egger | 5 | 1.020 | 0.992 | 0.380 | 2.772 | 0.397 | 19.376 |
| Eubacteriumoxidoreducensgroup | Weighted median | 5 | -0.289 | 0.351 | 0.410 | 0.749 | 0.377 | 1.489 |
| Eubacteriumrectalegroup | IVW | 8 | -0.351 | 0.360 | 0.329 | 0.704 | 0.348 | 1.424 |
| Eubacteriumrectalegroup | MR Egger | 8 | 0.773 | 1.308 | 0.576 | 2.167 | 0.167 | 28.111 |
| Eubacteriumrectalegroup | Weighted median | 8 | -0.789 | 0.485 | 0.104 | 0.454 | 0.176 | 1.175 |
| Eubacteriumruminantiumgroup | IVW | 18 | -0.121 | 0.167 | 0.469 | 0.886 | 0.639 | 1.229 |
| Eubacteriumruminantiumgroup | MR Egger | 18 | 0.756 | 0.527 | 0.171 | 2.130 | 0.757 | 5.988 |
| Eubacteriumruminantiumgroup | Weighted median | 18 | 0.024 | 0.228 | 0.916 | 1.024 | 0.655 | 1.602 |
| Eubacteriumventriosumgroup | IVW | 15 | 0.329 | 0.376 | 0.381 | 1.390 | 0.665 | 2.904 |
| Eubacteriumventriosumgroup | MR Egger | 15 | 1.576 | 1.702 | 0.371 | 4.834 | 0.172 | 135.741 |
| Eubacteriumventriosumgroup | Weighted median | 15 | 0.378 | 0.392 | 0.335 | 1.459 | 0.677 | 3.143 |
| Eubacteriumxylanophilumgroup | IVW | 9 | 0.096 | 0.311 | 0.757 | 1.101 | 0.599 | 2.025 |
| Eubacteriumxylanophilumgroup | MR Egger | 9 | 0.025 | 0.991 | 0.981 | 1.025 | 0.147 | 7.157 |
| Eubacteriumxylanophilumgroup | Weighted median | 9 | -0.021 | 0.391 | 0.957 | 0.979 | 0.455 | 2.106 |
| Faecalibacterium | IVW | 10 | 0.057 | 0.269 | 0.833 | 1.058 | 0.624 | 1.794 |
| Faecalibacterium | MR Egger | 10 | 0.425 | 0.536 | 0.451 | 1.530 | 0.535 | 4.372 |
| Faecalibacterium | Weighted median | 10 | 0.166 | 0.369 | 0.653 | 1.180 | 0.573 | 2.432 |
| FamilyXIIIAD3011group | IVW | 13 | -0.513 | 0.265 | 0.053 | 0.599 | 0.356 | 1.007 |
| FamilyXIIIAD3011group | MR Egger | 13 | -0.474 | 1.266 | 0.715 | 0.623 | 0.052 | 7.447 |
| FamilyXIIIAD3011group | Weighted median | 13 | -0.208 | 0.335 | 0.535 | 0.812 | 0.421 | 1.566 |
| FamilyXIIIUCG001 | IVW | 8 | -0.639 | 0.308 | 0.038 | 0.528 | 0.289 | 0.964 |
| FamilyXIIIUCG001 | MR Egger | 8 | -0.600 | 0.956 | 0.553 | 0.549 | 0.084 | 3.572 |
| FamilyXIIIUCG001 | Weighted median | 8 | -0.758 | 0.381 | 0.047 | 0.469 | 0.222 | 0.988 |
| Flavonifractor | IVW | 5 | -0.137 | 0.361 | 0.704 | 0.872 | 0.430 | 1.769 |
| Flavonifractor | MR Egger | 5 | 0.822 | 1.438 | 0.607 | 2.275 | 0.136 | 38.091 |
| Flavonifractor | Weighted median | 5 | -0.004 | 0.455 | 0.994 | 0.996 | 0.408 | 2.432 |
| Fusicatenibacter | IVW | 18 | -0.061 | 0.251 | 0.809 | 0.941 | 0.575 | 1.539 |
| Fusicatenibacter | MR Egger | 18 | 0.806 | 0.940 | 0.404 | 2.238 | 0.355 | 14.114 |
| Fusicatenibacter | Weighted median | 18 | 0.040 | 0.337 | 0.904 | 1.041 | 0.538 | 2.015 |
| Gordonibacter | IVW | 12 | 0.182 | 0.192 | 0.343 | 1.199 | 0.824 | 1.746 |
| Gordonibacter | MR Egger | 12 | 0.155 | 0.864 | 0.862 | 1.167 | 0.215 | 6.345 |
| Gordonibacter | Weighted median | 12 | 0.070 | 0.222 | 0.750 | 1.073 | 0.695 | 1.656 |
| Haemophilus | IVW | 9 | -0.079 | 0.220 | 0.719 | 0.924 | 0.600 | 1.423 |
| Haemophilus | MR Egger | 9 | 0.317 | 0.494 | 0.542 | 1.373 | 0.521 | 3.619 |
| Haemophilus | Weighted median | 9 | -0.065 | 0.294 | 0.825 | 0.937 | 0.527 | 1.667 |
| Holdemanella | IVW | 11 | 0.178 | 0.227 | 0.433 | 1.195 | 0.766 | 1.865 |
| Holdemanella | MR Egger | 11 | 0.662 | 0.662 | 0.344 | 1.938 | 0.529 | 7.097 |
| Holdemanella | Weighted median | 11 | 0.006 | 0.277 | 0.981 | 1.006 | 0.584 | 1.734 |
| Holdemania | IVW | 14 | 0.070 | 0.203 | 0.730 | 1.073 | 0.721 | 1.596 |
| Holdemania | MR Egger | 14 | -0.023 | 0.596 | 0.970 | 0.977 | 0.304 | 3.142 |
| Holdemania | Weighted median | 14 | -0.034 | 0.279 | 0.902 | 0.966 | 0.559 | 1.671 |
| Howardella | IVW | 9 | -0.140 | 0.166 | 0.398 | 0.869 | 0.628 | 1.203 |
| Howardella | MR Egger | 9 | -0.420 | 0.695 | 0.564 | 0.657 | 0.168 | 2.564 |
| Howardella | Weighted median | 9 | -0.024 | 0.221 | 0.915 | 0.977 | 0.634 | 1.505 |
| Hungatella | IVW | 5 | 0.012 | 0.249 | 0.960 | 1.012 | 0.621 | 1.651 |
| Hungatella | MR Egger | 5 | -0.271 | 1.553 | 0.873 | 0.763 | 0.036 | 16.023 |
| Hungatella | Weighted median | 5 | 0.109 | 0.288 | 0.705 | 1.115 | 0.635 | 1.959 |
| Intestinibacter | IVW | 15 | -0.151 | 0.222 | 0.498 | 0.860 | 0.557 | 1.330 |
| Intestinibacter | MR Egger | 15 | 0.247 | 0.721 | 0.737 | 1.280 | 0.312 | 5.256 |
| Intestinibacter | Weighted median | 15 | 0.014 | 0.308 | 0.964 | 1.014 | 0.554 | 1.856 |
| Intestinimonas | IVW | 16 | 0.146 | 0.213 | 0.491 | 1.158 | 0.763 | 1.756 |
| Intestinimonas | MR Egger | 16 | 0.082 | 0.589 | 0.892 | 1.085 | 0.342 | 3.446 |
| Intestinimonas | Weighted median | 16 | 0.117 | 0.279 | 0.674 | 1.124 | 0.651 | 1.941 |
| Lachnoclostridium | IVW | 13 | -0.322 | 0.288 | 0.263 | 0.725 | 0.413 | 1.274 |
| Lachnoclostridium | MR Egger | 13 | -1.175 | 0.978 | 0.255 | 0.309 | 0.045 | 2.098 |
| Lachnoclostridium | Weighted median | 13 | -0.479 | 0.389 | 0.218 | 0.620 | 0.289 | 1.327 |
| Lachnospira | IVW | 5 | 0.299 | 0.473 | 0.527 | 1.349 | 0.534 | 3.407 |
| Lachnospira | MR Egger | 5 | 3.389 | 3.147 | 0.360 | 29.625 | 0.062 | >1000 |
| Lachnospira | Weighted median | 5 | 0.648 | 0.637 | 0.309 | 1.911 | 0.549 | 6.660 |
| LachnospiraceaeFCS020group | IVW | 12 | -0.193 | 0.257 | 0.453 | 0.825 | 0.499 | 1.364 |
| LachnospiraceaeFCS020group | MR Egger | 12 | -0.115 | 0.706 | 0.874 | 0.892 | 0.223 | 3.559 |
| LachnospiraceaeFCS020group | Weighted median | 12 | -0.293 | 0.348 | 0.399 | 0.746 | 0.377 | 1.475 |
| LachnospiraceaeNC2004group | IVW | 25 | 0.051 | 0.126 | 0.684 | 1.053 | 0.822 | 1.348 |
| LachnospiraceaeNC2004group | MR Egger | 25 | 0.759 | 0.711 | 0.297 | 2.135 | 0.530 | 8.610 |
| LachnospiraceaeNC2004group | Weighted median | 25 | 0.217 | 0.160 | 0.174 | 1.242 | 0.909 | 1.698 |
| LachnospiraceaeND3007group | IVW | 3 | -0.764 | 1.064 | 0.473 | 0.466 | 0.058 | 3.748 |
| LachnospiraceaeND3007group | MR Egger | 3 | -13.772 | 21.656 | 0.639 | <0.001 | <0.001 | >1000 |
| LachnospiraceaeND3007group | Weighted median | 3 | -1.431 | 0.820 | 0.081 | 0.239 | 0.048 | 1.194 |
| LachnospiraceaeNK4A136group | IVW | 15 | -0.469 | 0.264 | 0.076 | 0.626 | 0.373 | 1.051 |
| LachnospiraceaeNK4A136group | MR Egger | 15 | 0.294 | 0.491 | 0.560 | 1.341 | 0.513 | 3.510 |
| LachnospiraceaeNK4A136group | Weighted median | 15 | 0.086 | 0.356 | 0.810 | 1.090 | 0.542 | 2.190 |
| LachnospiraceaeUCG001 | IVW | 13 | -0.155 | 0.209 | 0.460 | 0.857 | 0.568 | 1.291 |
| LachnospiraceaeUCG001 | MR Egger | 13 | 0.374 | 0.873 | 0.677 | 1.453 | 0.263 | 8.042 |
| LachnospiraceaeUCG001 | Weighted median | 13 | -0.098 | 0.292 | 0.737 | 0.907 | 0.511 | 1.607 |
| LachnospiraceaeUCG004 | IVW | 12 | 0.104 | 0.286 | 0.717 | 1.109 | 0.633 | 1.942 |
| LachnospiraceaeUCG004 | MR Egger | 12 | 1.408 | 1.194 | 0.265 | 4.088 | 0.394 | 42.413 |
| LachnospiraceaeUCG004 | Weighted median | 12 | 0.132 | 0.380 | 0.727 | 1.142 | 0.543 | 2.402 |
| LachnospiraceaeUCG008 | IVW | 11 | 0.096 | 0.193 | 0.620 | 1.100 | 0.754 | 1.605 |
| LachnospiraceaeUCG008 | MR Egger | 11 | 0.306 | 0.991 | 0.764 | 1.359 | 0.195 | 9.468 |
| LachnospiraceaeUCG008 | Weighted median | 11 | 0.112 | 0.267 | 0.675 | 1.119 | 0.663 | 1.889 |
| LachnospiraceaeUCG010 | IVW | 10 | 0.236 | 0.298 | 0.429 | 1.266 | 0.706 | 2.272 |
| LachnospiraceaeUCG010 | MR Egger | 10 | 0.088 | 0.958 | 0.929 | 1.092 | 0.167 | 7.137 |
| LachnospiraceaeUCG010 | Weighted median | 10 | 0.127 | 0.410 | 0.757 | 1.135 | 0.509 | 2.533 |
| Lactobacillus | IVW | 9 | 0.455 | 0.196 | 0.020 | 1.576 | 1.073 | 2.315 |
| Lactobacillus | MR Egger | 9 | 0.909 | 0.516 | 0.122 | 2.481 | 0.902 | 6.824 |
| Lactobacillus | Weighted median | 9 | 0.587 | 0.263 | 0.025 | 1.799 | 1.075 | 3.011 |
| Lactococcus | IVW | 9 | -0.020 | 0.174 | 0.909 | 0.980 | 0.697 | 1.379 |
| Lactococcus | MR Egger | 9 | -0.224 | 0.781 | 0.782 | 0.799 | 0.173 | 3.693 |
| Lactococcus | Weighted median | 9 | 0.057 | 0.232 | 0.807 | 1.058 | 0.671 | 1.669 |
| Marvinbryantia | IVW | 10 | -0.308 | 0.286 | 0.282 | 0.735 | 0.419 | 1.288 |
| Marvinbryantia | MR Egger | 10 | 0.915 | 1.122 | 0.438 | 2.496 | 0.277 | 22.500 |
| Marvinbryantia | Weighted median | 10 | -0.223 | 0.365 | 0.541 | 0.800 | 0.392 | 1.635 |
| Methanobrevibacter | IVW | 6 | 0.018 | 0.219 | 0.933 | 1.019 | 0.663 | 1.564 |
| Methanobrevibacter | MR Egger | 6 | 0.944 | 0.814 | 0.311 | 2.570 | 0.521 | 12.668 |
| Methanobrevibacter | Weighted median | 6 | -0.268 | 0.290 | 0.355 | 0.765 | 0.433 | 1.351 |
| Odoribacter | IVW | 7 | -0.173 | 0.354 | 0.625 | 0.841 | 0.421 | 1.683 |
| Odoribacter | MR Egger | 7 | -0.269 | 1.109 | 0.818 | 0.764 | 0.087 | 6.720 |
| Odoribacter | Weighted median | 7 | -0.148 | 0.449 | 0.742 | 0.863 | 0.358 | 2.081 |
| Olsenella | IVW | 10 | -0.292 | 0.149 | 0.050 | 0.747 | 0.557 | 1.000 |
| Olsenella | MR Egger | 10 | -0.876 | 0.478 | 0.104 | 0.416 | 0.163 | 1.062 |
| Olsenella | Weighted median | 10 | -0.191 | 0.188 | 0.311 | 0.826 | 0.572 | 1.195 |
| Oscillibacter | IVW | 13 | -0.131 | 0.204 | 0.520 | 0.877 | 0.588 | 1.308 |
| Oscillibacter | MR Egger | 13 | -0.360 | 0.771 | 0.650 | 0.698 | 0.154 | 3.159 |
| Oscillibacter | Weighted median | 13 | -0.244 | 0.279 | 0.382 | 0.783 | 0.453 | 1.354 |
| Oscillospira | IVW | 7 | 0.285 | 0.316 | 0.369 | 1.329 | 0.715 | 2.472 |
| Oscillospira | MR Egger | 7 | 0.029 | 1.535 | 0.986 | 1.029 | 0.051 | 20.842 |
| Oscillospira | Weighted median | 7 | 0.033 | 0.395 | 0.934 | 1.033 | 0.476 | 2.241 |
| Oxalobacter | IVW | 11 | -0.078 | 0.156 | 0.618 | 0.925 | 0.681 | 1.256 |
| Oxalobacter | MR Egger | 11 | 0.337 | 0.743 | 0.661 | 1.401 | 0.326 | 6.011 |
| Oxalobacter | Weighted median | 11 | 0.137 | 0.214 | 0.524 | 1.146 | 0.753 | 1.745 |
| Parabacteroides | IVW | 5 | -0.602 | 0.479 | 0.209 | 0.548 | 0.214 | 1.402 |
| Parabacteroides | MR Egger | 5 | 3.581 | 2.724 | 0.280 | 35.918 | 0.173 | >1000 |
| Parabacteroides | Weighted median | 5 | -0.614 | 0.573 | 0.284 | 0.541 | 0.176 | 1.665 |
| Paraprevotella | IVW | 13 | 0.180 | 0.181 | 0.321 | 1.197 | 0.840 | 1.706 |
| Paraprevotella | MR Egger | 13 | -0.962 | 0.671 | 0.180 | 0.382 | 0.103 | 1.424 |
| Paraprevotella | Weighted median | 13 | -0.045 | 0.246 | 0.856 | 0.956 | 0.591 | 1.548 |
| Parasutterella | IVW | 14 | -0.006 | 0.207 | 0.977 | 0.994 | 0.662 | 1.492 |
| Parasutterella | MR Egger | 14 | -0.530 | 0.575 | 0.375 | 0.589 | 0.191 | 1.817 |
| Parasutterella | Weighted median | 14 | 0.009 | 0.285 | 0.976 | 1.009 | 0.577 | 1.762 |
| Peptococcus | IVW | 12 | 0.060 | 0.200 | 0.764 | 1.062 | 0.718 | 1.570 |
| Peptococcus | MR Egger | 12 | -0.713 | 0.769 | 0.375 | 0.490 | 0.109 | 2.211 |
| Peptococcus | Weighted median | 12 | 0.042 | 0.236 | 0.859 | 1.043 | 0.657 | 1.655 |
| Phascolarctobacterium | IVW | 10 | -0.361 | 0.266 | 0.175 | 0.697 | 0.414 | 1.175 |
| Phascolarctobacterium | MR Egger | 10 | -0.033 | 1.222 | 0.979 | 0.967 | 0.088 | 10.620 |
| Phascolarctobacterium | Weighted median | 10 | -0.538 | 0.364 | 0.140 | 0.584 | 0.286 | 1.193 |
| Prevotella7 | IVW | 11 | 0.059 | 0.147 | 0.689 | 1.060 | 0.795 | 1.414 |
| Prevotella7 | MR Egger | 11 | 0.447 | 0.866 | 0.618 | 1.564 | 0.287 | 8.533 |
| Prevotella7 | Weighted median | 11 | 0.151 | 0.207 | 0.466 | 1.163 | 0.775 | 1.746 |
| Prevotella9 | IVW | 15 | -0.288 | 0.191 | 0.131 | 0.750 | 0.516 | 1.090 |
| Prevotella9 | MR Egger | 15 | -0.827 | 0.557 | 0.162 | 0.437 | 0.147 | 1.303 |
| Prevotella9 | Weighted median | 15 | -0.228 | 0.258 | 0.377 | 0.796 | 0.480 | 1.320 |
| RikenellaceaeRC9gutgroup | IVW | 11 | -0.028 | 0.142 | 0.842 | 0.972 | 0.735 | 1.285 |
| RikenellaceaeRC9gutgroup | MR Egger | 11 | <0.001 | 0.887 | 1.000 | 1.000 | 0.176 | 5.695 |
| RikenellaceaeRC9gutgroup | Weighted median | 11 | 0.009 | 0.199 | 0.965 | 1.009 | 0.683 | 1.490 |
| Romboutsia | IVW | 13 | -0.134 | 0.257 | 0.602 | 0.875 | 0.529 | 1.446 |
| Romboutsia | MR Egger | 13 | -0.771 | 0.745 | 0.323 | 0.463 | 0.107 | 1.992 |
| Romboutsia | Weighted median | 13 | -0.060 | 0.345 | 0.862 | 0.942 | 0.478 | 1.853 |
| Roseburia | IVW | 14 | 0.222 | 0.284 | 0.436 | 1.248 | 0.715 | 2.179 |
| Roseburia | MR Egger | 14 | 0.272 | 0.894 | 0.766 | 1.313 | 0.228 | 7.573 |
| Roseburia | Weighted median | 14 | 0.630 | 0.386 | 0.102 | 1.878 | 0.882 | 3.999 |
| Ruminiclostridium5 | IVW | 11 | 0.272 | 0.349 | 0.435 | 1.313 | 0.663 | 2.601 |
| Ruminiclostridium5 | MR Egger | 11 | 1.304 | 1.495 | 0.406 | 3.683 | 0.197 | 69.030 |
| Ruminiclostridium5 | Weighted median | 11 | 0.070 | 0.453 | 0.878 | 1.072 | 0.441 | 2.604 |
| Ruminiclostridium6 | IVW | 15 | 0.197 | 0.243 | 0.417 | 1.218 | 0.757 | 1.959 |
| Ruminiclostridium6 | MR Egger | 15 | 0.987 | 0.598 | 0.123 | 2.682 | 0.831 | 8.659 |
| Ruminiclostridium6 | Weighted median | 15 | 0.380 | 0.329 | 0.248 | 1.462 | 0.767 | 2.785 |
| Ruminiclostridium9 | IVW | 8 | -0.487 | 0.388 | 0.209 | 0.614 | 0.287 | 1.314 |
| Ruminiclostridium9 | MR Egger | 8 | 0.157 | 1.987 | 0.940 | 1.170 | 0.024 | 57.421 |
| Ruminiclostridium9 | Weighted median | 8 | -0.061 | 0.487 | 0.900 | 0.941 | 0.362 | 2.445 |
| RuminococcaceaeNK4A214group | IVW | 13 | 0.130 | 0.327 | 0.690 | 1.139 | 0.600 | 2.162 |
| RuminococcaceaeNK4A214group | MR Egger | 13 | 0.188 | 1.127 | 0.871 | 1.206 | 0.132 | 10.987 |
| RuminococcaceaeNK4A214group | Weighted median | 13 | 0.649 | 0.394 | 0.100 | 1.913 | 0.883 | 4.143 |
| RuminococcaceaeUCG002 | IVW | 22 | -0.114 | 0.201 | 0.570 | 0.892 | 0.601 | 1.323 |
| RuminococcaceaeUCG002 | MR Egger | 22 | -0.579 | 0.531 | 0.289 | 0.561 | 0.198 | 1.587 |
| RuminococcaceaeUCG002 | Weighted median | 22 | -0.046 | 0.285 | 0.871 | 0.955 | 0.546 | 1.669 |
| RuminococcaceaeUCG003 | IVW | 12 | -0.484 | 0.259 | 0.062 | 0.616 | 0.371 | 1.024 |
| RuminococcaceaeUCG003 | MR Egger | 12 | -0.374 | 0.845 | 0.668 | 0.688 | 0.131 | 3.604 |
| RuminococcaceaeUCG003 | Weighted median | 12 | -0.379 | 0.340 | 0.265 | 0.685 | 0.352 | 1.333 |
| RuminococcaceaeUCG004 | IVW | 11 | -0.318 | 0.318 | 0.317 | 0.728 | 0.391 | 1.356 |
| RuminococcaceaeUCG004 | MR Egger | 11 | -1.989 | 1.764 | 0.289 | 0.137 | 0.004 | 4.347 |
| RuminococcaceaeUCG004 | Weighted median | 11 | 0.085 | 0.358 | 0.812 | 1.089 | 0.540 | 2.198 |
| RuminococcaceaeUCG005 | IVW | 14 | 0.012 | 0.240 | 0.961 | 1.012 | 0.632 | 1.619 |
| RuminococcaceaeUCG005 | MR Egger | 14 | -0.483 | 0.651 | 0.472 | 0.617 | 0.172 | 2.211 |
| RuminococcaceaeUCG005 | Weighted median | 14 | -0.127 | 0.317 | 0.688 | 0.880 | 0.473 | 1.639 |
| RuminococcaceaeUCG009 | IVW | 12 | -0.407 | 0.247 | 0.099 | 0.666 | 0.410 | 1.079 |
| RuminococcaceaeUCG009 | MR Egger | 12 | -0.341 | 1.033 | 0.748 | 0.711 | 0.094 | 5.386 |
| RuminococcaceaeUCG009 | Weighted median | 12 | -0.588 | 0.269 | 0.029 | 0.556 | 0.328 | 0.942 |
| RuminococcaceaeUCG010 | IVW | 6 | 0.054 | 0.356 | 0.880 | 1.055 | 0.525 | 2.121 |
| RuminococcaceaeUCG010 | MR Egger | 6 | -1.640 | 0.936 | 0.155 | 0.194 | 0.031 | 1.215 |
| RuminococcaceaeUCG010 | Weighted median | 6 | 0.223 | 0.472 | 0.636 | 1.250 | 0.496 | 3.155 |
| RuminococcaceaeUCG011 | IVW | 8 | 0.050 | 0.164 | 0.760 | 1.051 | 0.762 | 1.450 |
| RuminococcaceaeUCG011 | MR Egger | 8 | 0.072 | 0.820 | 0.933 | 1.074 | 0.215 | 5.364 |
| RuminococcaceaeUCG011 | Weighted median | 8 | 0.018 | 0.200 | 0.928 | 1.018 | 0.688 | 1.508 |
| RuminococcaceaeUCG013 | IVW | 12 | -0.414 | 0.282 | 0.142 | 0.661 | 0.380 | 1.149 |
| RuminococcaceaeUCG013 | MR Egger | 12 | -1.428 | 0.802 | 0.105 | 0.240 | 0.050 | 1.154 |
| RuminococcaceaeUCG013 | Weighted median | 12 | -0.209 | 0.374 | 0.577 | 0.812 | 0.390 | 1.691 |
| RuminococcaceaeUCG014 | IVW | 11 | -0.372 | 0.289 | 0.198 | 0.690 | 0.392 | 1.215 |
| RuminococcaceaeUCG014 | MR Egger | 11 | -0.539 | 0.716 | 0.470 | 0.583 | 0.143 | 2.370 |
| RuminococcaceaeUCG014 | Weighted median | 11 | -0.673 | 0.371 | 0.070 | 0.510 | 0.246 | 1.057 |
| Ruminococcus1 | IVW | 10 | 0.585 | 0.354 | 0.098 | 1.795 | 0.897 | 3.591 |
| Ruminococcus1 | MR Egger | 10 | 0.619 | 1.023 | 0.562 | 1.856 | 0.250 | 13.796 |
| Ruminococcus1 | Weighted median | 10 | 0.425 | 0.417 | 0.308 | 1.529 | 0.676 | 3.459 |
| Ruminococcus2 | IVW | 14 | -0.303 | 0.229 | 0.186 | 0.739 | 0.471 | 1.157 |
| Ruminococcus2 | MR Egger | 14 | -0.036 | 0.541 | 0.947 | 0.964 | 0.334 | 2.783 |
| Ruminococcus2 | Weighted median | 14 | -0.089 | 0.331 | 0.788 | 0.915 | 0.478 | 1.749 |
| Ruminococcusgauvreauiigroup | IVW | 13 | 0.121 | 0.248 | 0.627 | 1.128 | 0.694 | 1.835 |
| Ruminococcusgauvreauiigroup | MR Egger | 13 | -0.541 | 1.079 | 0.626 | 0.582 | 0.070 | 4.827 |
| Ruminococcusgauvreauiigroup | Weighted median | 13 | 0.236 | 0.348 | 0.498 | 1.266 | 0.640 | 2.502 |
| Ruminococcusgnavusgroup | IVW | 12 | 0.139 | 0.182 | 0.447 | 1.149 | 0.803 | 1.642 |
| Ruminococcusgnavusgroup | MR Egger | 12 | 0.735 | 0.860 | 0.413 | 2.085 | 0.386 | 11.260 |
| Ruminococcusgnavusgroup | Weighted median | 12 | 0.207 | 0.247 | 0.403 | 1.230 | 0.758 | 1.997 |
| Ruminococcustorquesgroup | IVW | 9 | 0.049 | 0.373 | 0.895 | 1.051 | 0.506 | 2.180 |
| Ruminococcustorquesgroup | MR Egger | 9 | 0.080 | 1.710 | 0.964 | 1.083 | 0.038 | 30.955 |
| Ruminococcustorquesgroup | Weighted median | 9 | 0.091 | 0.483 | 0.850 | 1.095 | 0.425 | 2.823 |
| Sellimonas | IVW | 9 | <0.001 | 0.145 | 0.999 | 1.000 | 0.753 | 1.328 |
| Sellimonas | MR Egger | 9 | 0.358 | 0.850 | 0.686 | 1.431 | 0.270 | 7.572 |
| Sellimonas | Weighted median | 9 | 0.072 | 0.204 | 0.725 | 1.074 | 0.721 | 1.602 |
| Senegalimassilia | IVW | 6 | -0.301 | 0.349 | 0.390 | 0.740 | 0.373 | 1.469 |
| Senegalimassilia | MR Egger | 6 | 0.145 | 1.608 | 0.932 | 1.156 | 0.049 | 27.015 |
| Senegalimassilia | Weighted median | 6 | 0.004 | 0.382 | 0.992 | 1.004 | 0.475 | 2.124 |
| Slackia | IVW | 6 | -0.144 | 0.267 | 0.589 | 0.866 | 0.513 | 1.461 |
| Slackia | MR Egger | 6 | 1.517 | 1.739 | 0.432 | 4.558 | 0.151 | 137.837 |
| Slackia | Weighted median | 6 | -0.076 | 0.340 | 0.823 | 0.927 | 0.476 | 1.804 |
| Streptococcus | IVW | 14 | -0.080 | 0.274 | 0.771 | 0.923 | 0.540 | 1.579 |
| Streptococcus | MR Egger | 14 | -1.116 | 0.994 | 0.284 | 0.328 | 0.047 | 2.299 |
| Streptococcus | Weighted median | 14 | 0.161 | 0.372 | 0.664 | 1.175 | 0.567 | 2.435 |
| Subdoligranulum | IVW | 11 | 0.221 | 0.314 | 0.480 | 1.248 | 0.675 | 2.308 |
| Subdoligranulum | MR Egger | 11 | 0.299 | 0.852 | 0.734 | 1.349 | 0.254 | 7.161 |
| Subdoligranulum | Weighted median | 11 | 0.484 | 0.419 | 0.248 | 1.622 | 0.714 | 3.688 |
| Sutterella | IVW | 12 | -0.157 | 0.326 | 0.631 | 0.855 | 0.451 | 1.621 |
| Sutterella | MR Egger | 12 | 0.007 | 1.480 | 0.996 | 1.007 | 0.055 | 18.311 |
| Sutterella | Weighted median | 12 | -0.172 | 0.379 | 0.650 | 0.842 | 0.400 | 1.771 |
| Terrisporobacter | IVW | 5 | -0.560 | 0.304 | 0.066 | 0.571 | 0.315 | 1.038 |
| Terrisporobacter | MR Egger | 5 | -0.503 | 0.982 | 0.644 | 0.605 | 0.088 | 4.147 |
| Terrisporobacter | Weighted median | 5 | -0.711 | 0.390 | 0.068 | 0.491 | 0.229 | 1.054 |
| Turicibacter | IVW | 9 | 0.125 | 0.239 | 0.599 | 1.134 | 0.710 | 1.811 |
| Turicibacter | MR Egger | 9 | 1.643 | 1.008 | 0.147 | 5.169 | 0.717 | 37.286 |
| Turicibacter | Weighted median | 9 | 0.326 | 0.312 | 0.296 | 1.385 | 0.752 | 2.553 |
| Tyzzerella3 | IVW | 13 | 0.060 | 0.172 | 0.730 | 1.061 | 0.757 | 1.488 |
| Tyzzerella3 | MR Egger | 13 | -0.233 | 1.033 | 0.826 | 0.792 | 0.105 | 6.005 |
| Tyzzerella3 | Weighted median | 13 | 0.149 | 0.225 | 0.506 | 1.161 | 0.748 | 1.804 |
| Veillonella | IVW | 6 | -0.106 | 0.448 | 0.813 | 0.899 | 0.374 | 2.163 |
| Veillonella | MR Egger | 6 | 3.411 | 3.542 | 0.390 | 30.281 | 0.029 | >1000 |
| Veillonella | Weighted median | 6 | -0.297 | 0.446 | 0.506 | 0.743 | 0.310 | 1.781 |
| Victivallis | IVW | 10 | 0.182 | 0.180 | 0.314 | 1.199 | 0.842 | 1.708 |
| Victivallis | MR Egger | 10 | -1.697 | 1.295 | 0.226 | 0.183 | 0.014 | 2.319 |
| Victivallis | Weighted median | 10 | 0.127 | 0.221 | 0.566 | 1.135 | 0.736 | 1.752 |

**Supplementary Table 3.** Reverse MR estimates for the association between gut microbiota and FM

| Bacterial taxa (outcome) | exposure | MR method | No. of SNP | Beta | Standard error | P-value | OR | Lower 95%CI | Upper 95%CI |
| --- | --- | --- | --- | --- | --- | --- | --- | --- | --- |
| Coprococcus2 | Fibromyalgia | IVW | 9 | -0.032 | 0.018 | 0.071 | 0.969 | 0.936 | 1.003 |
| Coprococcus2 | Fibromyalgia | MR Egger | 9 | -0.071 | 0.073 | 0.365 | 0.932 | 0.807 | 1.075 |
| Coprococcus2 | Fibromyalgia | Weighted median | 9 | -0.034 | 0.023 | 0.144 | 0.967 | 0.924 | 1.012 |
| Eggerthella | Fibromyalgia | IVW | 8 | -0.007 | 0.028 | 0.794 | 0.993 | 0.941 | 1.048 |
| Eggerthella | Fibromyalgia | MR Egger | 8 | 0.042 | 0.147 | 0.787 | 1.042 | 0.782 | 1.390 |
| Eggerthella | Fibromyalgia | Weighted median | 8 | 0.001 | 0.038 | 0.987 | 1.001 | 0.929 | 1.078 |
| Lactobacillus | Fibromyalgia | IVW | 8 | -0.021 | 0.024 | 0.379 | 0.979 | 0.935 | 1.026 |
| Lactobacillus | Fibromyalgia | MR Egger | 8 | 0.121 | 0.119 | 0.350 | 1.129 | 0.893 | 1.426 |
| Lactobacillus | Fibromyalgia | Weighted median | 8 | -0.003 | 0.033 | 0.922 | 0.997 | 0.934 | 1.063 |
| FamilyXIIIUCG001 | Fibromyalgia | IVW | 9 | 0.008 | 0.017 | 0.616 | 1.008 | 0.976 | 1.042 |
| FamilyXIIIUCG001 | Fibromyalgia | MR Egger | 9 | -0.093 | 0.072 | 0.234 | 0.911 | 0.791 | 1.048 |
| FamilyXIIIUCG001 | Fibromyalgia | Weighted median | 9 | 0.006 | 0.021 | 0.791 | 1.006 | 0.965 | 1.048 |
| Olsenella | Fibromyalgia | IVW | 8 | 0.006 | 0.037 | 0.879 | 1.006 | 0.935 | 1.082 |
| Olsenella | Fibromyalgia | MR Egger | 8 | 0.193 | 0.189 | 0.346 | 1.213 | 0.838 | 1.757 |
| Olsenella | Fibromyalgia | Weighted median | 8 | 0.016 | 0.045 | 0.720 | 1.016 | 0.931 | 1.109 |

**Supplementary Table 4.** The Cochran's IVW Q test of gut microbiota instrumental variables

| outcome | Bacterial taxa (exposure) | MR method | Q | df | P-value |
| --- | --- | --- | --- | --- | --- |
| Fibromyalgia | Coprococcus2 | MR Egger | 3.424 | 6 | 0.754 |
| Fibromyalgia | Coprococcus2 | Inverse variance weighted | 3.454 | 7 | 0.840 |
| Fibromyalgia | Eggerthella | MR Egger | 5.329 | 8 | 0.722 |
| Fibromyalgia | Eggerthella | Inverse variance weighted | 7.016 | 9 | 0.635 |
| Fibromyalgia | Lactobacillus | MR Egger | 4.729 | 7 | 0.693 |
| Fibromyalgia | Lactobacillus | Inverse variance weighted | 5.631 | 8 | 0.688 |
| Fibromyalgia | FamilyXIIIUCG001 | MR Egger | 3.401 | 6 | 0.757 |
| Fibromyalgia | FamilyXIIIUCG001 | Inverse variance weighted | 3.403 | 7 | 0.845 |
| Fibromyalgia | Olsenella | MR Egger | 2.415 | 8 | 0.966 |
| Fibromyalgia | Olsenella | Inverse variance weighted | 4.073 | 9 | 0.907 |

**Supplementary Table 5.** Pleiotropy assessed by intercept term in MR Egger regression of the association between gut microbiota and FM.

| outcome | Bacterial taxa (exposure) | Egger_intercept | SE | P-value |
| --- | --- | --- | --- | --- |
| Fibromyalgia | Coprococcus2 | 0.023 | 0.135 | 0.868 |
| Fibromyalgia | Eggerthella | 0.124 | 0.095 | 0.230 |
| Fibromyalgia | Lactobacillus | -0.057 | 0.060 | 0.374 |
| Fibromyalgia | FamilyXIIIUCG001 | -0.003 | 0.080 | 0.967 |
| Fibromyalgia | Olsenella | 0.084 | 0.065 | 0.234 |

**Supplementary Table 6.** MR-PRESSO analysis for the association between gut microbiota and FM

| Main.MR.results.Exposure | Main.MR.results.MR.Analysis | Main.MR.results.Causal.Estimate | Main.MR.results.Sd | Main.MR.results.T.stat | Main.MR.results.P.value | MR.PRESSO.results.Global.Test.RSSobs | MR.PRESSO.results.Global.Test.Pvalue |
| --- | --- | --- | --- | --- | --- | --- | --- |
| beta.exposure | Raw | 0.840 | 0.210 | 3.996 | 0.005 | 4.461 | 0.866 |
| beta.exposure | Outlier-corrected | NA | NA | NA | NA | 4.461 | 0.866 |
| beta.exposure | Raw | 0.640 | 0.166 | 3.860 | 0.004 | 8.729 | 0.687 |
| beta.exposure | Outlier-corrected | NA | NA | NA | NA | 8.729 | 0.687 |
| beta.exposure | Raw | 0.455 | 0.164 | 2.766 | 0.024 | 6.839 | 0.702 |
| beta.exposure | Outlier-corrected | NA | NA | NA | NA | 6.839 | 0.702 |
| beta.exposure | Raw | -0.639 | 0.215 | -2.981 | 0.020 | 4.517 | 0.860 |
| beta.exposure | Outlier-corrected | NA | NA | NA | NA | 4.517 | 0.860 |
| beta.exposure | Raw | -0.292 | 0.100 | -2.909 | 0.017 | 5.371 | 0.895 |
| beta.exposure | Outlier-corrected | NA | NA | NA | NA | 5.371 | 0.895 |

**Supplementary Table 7.** The F-statistics of gut microbiota instrumental variables

| Bacterial taxa (exposure) | SNP | Beta | Samplesize | MAF | R2 | F |
| --- | --- | --- | --- | --- | --- | --- |
| Coprococcus2 | rs59936925 | 0.117 | 14306 | 0.042 | 0.001 | 15.726 |
| Coprococcus2 | rs6677933 | -0.080 | 14306 | 0.210 | 0.002 | 30.782 |
| Coprococcus2 | rs1958519 | 0.067 | 14306 | 0.257 | 0.002 | 24.245 |
| Coprococcus2 | rs72680320 | -0.065 | 14306 | 0.219 | 0.001 | 20.654 |
| Coprococcus2 | rs2482516 | 0.075 | 14306 | 0.198 | 0.002 | 25.872 |
| Coprococcus2 | rs35890118 | -0.067 | 14306 | 0.286 | 0.002 | 25.907 |
| Coprococcus2 | rs9426473 | 0.073 | 14306 | 0.225 | 0.002 | 26.437 |
| Coprococcus2 | rs6894272 | -0.113 | 14306 | 0.069 | 0.002 | 23.573 |
| Coprococcus2 | rs12634070 | 0.074 | 14306 | 0.186 | 0.002 | 23.560 |
| Coprococcus2 | rs61823518 | -0.096 | 14306 | 0.095 | 0.002 | 22.522 |
| Eggerthella | rs3851328 | -0.108 | 14306 | 0.250 | 0.004 | 62.554 |
| Eggerthella | rs2240838 | 0.098 | 14306 | 0.490 | 0.005 | 69.074 |
| Eggerthella | rs2223081 | 0.103 | 14306 | 0.271 | 0.004 | 59.779 |
| Eggerthella | rs112205261 | -0.189 | 14306 | 0.082 | 0.005 | 77.301 |
| Eggerthella | rs4985746 | 0.111 | 14306 | 0.202 | 0.004 | 56.578 |
| Eggerthella | rs1784405 | 0.091 | 14306 | 0.481 | 0.004 | 59.155 |
| Eggerthella | rs76663501 | 0.175 | 14306 | 0.065 | 0.004 | 53.967 |
| Eggerthella | rs6430926 | 0.088 | 14306 | 0.498 | 0.004 | 55.566 |
| Eggerthella | rs2877457 | -0.093 | 14306 | 0.212 | 0.003 | 41.825 |
| Eggerthella | rs13070736 | -0.121 | 14306 | 0.158 | 0.004 | 56.141 |
| Eggerthella | rs67490567 | 0.108 | 14306 | 0.195 | 0.004 | 53.034 |
| Lactobacillus | rs921925 | 0.099 | 14306 | 0.281 | 0.004 | 56.275 |
| Lactobacillus | rs16861661 | -0.183 | 14306 | 0.065 | 0.004 | 58.529 |
| Lactobacillus | rs768253 | -0.079 | 14306 | 0.423 | 0.003 | 43.917 |
| Lactobacillus | rs11674854 | -0.085 | 14306 | 0.406 | 0.004 | 50.320 |
| Lactobacillus | rs328312 | 0.082 | 14306 | 0.470 | 0.003 | 47.503 |
| Lactobacillus | rs6092149 | -0.080 | 14306 | 0.302 | 0.003 | 38.832 |
| Lactobacillus | rs75127669 | 0.140 | 14306 | 0.022 | 0.001 | 11.823 |
| Lactobacillus | rs77478751 | -0.220 | 14306 | 0.046 | 0.004 | 61.570 |
| Lactobacillus | rs1530559 | 0.080 | 14306 | 0.456 | 0.003 | 46.028 |
| Lactobacillus | rs62314653 | 0.188 | 14306 | 0.058 | 0.004 | 55.341 |
| Lactobacillus | rs7399658 | -0.107 | 14306 | 0.088 | 0.002 | 26.354 |
| Lactobacillus | rs12693845 | -0.081 | 14306 | 0.352 | 0.003 | 42.448 |
| FamilyXIIIUCG001 | rs1426266 | -0.067 | 14306 | 0.319 | 0.002 | 27.566 |
| FamilyXIIIUCG001 | rs12049454 | -0.065 | 14306 | 0.292 | 0.002 | 24.816 |
| FamilyXIIIUCG001 | rs3842897 | -0.113 | 14306 | 0.079 | 0.002 | 26.394 |
| FamilyXIIIUCG001 | rs116979587 | -0.122 | 14306 | 0.059 | 0.002 | 23.564 |
| FamilyXIIIUCG001 | rs62414802 | -0.061 | 14306 | 0.266 | 0.001 | 20.926 |
| FamilyXIIIUCG001 | rs2276529 | -0.076 | 14306 | 0.212 | 0.002 | 27.575 |
| FamilyXIIIUCG001 | rs112362903 | -0.149 | 14306 | 0.039 | 0.002 | 23.884 |
| FamilyXIIIUCG001 | rs8076666 | 0.089 | 14306 | 0.119 | 0.002 | 23.549 |
| FamilyXIIIUCG001 | rs7119679 | -0.081 | 14306 | 0.177 | 0.002 | 27.327 |
| FamilyXIIIUCG001 | rs76463770 | 0.193 | 14306 | 0.043 | 0.003 | 44.205 |
| Olsenella | rs1035588 | -0.108 | 14306 | 0.360 | 0.005 | 77.509 |
| Olsenella | rs62112538 | -0.199 | 14306 | 0.070 | 0.005 | 74.457 |
| Olsenella | rs35225860 | -0.224 | 14306 | 0.050 | 0.005 | 68.266 |
| Olsenella | rs72691585 | -0.249 | 14306 | 0.060 | 0.007 | 100.807 |
| Olsenella | rs9460691 | 0.120 | 14306 | 0.240 | 0.005 | 75.494 |
| Olsenella | rs17148768 | 0.140 | 14306 | 0.200 | 0.006 | 90.845 |
| Olsenella | rs61090148 | -0.105 | 14306 | 0.410 | 0.005 | 76.386 |
| Olsenella | rs7540303 | 0.108 | 14306 | 0.370 | 0.005 | 78.271 |
| Olsenella | rs2759329 | -0.111 | 14306 | 0.390 | 0.006 | 84.550 |
| Olsenella | rs6046522 | 0.123 | 14306 | 0.330 | 0.007 | 96.241 |
| Olsenella | rs8066522 | -0.107 | 14306 | 0.350 | 0.005 | 74.242 |
